# Monitoring correlates of SARS-CoV-2 infection in cell culture using a two-photon-active calcium-sensitive dye

**DOI:** 10.1186/s11658-024-00619-0

**Published:** 2024-07-19

**Authors:** Domokos Máthé, Gergely Szalay, Levente Cseri, Zoltán Kis, Bernadett Pályi, Gábor Földes, Noémi Kovács, Anna Fülöp, Áron Szepesi, Polett Hajdrik, Attila Csomos, Ákos Zsembery, Kristóf Kádár, Gergely Katona, Zoltán Mucsi, Balázs József Rózsa, Ervin Kovács

**Affiliations:** 1 Department of Biophysics and Radiation Biology, Semmelweis University, Tűzoltó utca 37-47, 1094 Budapest, Hungary; 2 In Vivo Imaging Advanced Core Facility, Hungarian Centre of Excellence for Molecular Medicine, Tűzoltó utca 37-47, 1094 Budapest, Hungary; 3 HUN-REN Physical Virology Research Group, Semmelweis University, Tűzoltó utca 37-47, 1094 Budapest, Hungary; 4 Laboratory of 3D Functional Network and Dendritic Imaging, HUN-REN Institute of Experimental Medicine, Szigony utca 43, 1083 Budapest, Hungary; 5 BrainVisionCenter, Liliom utca 43-45, 1094 Budapest, Hungary; 6 Femtonics Ltd., Tűzoltó utca 59, 1094 Budapest, Hungary; 7 National Center for Public Health, Albert Flórián út 2-6, 1097 Budapest, Hungary; 8 National Heart and Lung Institute, Imperial College London, Du Cane Road, London, W12 0NN UK; 9 Heart and Vascular Center, Semmelweis University, Városmajor utca. 68, 1122 Budapest, Hungary; 10 Hevesy György PhD School of Chemistry, Eötvös Loránd University, Pázmány Péter sétány 1/A, 1117 Budapest, Hungary; 11 Department of Oral Biology, Faculty of Dentistry, Semmelweis University, Nagyvárad tér 4, 1089 Budapest, Hungary; 12 Two-Photon Measurement Technology Group, The Faculty of Information Technology, Pázmány Péter Catholic University, Szigony utca 50/A, 1083 Budapest, Hungary; 13 Institute of Chemistry, Faculty of Materials Science and Engineering, University of Miskolc, Egyetem tér 1, 3515 Miskolc, Hungary; 14 Institute of Materials and Environmental Chemistry, HUN-REN Research Centre for Natural Sciences, Magyar Tudósok körútja 2, 1117 Budapest, Hungary

**Keywords:** Viral infections, SARS-CoV-2, Two-photon microscopy, Calcium sensors, Fluorescence imaging

## Abstract

**Background:**

The organism-wide effects of severe acute respiratory syndrome coronavirus 2 (SARS-CoV-2) viral infection are well studied, but little is known about the dynamics of how the infection spreads in time among or within cells due to the scarcity of suitable high-resolution experimental systems. It has been reported that SARS-CoV-2 infection pathways converge at calcium influx and subcellular calcium distribution changes. Imaging combined with a proper staining technique is an effective tool for studying subcellular calcium-related infection and replication mechanisms at such resolutions.

**Methods:**

Using two-photon (2P) fluorescence imaging with our novel Ca-selective dye, automated image analysis and clustering analysis were applied to reveal titer and variant effects on SARS-CoV-2-infected Vero E6 cells.

**Results:**

The application of a new calcium sensor molecule is shown, combined with a high-end 2P technique for imaging and identifying the patterns associated with cellular infection damage within cells. Vero E6 cells infected with SARS-CoV-2 variants, D614G or B.1.1.7, exhibit elevated cytosolic calcium levels, allowing infection monitoring by tracking the cellular changes in calcium level by the internalized calcium sensor. The imaging provides valuable information on how the level and intracellular distribution of calcium are perturbed during the infection. Moreover, two-photon calcium sensing allowed the distinction of infections by two studied viral variants via cluster analysis of the image parameters. This approach will facilitate the study of cellular correlates of infection and their quantification depending on viral variants and viral load.

**Conclusions:**

We propose a new two-photon microscopy-based method combined with a cell-internalized sensor to quantify the level of SARS-CoV-2 infection. We optimized the applied dye concentrations to not interfere with viral fusion and viral replication events. The presented method ensured the proper monitoring of viral infection, replication, and cell fate. It also enabled distinguishing intracellular details of cell damage, such as vacuole and apoptotic body formation. Using clustering analysis, 2P microscopy calcium fluorescence images were suitable to distinguish two different viral variants in cell cultures. Cellular harm levels read out by calcium imaging were quantitatively related to the initial viral multiplicity of infection numbers. Thus, 2P quantitative calcium imaging might be used as a correlate of infection or a correlate of activity in cellular antiviral studies.

**Graphical Abstract:**

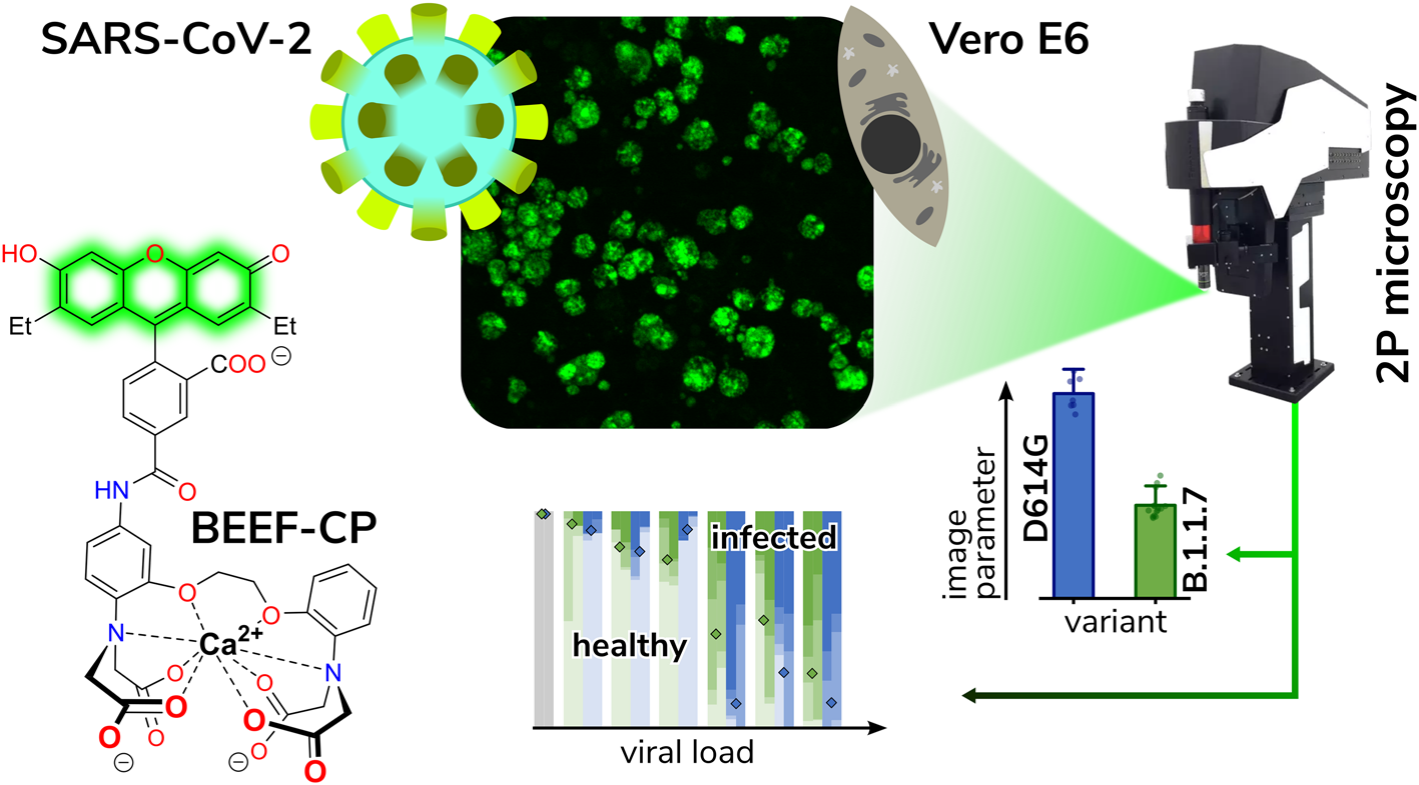

**Supplementary Information:**

The online version contains supplementary material available at 10.1186/s11658-024-00619-0.

## Background

The latest global epidemic—caused by the novel severe acute respiratory syndrome coronavirus 2 (SARS-CoV-2)—has highlighted the lack of in vivo experimental techniques for commensurable methods that could enable the monitoring of viral infections at cellular or subcellular levels [1]. In its first four years (as of 23 April 2024), the pandemic caused more than 774 million confirmed cases and about 7.0 million deaths [2]. Based on current trends, the disease is not expected to disappear anytime soon due to the appearance of successful virus variants that increasingly evade existing immunization, such as the Delta variant or the worldwide surge of different Omicron variants over the past years [3, 4]. Nonetheless, even without acute infections, clinical and experimental evidence shows that long-term effects of SARS-CoV-2 infection, sometimes referred to as post-acute sequelae of coronavirus disease 2019 (COVID-19) or, more colloquially, the long COVID or post-COVID syndrome, are affecting many people and are scarcely scrutinized [5]. With increasing proportions of infected people suffering from post-COVID and repeated reinfections, and the need to provide further therapies against these, from a translational medical perspective, a quantitative cellular harm assay remains a necessity [6, 7]. It is to be established as a routine way to quickly determine robust, standardizable cellular correlates of the SARS-CoV-2 infection. This “damage assay” should be performed quantitatively on relevant models, such as human tissue organoids or certain cell cultures widely used for SARS-CoV-2 studies.

We considered that a common pathway of harm in SARS-CoV-2 infection is cellular calcium ion homeostasis disruption. Since the start of research on SARS-CoV-2 viral entry, multiple mechanisms have been identified. Two of the most important effectors of harm, cell-to-cell spread via cellular fusion by the S2 subunit of the spike (S) protein and fusogenic peptide hexamer formation, and ACE2 downstream action via the calcineurin pathway are both dependent on Ca^2+^ ions [8–10]. Other results also highlight a connection between calcium homeostasis and SARS-CoV-2 infection [11]. It has been well established previously for many viruses that calcium is essential for viral entry into host cells, viral gene expression, processing of viral proteins, viral maturation, and release [11, 12]. Thus, these viruses either create their own calcium channel proteins, also known as viroporins, or hijack host-encoded cellular calcium channels to increase intracellular Ca^2+^ ion concentration. This can occur both by calcium influx from the extracellular space and release from the endoplasmic reticulum [13]. Studies revealed that the viroporins of coronaviruses, such as ORF3a or most notably the E protein, can transport Ca^2+^ ions into the cytoplasm, can activate the NLRP3 inflammasome, and are involved in virulence [11, 12]. Recent studies with S protein pseudotyped lentiviruses showed that the depletion of extracellular or intracellular Ca^2+^ ion concentration drastically decreased SARS-CoV-2 viral entry [14, 15]. It has also been highlighted that the calcium ion plays a crucial role in several steps of viral replication and protein synthesis in the cell, at a molecular level [16, 17]. These data undoubtedly reveal the role of the calcium ion in the course of COVID-19 as a disease and immunity, too [10]. As free calcium concentration is typically low in the cytoplasm (~ 100 nM), but it is stored in large amounts in intracellular vesicles, endoplasmic reticulum (ER), and Golgi lumens (0.1–1 mM) [18], a Ca^2+^ sensor with prime cell membrane permeability is needed to be able to closely monitor these processes in the living cells.

Tracking and visualizing the spread of SARS-CoV-2 infection in cellular and organoid systems has been indispensable in several important studies (Supplementary Table 4) [19, 20]. In antiviral drug efficacy experiments, real-time quantitative polymerase chain reaction (PCR) has been the go-to method of infection monitoring [21, 22]. While this method can indicate the overall viral RNA concentration in the extracellular medium, it does not provide any information about the state of individual cells. To visualize the infection in individual cells and thus allow microscopic imaging, fluorescent or immunofluorescent (IF) staining has been applied in cell cultures and organoids [23, 24]. However, IF staining requires fixation of the cells, which prevents continuous monitoring of the infection process. Furthermore, the meticulous sample preparation and the expensive staining reagents limit its practical applicability. In their interesting approach to developing a novel cell culture system model, Ju et al. replaced the sequence encoding the nucleocapsid (N) protein with that of green fluorescent protein (GFP) [25]. This genetic modification not only decreased the biosafety level (BSL) of the model system but also allowed the fluorescent detection and imaging of the infected cells. Nevertheless, this staining approach involving genetic engineering remains limited to such model systems, and even there, the GFP sequence is deleted from the genome over time as demonstrated by serial passage experiments [25].

Herein, we report a straightforward downstream-effector-oriented procedure that exploits the disturbance of cellular Ca^2+^ physiology by SARS-CoV-2 through the application of a novel Ca^2+^-sensitive dye. The dye is aimed to be more sensitive and less disturbing to the cellular environments than previously applied ones. Most of the known fluorescent Ca^2+^ ion sensors, especially those with BAPTA chelator functionality [26], suffer from certain limitations in terms of their cellular applications. First, the widely used acetoxymethyl ester (AM) protected derivatives need to be applied at high concentrations (in 1–2 mM) to generate the active, deprotected form at suitable concentrations for the assay measurements. The use of lower (50–100 µM) concentrations of the same dyes requires meticulous preparation, loading time restrictions, and recovery periods with continuous checks of the culture. The deprotection of the AM ester releases a toxic byproduct, formaldehyde, in substantial concentration during its hydrolysis, which may lead to cell damage or death, thus perturbing the cell viability assay [27]. Second, these commonly used dyes are practically membrane impermeable in their unprotected forms [28], such as Oregon Green (2′,7′-difluorofluorescein). It is thus desirable to develop sensor dye molecules with a more robust application profile, especially due to the specific working time considerations that come with cellular imaging in biosafety laboratories. To avoid these issues of the AM esters and to find a good compromise between activity and cell internalization, we aimed to design a novel Ca^2+^ ion sensitive sensor molecule, BAPTA-enabled diethyl-fluorescein-calcium probe (BEEF-CP). This compound features two nonpolar and electron-donating ethyl groups on the xanthene moiety, presumably contributing to its internalization and eliminating the need for esters that generate cytotoxic compounds upon activation [27]. Using fluorescence information in a previously-not-achieved high-sensitivity manner, one might be able to collect relevant data about many stages of the infection process. The infection of individual cells and cell cultures indicated by the fluorescent turn-on of the dye is monitored by high-resolution two-photon (2P) microscopy, which is capable of extracting information with high temporal and spatial resolution from different planes of cell cultures[29] or even three-dimensional organoids.

## Methods

### Two-photon (2P) imaging

All experiments were performed on a Femto Smart 2P microscope (Femtonics Ltd., Budapest, Hungary). Laser pulses were generated by a Mai Tai HP laser (SpectraPhysics, Santa Clara, CA), and the laser wavelength was tuned between 700 and 1050 nm, depending on the experiment. Laser intensity was controlled using a Pockels-cell (PC) electro-optical modulator (model 350-80 LA; Conoptics) after beam expansion (1:2, Thorlabs). For excitation and signal collection, a XLUMPLFLN20XW lens (Olympus, 20×, NA 1.0) was used, separated using a dichroic mirror (700dcxru, Chroma Technology) before the two-channel detector unit, which was sitting on the objective arm (traveling detector system) as described in detail elsewhere [30, 31]. Distilled water was used as immersion liquid. Emitted light entering the detectors was filtered with emission filters, ET525/100 for the green channel (Chroma Technology, Bellow Falls,VT). The fluorescence signal was collected to GaAsP photomultiplier tubes (PMT) fixed on the objective arm (H7422P-40-MOD, Hamamatsu). A set galvanometric mirrors was used to acquire full field images. All acquired images were recorded at 1000 × 1000 pixel resolution, spatial resolution was 0.36 µm/pixel. Single pixel integration time was 6.67 μs, resulting in a 6.7 s image acquisition time. The PMT’s maximal amplification voltage (~ 300 V) and the imaging laser power (~ 50 mW) were kept constant through all measurements and for all wavelengths for a more reliable comparison between the acquired fluorescent images. The dynamic range of the PMT is 16 bit. We selected the PMT’s gain to obtain most of the range but have a maximum of 50% of the pixels dark on the dimmest and no saturated pixel on the brightest image.

For the wavelength calibration measurements (Supplementary Fig. 3), we acquired a series of images using different stimulation wavelengths to calibrate the optimal laser wavelength. To compensate for the change in the output laser power experienced when tuning the output of the pulse laser, the PC amplification was aligned individually at every different wavelength tested, to keep laser intensity reaching the sample constant at ~ 50 mW power. The amplification levels were calibrated prior to the acquisition with an intensity meter placed under the objective. Note that the transmission of the objective is also dependent on the laser intensity, i.e., measuring the intensity under the objective also eliminates this error, besides the laser output modulation. Furthermore, not just the transmission but the material dispersion of the objective is wavelength dependent. Temporarily elongated laser pulses can also affect the two-photon efficacy. To minimize this effect, we used a glass stand chamber well plate, where the effect of the material dispersion is minimal. The maximum calculated change in the elongation of the pulse, caused by the material dispersion of the plate’s material, when tuning the laser from 700 to 1050 nm, is 21 fs. Due to the quadratic nature of the two-photon power on the excitation, and because the full pulse length is still below 200 fs at 1050 nm (~ 187 fs, FWHM—full width at half maximum), pulse elongation has less than 1% effect on the stimulation quantum efficacy. This is much lower than the effects we demonstrated and falls well within its noise level; therefore, we took no count for this effect during the analysis of the wavelength optimization.

For the image acquisition from the 96-well plate, one to three high-resolution images were acquired from every well using 700 nm stimulation The stage was moved between the plates automatically, with the known horizontal and vertical distances between the wells; however, the field of view and the focus were selected manually. For the imaging field, the center of the well was selected. If multiple images were taken, we moved the field just as much, so there was no overlap between the fields. The well plate was kept at room temperature during image acquisition with no CO_2_ supplementation. For one plate, the images from all wells were collected within 4 h. Images were saved as multichannel tiffs (for the dual-wavelength detection). Image acquisition was performed with a custom Matlab code. At each field, only one image was taken to minimize the phototoxic effect of the laser (although we did not see a phototoxic effect even after acquiring 30 images from the same imaging field; Supplementary Fig. 4). Raw two-photon microscopy images can be found in Supplementary Dataset 1 [32].

### Virus categories during manual analysis

Cells were manually localized in the recorded images, and then individual cells were visually categorized into five groups according to the following criteria:

- Healthy: Healthy cells display a pale, low-level fluorescence, due to low intracellular free Ca^2+^ level, and their shape is regular. The distribution of cells is homogeneous, and their arrangement on the plate is normal.
- Viral entry: These cells clearly show bright spot(s) in certain areas localized near the membrane, indicating an initial infection accompanied by the increase of the intracellular Ca^2+^ level.
- Viral replication: These cells exhibit bright fluorescence, due to the increased Ca^2+^ level, and they show signs of irregularities across their whole cell body, which remains compact. Their mean brightness is at least four times higher compared with healthy cells.
- Vacuolar lysis: Dying cells displaying bright fluorescence in their integrity and showing obvious disintegration were selected in this category. Due to the apoptosis-like disintegration of cells, bright fragments appear nearby the original cell, which were considered part of the initial cell and not counted separately.
- Absence of cells: Frames in samples with high viral titer contained significantly more dark areas. Cells disappeared from the spots where they were initially cultured. The difference between the mean cell count in noninfected frames and the actual cell count of the frame was considered indicative of the number of dead cells.

Cells belonging into each category were counted manually by the operator performing the analysis without direct information about the image position on the well plate, i.e., without information about the virus titer, variant, or dye concentration corresponding to the image.

### Automatic analysis

An ImageJ [33] macro was created to load PMT normalized images sequentially, run a despeckle filter to remove noise, run a threshold command to select the signal, measure the mean, max, and relative area of the selection, measure the mean intensity of the whole image (including empty space), and finally create a selection based on the threshold. To quantitate particle texture and roughness, a copy was made, and a Gaussian blur was applied. The converted image set was then subtracted from the original to enhance any variations in the image. Threshold areas in these images were then selected as before to create a second mask, which was again analyzed. Seven parameters characterizing the detected particles on the images were defined for each image: (i) relative signal area, (ii) image mean intensity, (iii) mean of the threshold area, (iv) maximum particle intensity, (v) average particle size, (vi) particle percentage area, and (vii) particle mean intensity. *t*-Distributed stochastic neighbor embedding (t-SNE) 2D plot was obtained in MATLAB using the built-in “tsne” function with default random number generation, a perplexity value of 10, and exaggeration value of 50 on all seven parameters of images acquired with 0.5 µM dye concentration (Supplementary Fig. 12). Gaussian mixture model and *k*-means clustering analyses were performed in the seven-dimensional space determined by the seven image parameters (Supplementary Fig. 13). The number of clusters was set to 3. For the Gaussian mixture fitting, the regularization parameter value was set to 1.

## Results

### The calcium-sensitive fluorescent dye readily enters the cell without signs of cell toxicity

**Fig. 1:**
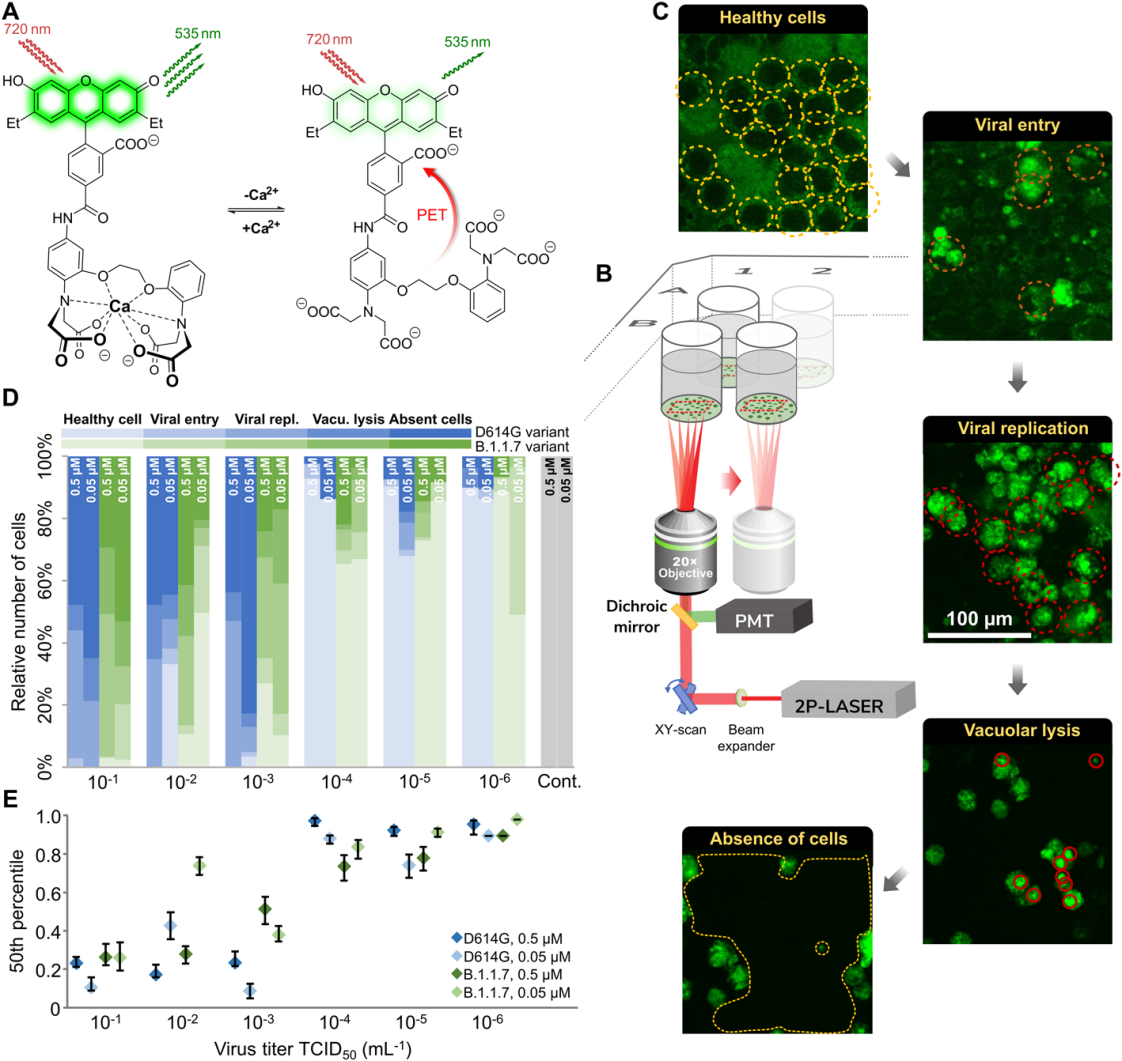
Experimental design and manual analysis of infection in Vero E6 cells using SARS-CoV-2 variants D614G and B.1.1.7. **A** Fluorescence enhancement of the BEEF-CP dye upon Ca^2+^ binding is attributed to the alleviation of the photoinduced electron transfer (PET) quenching. Its low background fluorescence and high two-photon cross-section (TPCS) along with its advantageous cell internalization make BEEF-CP an appropriate candidate for cytosolic Ca^2+^ imaging with 2P microscopy. **B** Experimental design with 2P microscopy. A pulsed Ti:Sa laser source was used for excitation at 700 nm wavelength, and laser light was diffracted with a pair of galvo mirrors for scanning the region of interest. A high-numerical-aperture objective (Olympus 20×) was used to obtain subcellular resolution. Samples were prepared in 96-well bioassay plates and transferred directly to the microscope. Samples were moved semi-automatically to locate the different wells in the field of view of the microscope. **C** Exemplary images from samples where most cells correspond to a particular stage of the infection (see “Methods” for more details on the characterization criteria). **D** The relative number of cells in different stages of infection 48 h post infection for the two variants at two different dye concentrations at various virus titers. Data from wells with the same virus titer, variant, and dye concentration are averaged. Statistical data for the individual cell categories are shown in Supplementary Fig. 5. **E** 50th percentile values, i.e., the percentage of cells under the median severity of infection, for the data displayed in **D**. Statistical analysis of the manual cell sorting shows that (i) the ratio of healthy cells is significantly higher with lower virus titer, regardless of the virus variant or dye concentration; and (ii) for high infection levels (TCID_50_ > 10^–3^ mL^−1^), the proportion of absent cells is higher for the D614G variant

BEEF-CP is an analog of the common Ca^2+^-sensitive dyes Calcium Green and Oregon Green that features nonpolar and electron-donating substituents, namely ethyl groups instead of halogen substituents on the fluorescein moiety (Fig. 1a). Fluorescein-based fluorophores are well known for their nonfluorescent lactone and fluorescent zwitterion tautomerism, which is heavily influenced by the substituents on the xanthene ring, pH, and other factors [26]. At the same time, the pH and Ca^2+^-dependent fluorescence of BAPTA-based Ca^2+^ indicators are a key factor in their applicability. At low or high pH values, these dyes can give false negative or false positive signals, respectively. The Ca^2+^ turn-on fluorescence experiments of BEEF-CP demonstrated low background and high enhancement in the biologically relevant pH range of 6.0–8.0 and consequently confirmed its suitability for in vitro measurements (Supplementary Fig. 21). The use of BEEF-CP in uninfected VeroE6 cells in a concentration of 0.5 µM was not associated with any cell toxicity signs measured over 24 h in the biosafety laboratory where these measurements were run along with cell infection experiments.

The spectroscopic characterization of BEEF-CP involved one-photon (1P) and 2P fluorescent measurements and the quantum yield (QY) determination in the presence and absence of Ca^2+^ ions. The measured fluorescence intensity at pH 7.2 showed a single-step enhancement upon Ca^2+^ addition in accordance with the formation of a single monometallic complex. The fluorescence enhancement was found to be around threefold in the biologically relevant Ca^2+^ concentration range, from 10 nM to 1 µM (Supplementary Fig. 20). The dissociation constant of the Ca^2+^ complex (*K*_D_ = 175.6 nM) is comparable with previously synthesized molecular probes for in vivo calcium level determination [34, 35]. The background fluorescence of BEEF-CP is not zero in the absence of Ca^2+^, which provides appropriate contrast to visualize even the healthy cells with a low level of Ca^2+^ ion during the cell viability assay. Furthermore, the high-slope region of the Ca^2+^-dependent fluorescent curve is adequately positioned to distinguish between healthy and malfunctioning cells.

The further applicability of BEEF-CP in cellular systems was tested by cytometry measurements on the HEK-293 cell line. Stained cells showed an increased mean fluorescence intensity by around one order of magnitude compared with the control level (Supplementary Fig. 17). In a parallel experiment, the stained HEK cells were treated with ethylene glycol-bis(β-aminoethyl ether)-*N*,*N*,*N*′,*N*′-tetraacetic acid (EGTA) prior to the analysis, which resulted in a somewhat lower mean fluorescence intensity. In contrast, cells stained in the presence of ionomycin using an otherwise identical procedure exhibit substantially higher mean fluorescence intensity. The ionomycin releases exclusively the intracellular Ca^2+^ storage, which proves that the dye is internalized by the cells as it responds to the cytosolic Ca^2+^ levels [36] (Table S5).

### Subcellular dye localization is characteristic and reproducibly different in uninfected and infected cell cultures

To perform high-resolution quantitative analysis of the level of SARS-CoV-2 infection, we used 2P imaging. This imaging technique has a high, subcellular spatial resolution (~ 350 nm) and virtually no out-of-focus signal from nonstudied cells or compartments, above or below of the imaging plane, which ensures that the detected signal originates only from the cells of interest. Samples were prepared on a 96-well bioassay plate with a clear, flat bottom for good optical access. One to three high-resolution, 2P, full-field, 360 × 360 µm large images were acquired with a Femto Smart 2P microscope at 700 nm wavelength (see Supplementary Fig. 3 for wavelength selection) from one or more representative area within each sample (see “Methods” for details).

**Fig. 2:**
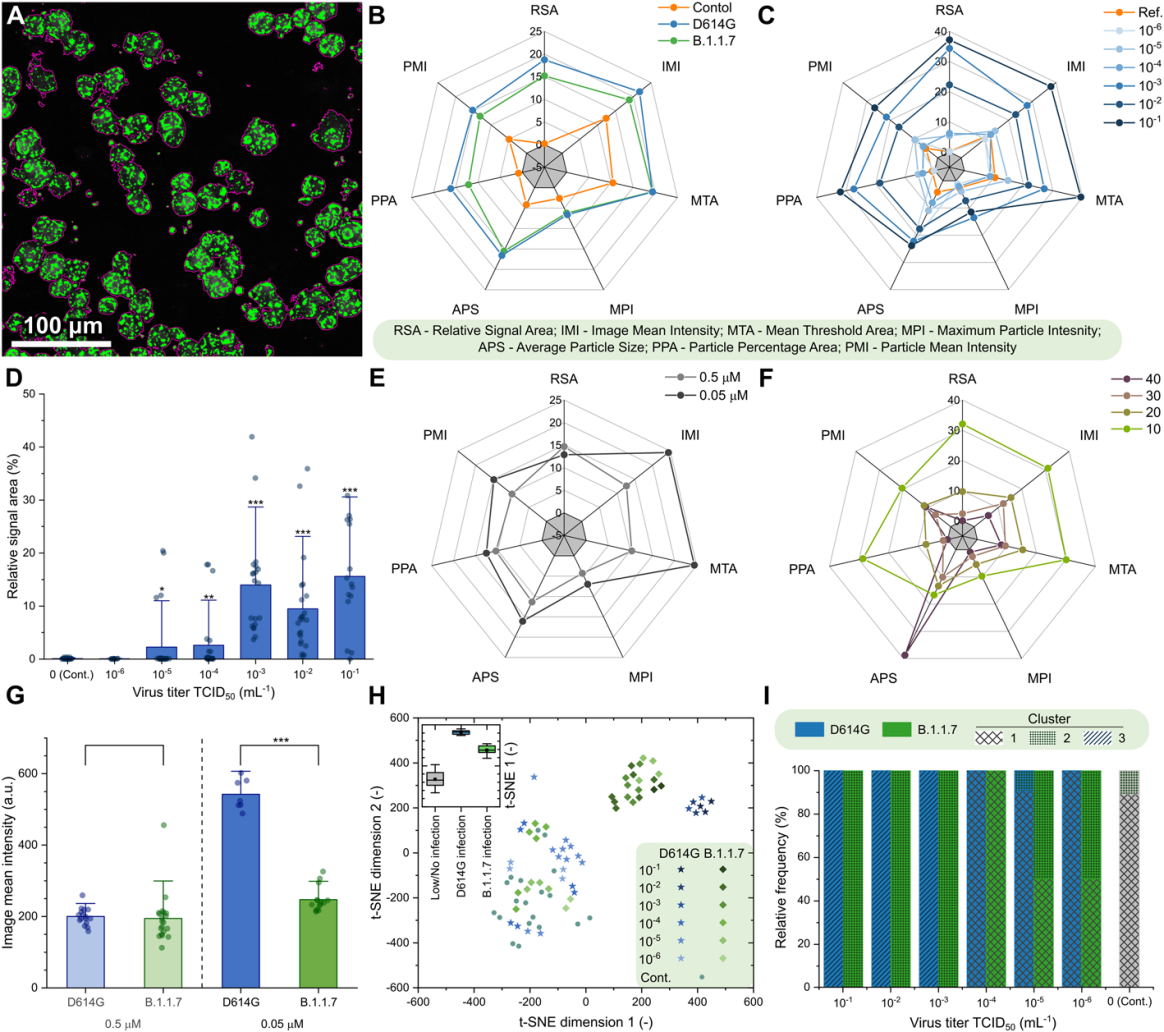
Automatic analysis of the 2P microscopy images of infected cells using SARS-CoV-2 variants D614G and B.1.1.7. **A** Purple contours indicate the areas automatically defined as particles during this analysis. **B**, **C**, **E**, **F** Spider charts showing the effects of **B** variant, **C** virus titer, **E** dye concentration, and **F** photomultiplier tube (PMT) relative voltage on the range-normalized values of seven different image parameters obtained from 2P microscopy images. The error bars for the spider charts can be found in Supplementary Fig. 7. **D** The image parameter called “relative signal area” shows a significant increase at virus titers higher than TCID_50_ 10^–3^ mL^−1^. **G** The image parameter called “image mean intensity” for the two studied variants at high virus titer (TCID_50_ > 10^–3^ mL^−1^) at 0.05 μM and 0.5 μM dye concentration. **H** *t*-Distributed stochastic neighbor embedding (t-SNE) 2D plot obtained from all the seven image parameters recorded at 0.5 µM dye concentration shows three clusters depending on the virus variant and titer. Inset shows that the three groups, namely no or low infection, infection with D614G variant, and infection with B.1.1.7 variant are clearly separated along the first dimension. **I** Classification of the images corresponding to different variants at various virus titer in three clusters by seven-dimensional Gaussian mixture model clustering (error bars show standard deviation; significance levels as *p ≤ 0.1; **p ≤ 0.05; ***p ≤ 0.01.)

Acquired images were analyzed with two complementary methods (manual and automatic). During manual evaluation, the cells were sorted according to their morphology (Fig. 1b–d), while the automatic analysis, based on statistical methods, consisted of the classification of the full images based on a set of image parameters (Fig. 2). Both methods take the different features of the acquired raw images into account for the analysis, and for both we investigated the fine details of the images (at cellular or single-pixel level) for designing a quantitative measure correlating with the initial challenge infection level. Two variants of concern of SARS-CoV-2, namely D614G and B.1.1.7, were studied (Supplementary Fig. 6). These caused pathophysiologically and clinically distinguishable disease patterns in the clinic, so the putative infection quantitation and infection assay properties of BEEF-CP would be tested as the distinguishing features of cellular Ca^2+^-related effects of these two variants. Different cellular features such as the state of infection and the apoptotic-like state were visible and distinguishable on the images of Vero E6 cells infected with the two SARS-CoV-2 variants (Fig. 1b). The investigations were done in a phase when still living cells (both healthy and infected) adhered to the plate surface. Cell compartments, such as cell membrane and nucleus, were visible and distinguishable in the samples (Supplementary Fig. 15). With increasing virus titer, the mean fluorescence of infected cells increased significantly as this caused more Ca^2+^ to enter the cytoplasm. When the infecting viral titer increased even further, the morphology of cells changed, and dead cells detached from the wells plate; thus, the number of these absent cells could be estimated by the ratio of unpopulated areas at the plates’ surface. Due to the subcellular resolution of this cell viability assay, we could detect the formation of several syncytial formations, as giant multinucleated cells in the most infected cultures, which agrees with the earlier observation that SARS-CoV-2 virus causes this type of cellular fusion (Supplementary Fig. 2) [37, 38].

### Manual cell counting quantifies infection progress

During the manual evaluation, cells were manually localized on the recorded images, and then the individual cells were visually categorized into five groups, representing different stages of the infection (see Fig. 1c and “Methods” for more details on cell categories). The individual images from different wells were subjected to a single-blind evaluation by a human operator. The cells were classified into four categories (Fig. 1c), while the fifth category, cell death, was determined by the absence of living cells, estimated from the surface area that was not covered with cells, as shown in Fig. 1c. The number of dead cells is not directly counted; instead, it was estimated from the number of missing or absence of cells relative to an average number of cells from images without infections. However, one may observe some variability in the number of overall cells per field of view, provided in Supplementary Tables 1 and 2. The ratio of the cells belonging to each category was calculated for the different virus variants, virus titer, and dye concentration showing an increasing number of cells in the healthy or initial infection categories with the virus titer decreasing (Fig. 1d and Supplementary Tables 1 and 2). The 50th percentile values were calculated for each virus titer by averaging the result from all wells infected with the given titer. For this calculation, cells were sorted by the severity of their infection from healthy to absent dead cells, and the position of the median was calculated as the ratio of all the cells. The 50th percentile values also show a clear trend with increasing viral load. Furthermore, there is a clear jump in the severity of the infection in the cell culture between TCID_50_ virus titer 10^–4^ and 10^–3^ mL^−1^.

### Automatic image analysis identifies advanced infection and virus variant

An ImageJ [39] macro was created to identify particle objects based on an intensity threshold (Fig. 2a) and to extract image data from the fluorescence microscopy images in the form of a few numerical parameters. The ImageJ macro is available in Appendix A. Seven parameters characterizing the detected particles were defined in each image: (i) relative signal area, (ii) image mean intensity, (iii) mean of the threshold area, (iv) maximum particle intensity, (v) average particle size, (vi) particle percentage area, and (vii) particle mean intensity (Supplementary Table 3).

The input images correspond to experimental points that can be described by two infection-related variables, namely virus titer and virus variant, and by two measurement-related variables, namely BEEF-CP sensor concentration and photomultiplier tube (PMT) voltage. Analysis of the image parameters as a function of the infection-related and measurement-related variables can yield information about the efficiency and robustness of the automatic image analysis, respectively. Figure 2b–c, e–f shows that the image parameters vary according to different acquisition settings, i.e., virus concentration, virus type, dye concentration, and PMT voltage. Importantly, certain image parameters such as “relative signal area” (Fig. 2d) can be a good indicator of the viral infection as they show increasing parameter values with increasing virus titer. However, images of the two variants did not show clear separation by any parameter values without eliminating the variation caused by the measurement-related variables.

Other prevalent Ca^2+^ sensors, such as OGB-1 AM (Oregon Green 488 BAPTA-AM), are used at a high concentration, around 1 mM (ranging from 0.9 to 1.4 mM) [40–42]. We used BEEF-CP at lower concentrations, at 0.5 µM and 0.05 µM. Both concentrations were suitable to differentiate between little or no infection and a high level of infection using automatic image analysis. Both these concentrations, however, are above the typical toxicity level, influencing the results. Moreover, differentiation of the variants responsible for the infection became possible when the images obtained with higher dye concentration (0.5 µM) were analyzed in those cases when the well was highly infected (TCID_50_ > 10^–3^ mL^1^) (Fig. 2g). Therefore, for further analysis, only those images were considered that were acquired from samples stained with 0.5 µM dye concentration.

A cluster analysis that considers all seven parameters was run on all the images acquired using 0.5 µM dye concentration. Two-dimensional visualization of the seven-dimensional clustering (Fig. 2h) shows three separated clusters (see Experimental procedures and Supplementary Figs. 7–14 for the detailed analysis). A three-component Gaussian mixture model cluster analysis was performed on the image parameters. Figure 2i shows that the three obtained clusters agree well with the three distinct states of the wells, i.e., little or no infection, highly infected with the D614G virus variant, and highly infected with the B.1.1.7 variant.

### Quantitative image analysis shows a viral variant-dependent separation and a clear infection threshold of the applied virus titers

The manual and automatic analyses both showed a difference between the two tested virus variants, with D614G having a significantly higher infection rate. This finding complements earlier results that found a higher infection rate for D614G than B.1.1.7 in HeLa ACE2 and similar rates in HEK-293 T cells [43]. There is a very clear threshold found over 10^–3^ mL^−1^ TCID_50_ virus titer, where instead of occasional virus infection spread, we saw reliably escalated infection at all imaging sites. Furthermore, the automatic analysis revealed other important findings. First, it showed a clear threshold for the dye titer, showing that at least 0.5 µM dye concentration is required for the significant differentiation between variants (Fig. 2g). The results suggest that the fluorescence intensity of infected cells—related to the image mean intensity—is delimited by the dye concentration at 0.05 µM and by the Ca^2+^ concentration at 0.5 µM dye load. Second, the cluster analysis of the data could distinguish three separate groups (Supplementary Fig. 12). Among these was the noninfected group; the images falling into this cluster showed similar characteristics to the control measurements. However, more interestingly, the remaining images were almost perfectly separated by the virus variant used for the infection (Fig. 2h). These results demonstrate that this method is capable of quantifying the infection level and can also differentiate the two applied variants, D614G and B.1.1.7.

## Discussion

This study presents a general concept of quantitative calcium-based viral cellular harm assay, here applied on SARS-CoV-2 viral variants. For quantitative analysis of SARS-CoV-2 infection, we chose to monitor the Ca^2+^-dependent cellular mechanism previously described in the literature as it strongly correlates to the infection rate. For monitoring the intrinsic concentration of Ca^2+^, a novel cell-membrane-permeable internalized, two-photon active Ca^2+^ selective sensor molecule was designed and synthesized, which allows the measurement of both extra- and intracellular Ca^2+^ concentration, without chemical compromises. BEEF-CP as a Ca^2+^-selective sensor exhibits several advantages for practical applicability, such as (i) appropriate cell internalization [44]; (ii) the effective concentration of the dye is 2–20 times lower (0.5–0.05 µM) compared with other widely used compounds (> 1 µM); and (iii) it is free of toxic leaving groups and, hence, infection processes are not affected by the chelation of Ca^2+^ using the novel dye, unlike in the case of working concentrations for BAPTA-AM; and (iv) appropriate spectrophysical characteristics and sufficient pH insensitivity around the physiological pH.

As we used the fluorescence intensity profile of the images acquired for quantitative analysis, it was imperative to measure only a single cellular layer without contamination from the out-of-focus cells or compartments. This was achieved by means of 2P imaging. Furthermore, it should be noted that the proposed imaging method can be directly applied to tissue slices or organoids, opening new ways to test the effects of SARS-CoV-2 infections in more complex systems as well. The high-resolution detection of the Ca^2+^ level within the infected cells requires a sensor molecule with appropriate 2P sensitivity and adequate cell internalization. The developed sensor, BEEF-CP meets these criteria owing to its diethylfluorescein fluorophore. The fluorescent dye concentration influences the information content of the images; 0.5 µM—unlike 0.05 µM—proved to be sufficient to determine not only the level of infection but also the virus variant. Importantly, neither applied dye concentration induced cell death in the control wells. The incubation time (60 min) was sufficiently short for rapid imaging of the fast dynamics of the infection.

Two different methods, with comparable results, have been used to evaluate imaging data. The first method allowed, by manual cell counting, to quantify the number of cells in different stages of the infection such as (i) healthy cells, (ii) initial infection of the cells, (iii) infected cells, (iv) infected vacuoles, and (v) dead cells (as the absence of cells). The second method automatically extracted image parameters from the image files to compare the different infection states without needing manual categorization. With this methodology that works in BSL3 environment, we demonstrated that the increase of the intracellular Ca^2+^ ion concentration is a good indicator of the infection progress as the concentration of the calcium changes significantly inside the cell during virus infection. Correlates of infection in relationship to intracellular and fusogenicity-related calcium concentration changes might offer a prospective and reliable way of infection and therapy monitoring in vitro.

## Conclusions

We propose a new calcium-sensitive dye for correlates of SARS-CoV-2 infection determination, quantification, and monitoring in two- and three-dimensional cell cultures using 2P microscopy. This molecule is readily and easily applicable in biosafety and repeated cell culture monitoring conditions. The concentrations applied in our study suggest that the molecule performs comparably to previously applied calcium dyes in other applications. Our BSL-3 2P microscopy measurements have also established that the applied concentrations of the dye do not interfere with viral replication and viral fusion events. We postulate that this method thus ensures proper monitoring of the full viral entry spectrum of events. At the same time, it also enabled us to distinguish intracellular details of cell damage, such as vacuole and apoptotic body formation. Using clustering analysis, we could use the 2P microscopy calcium fluorescence images for the distinction of two different viral variants in cell cultures. Using this 2P microscopy method, we could also establish correlates of infection related to the initial viral multiplicity of infection numbers. Further research may utilize this technique to study and compare the effects of antiviral drug candidates on the infection in cell cultures.

### Supplementary Information

Supplementary Material 1.

## Data Availability

The authors declare that all the data supporting the findings of this study are available within the paper and the Supplementary Data. Raw two-photon microscopy images can be found in Supplementary Dataset 1.^29^ Additional raw data are available from the corresponding authors upon request.

## Abbreviations

1P: One-photon
2P: Two-photon
AM: Acetoxymethyl
BAPTA: 1,2-*bis*(*O*-Aminophenoxy)ethane-*N*,*N*,*N*′,*N*′-tetraacetic acid
BEEF-CP: BAPTA-enabled diethyl-fluorescein-calcium probe
BSL: Biosafety level
GFP: Green fluorescent protein
EGTA: Ethylene glycol-*bis*(β-aminoethyl ether)-*N*,*N*,*N*′,*N*′-tetraacetic acid
OGB-AM: Oregon Green 488 BAPTA-AM
PET: Photoinduced electron transfer
PMT: Photomultiplier tube
TCID_50_: Tissue culture infectious dose
TPCS: Two-photon cross-section
t-SNE: *t*-Distributed stochastic neighbor embedding
