# Supplementary material for: Monitoring correlates of SARS-CoV-2 infection in cell culture using a two-photon-active calcium-sensitive dye
Source: Cell Mol Biol Lett. 2024 Jul 19;29:105. doi: 10.1186/s11658-024-00619-0 (PMC11264913; doi:10.1186/s11658-024-00619-0)
Supplement: Supplementary file 1 — Supplementary Material 1. [file 11658_2024_619_MOESM1_ESM.pdf]

Supplementary Information

for

Monitoring correlates of SARS-CoV-2 infection in cell culture using a two-photon-active calcium-sensitive dye

Domokos Máthé[a,b,c,†,*], Gergely Szalay[d,e,†], Levente Cseri[e,f,†], Zoltán Kis[g], Bernadett Pályi[g], Gábor Földes[h,i], Noémi Kovács[b], Anna Fülöp[f], Áron Szepesi[d,e], Polett Hajdrik[a], Attila Csomos[f,j], Ákos Zsembery[k], Kristóf Kádár[k], Gergely Katona[l], Zoltán Mucsi[e,f,m,*], Balázs József Rózsa[d,e,l,*], Ervin Kovács[l,n,*]

[a]Department of Biophysics and Radiation Biology, Semmelweis University, Tűzoltó utca 37–47, H-1094 Budapest, Hungary
[b]In Vivo Imaging Advanced Core Facility, Hungarian Centre of Excellence for Molecular Medicine, Tűzoltó utca 37–47, H-1094 Budapest, Hungary
[c]HUN-REN Physical Virology Research Group, Semmelweis University, Tűzoltó utca 37–47, H-1094 Budapest, Hungary
[d]Laboratory of 3D Functional Network and Dendritic Imaging, HUN-REN Institute of Experimental Medicine, Szigony utca 43, H-1083 Budapest, Hungary
[e]BrainVisionCenter, Liliom utca 43–45, H-1094 Budapest, Hungary
[f]Femtonics Ltd., Tűzoltó utca 59, H-1094 Budapest, Hungary
[g]National Center for Public Health, Albert Flórián út 2–6, H-1097 Budapest, Hungary
[h]National Heart and Lung Institute, Imperial College London, Du Cane Road, W12 0NN London, United Kingdom
[i]Heart and Vascular Center, Semmelweis University, Városmajor utca 68, H-1122 Budapest, Hungary
[j]Hevesy György PhD School of Chemistry, Eötvös Loránd University, Pázmány Péter sétány 1/A, H-1117 Budapest, Hungary
[k]Department of Oral Biology, Faculty of Dentistry, Semmelweis University, Nagyvárad tér 4, H-1089 Budapest, Hungary
[l]Two-photon measurement technology group, The Faculty of Information Technology, Pázmány Péter Catholic University, Szigony utca 50/A, H-1083 Budapest, Hungary
[m]Institute of Chemistry, Faculty of Materials Science and Engineering, University of Miskolc, Egyetem tér 1, H-3515 Miskolc, Hungary
[n]Institute of Materials and Environmental Chemistry, HUN-REN Research Centre for Natural Sciences, Magyar tudósok körútja 2, H-1117 Budapest, Hungary

[†]These authors contributed equally to this work.

*For correspondence: mathe.domokos@med.semmelweis-univ.hu (MD); zmucsi@femtonics.eu (ZM); rozsa.balazs@koki.hu (BJR); kovacs.ervin@ttk.hu (EK)

Contents

Supplementary methods 3

Viral isolate and viral infection gradient plate 3

Flow cytometry methods 3

Supplementary figures and tables 5

ImageJ script 27

Microbiological assay results 29

Cytotoxicity assay 29

Flow cytometry measurement results 30

Synthetic methods and results 31

General synthetic methodologies and characterization 31

Synthesis of BEEF-CP 32

Synthesis of ketone 5 32

Synthesis of unprotected fluorescein derivative 6 33

Acylation of the fluoresceine derivative 6 33

N-acylation of amino BAPTA moiety using 3 34

Hydrolysis of 7 using aqueous sodium hydroxide 35

Spectroscopic characterization of BEEF-CP 36

Single-photon absorption and emission of BEEF-CP 36

Determination of dissociation constant (K_d) of fluorescent calcium indicator BEEF-CP 38

pH sensitivity of the fluorescent sensor BEEF-CP 39

Ion-selectivity of the fluorescent sensor BEEF-CP 40

Two-photon action cross-section measurements 41

NMR spectra of the synthesized novel compounds 42

Supplementary data references 52

Supplementary methods

Viral isolate and viral infection gradient plate

D614G mutant variant of the ancestral Wuhan-Hu-1 SARS-CoV-2 virus, isolated from a Hungarian patient in 4 July 2020. SARS-CoV-2 B1.1.7 (Alpha) variant isolated from a Hungarian patient in October 2020. All experiments with the application of infective material were performed under Biosafety Level 3 (BSL-3) conditions at the National Biosafety Laboratory, National Public Health Center (Budapest, Hungary). One day prior to infection Vero E6 cells were plated in 96-well flat bottom tissue culture plates (TPP, Switzerland) in vaccine production-serum free medium (VP-SFM) until each well reached 80% confluence.

The Vero E6 cells for isolation were maintained in DMEM (Lonza, Switzerland) medium supplemented with 5% OptiClone fetal bovine serum (FBS, Euroclone, Italy) and Cell Culture Guard (PanReacApplichem, Germany). The viral titers were determined by the median tissue culture infectious dose assay (TCID50) method and calculated using the public domain code TCID50 calculator v2.1 [Binder M. TCID50 Calculator (v2.1-20-01-2017_MB) [(accessed on 10 June 2020)]; available online: https://www.klinikum.uni-heidelberg.de/fileadmin/inst_hygiene/molekulare_virologie/Downloads/TCID50_calculator_v2_17-01-20_MB.xlsx].

The basic viral titer of the stock solution was calculated as 1×10^{-1} ml^{-1}. Subsequently, a tenfold serial dilution was performed using the stock solution, i.e. from 10^{-2} until 10^{-6} $TCID_{50}$, each in a final volume of 100 microliters. This volume was supplemented to a final volume of 200 microliters with VP-SFM and $TCID_{50}$ added to the plate wells for the viral infection calcium imaging tests in triplicate. A positive control triplicate using 10^{-1} $TCID_{50}$ and a negative uninfected control triplicate were also plated.

The plates were incubated in a humidified 37 °C incubator at 5% CO_2 for forty-eight hours to allow for the cytopathogenic effects of viral replication to complete.

For imaging, the fluorescent dye solution was added to each well in 10 microliters of volume in a solution of 10 or 100 times diluted original stock (1 mg, 10^{-6} mol of dye dissolved in 1 mL of ethanol, which was diluted with 9 mL of distilled water resulted in the dye in 100 μM concentration in original stock, which was diluted to 10 μM or 1 μM before added to the wells). The dye concentration in the wells were 0.5 or 0.05 μM, respectively. The supernatant was replaced after 60 minutes of incubation prior to the imaging.

Flow cytometry methods

The flow cytometry measurement was carried out on a BD-FACSVerse instrument (BD company) including three laser sources and 8 channel in FICS channel. The HEK-293 cell line was implemented according to the generic vendor protocol at 37 °C in a humidified atmosphere with 5% CO_2. The base medium for this cell line is D-MEM with 4.5 g L^1 glucose. To make the complete growth medium, fetal bovine serum was added to a final concentration of 10%.

For calcium efflux measurement we used a cell suspension with 1×10^6 cells in 1 mL HBSS (Thermo Fisher) solution. Cells were loaded with BEEF-CP dye (0.01 mL, 100 μM) or without dye at HBSS as untreated control. Cells were incubated for 45 minutes at 37 °C in a shaking incubator (500 rpm) in the dark. Cells were washed twice with DMEM with 2% FBS and resuspended in HBSS. For recovering, the cells were stored in the dark at

room temperature until about 0.5–1 hour. The samples were measured with a 3 laser, 8 channel BD FACSVerse instrument.

The baseline fluorescence was determined with untreated cells. For maximal calcium flux ratio, cells were treated with 1 µg/ml ionomycin (Thermo Fisher) for 5 minutes and measured. For negative control cells were inhibited with EGTA. Positive control: Ionomycin, 1 µg mL^{-1} final concentration. Negative control: EGTA 8 mM final concentration. The data were analyzed with BD FACSuite Software.

Supplementary figures and tables

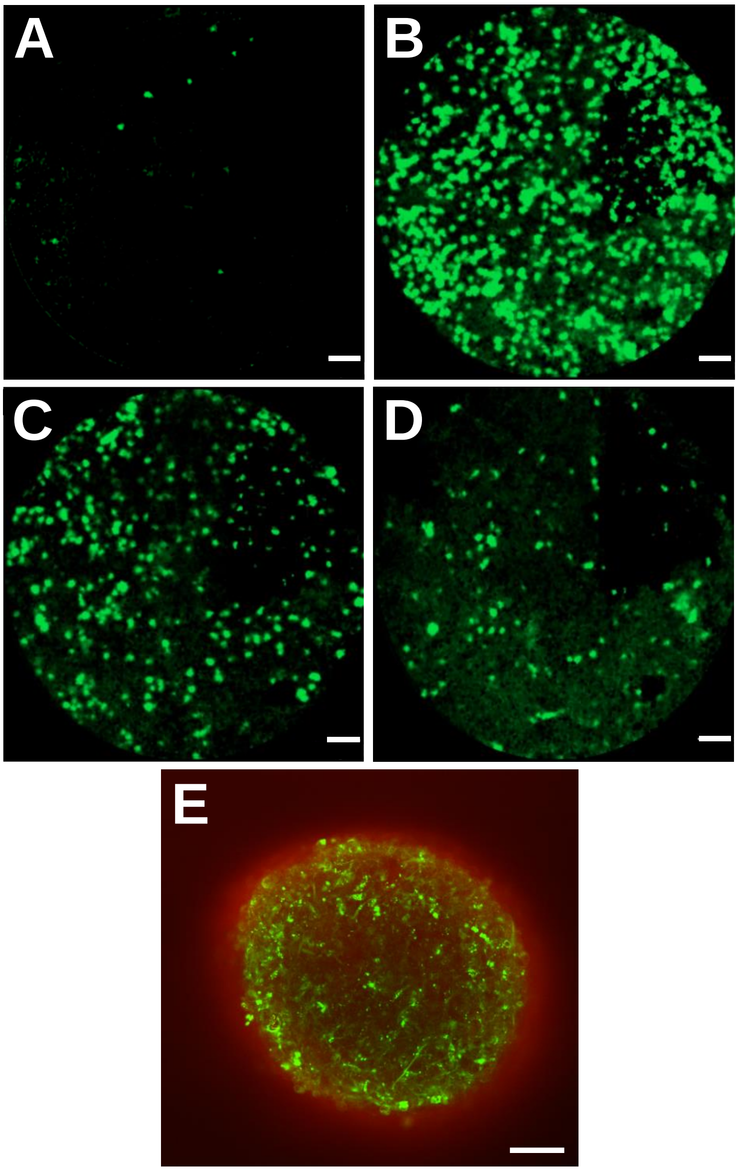

Fig. S1. Images acquired with a Cellvizio confocal laser scanning fiber optic endomicroscope (CellVizio Dual Band, Mauna Kea Tech Srl, Paris, France; fiberoptic dual wavelength confocal endomicroscopic system. The applied optode was the S-800 with a 800 micron field-of-view and 3 micron resolution) using the 490 nm excitation. Panels (A) and (B) show the cellular calcium response in bright green fluorescence using the novel calcium sensing dye BEEF-CP (0.05 µM), while (C) and (D) show the use of the dye in normal non-infected cell culture. Besides the obvious viral titer dependency of the readout calcium light response, the depth dependent changes in resolution and cellular image blurring are also emphasized when this non-3D method of imaging is applied in real-life 3D cell culture conditions. (E) Two-photon (green) and one-photon (red) images (separated by the stimulation wavelength) from the same organoid sample overlayed. One-photon signal, acquired through the two-photon detectors (with no pinhole). Scale bars (on all panels) 50 µm.

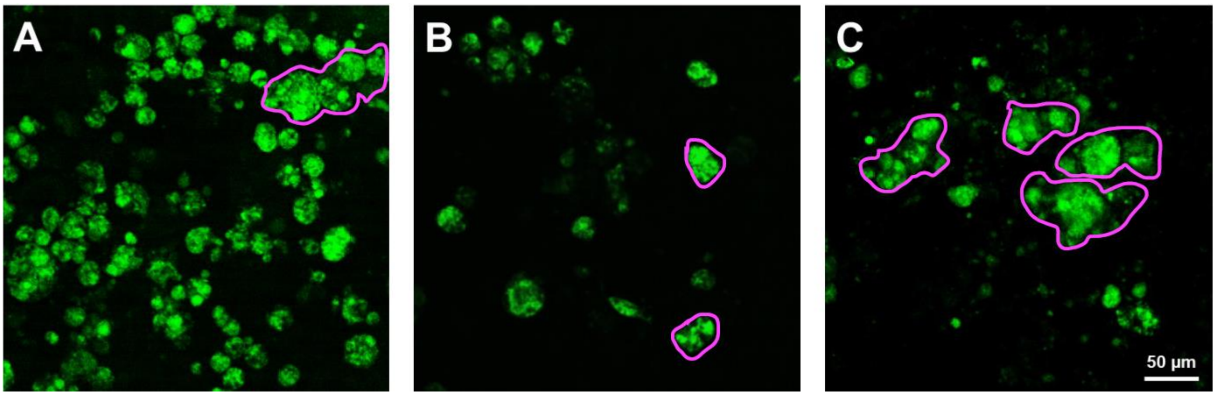

Fig. S2. Syncytium formation. (A-C) Examples for syncytium formation with different syncytium sizes, assumably with increasing number of contributing cells. Exemplified syncytial formations are outlined in pink.

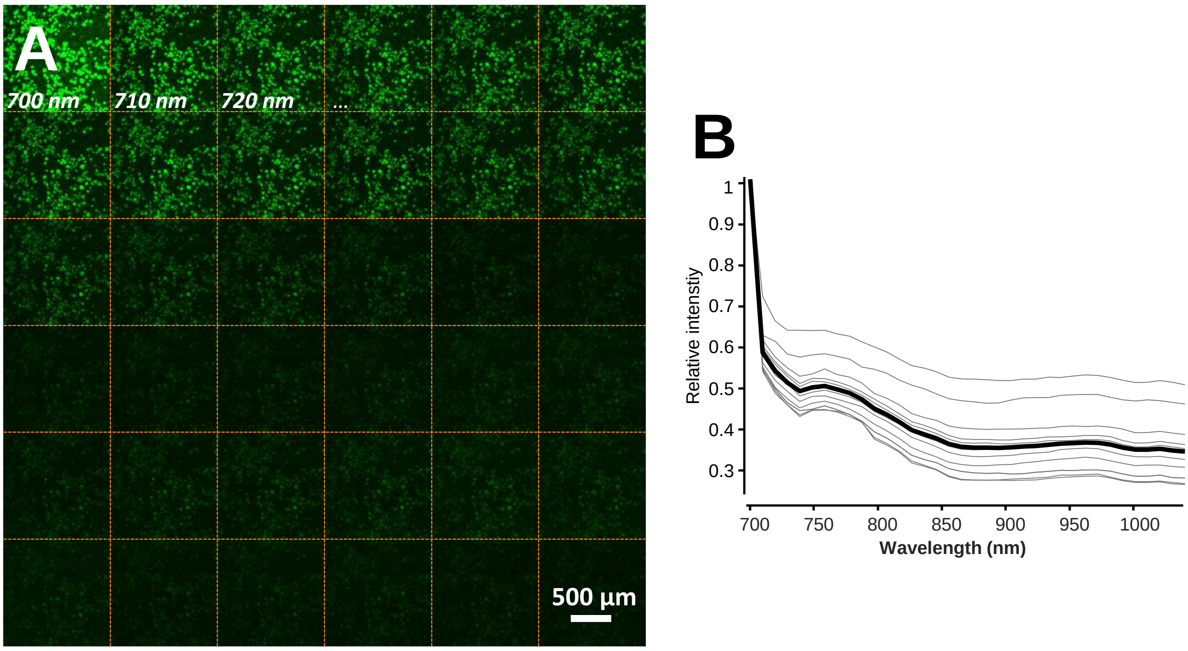

Fig. S3. Wavelength selection. (A) 'Wavelength stack', images acquired from the same imaging region without changing the laser intensity or PMTs' setting. (B) Averaged two-photon fluorescent signal as a factor of the wavelength, as in (A). Data was pulled from hand selected image areas contains distinguishable structures. Gray lines indicate individual measurements, from different well-plates, intensity is normalized to the one measured at 700 nm. Black line indicates average of 10 measurements with a clear stimulation peak at 700 nm. This 700 nm excitation wavelength was used for all further measurements.

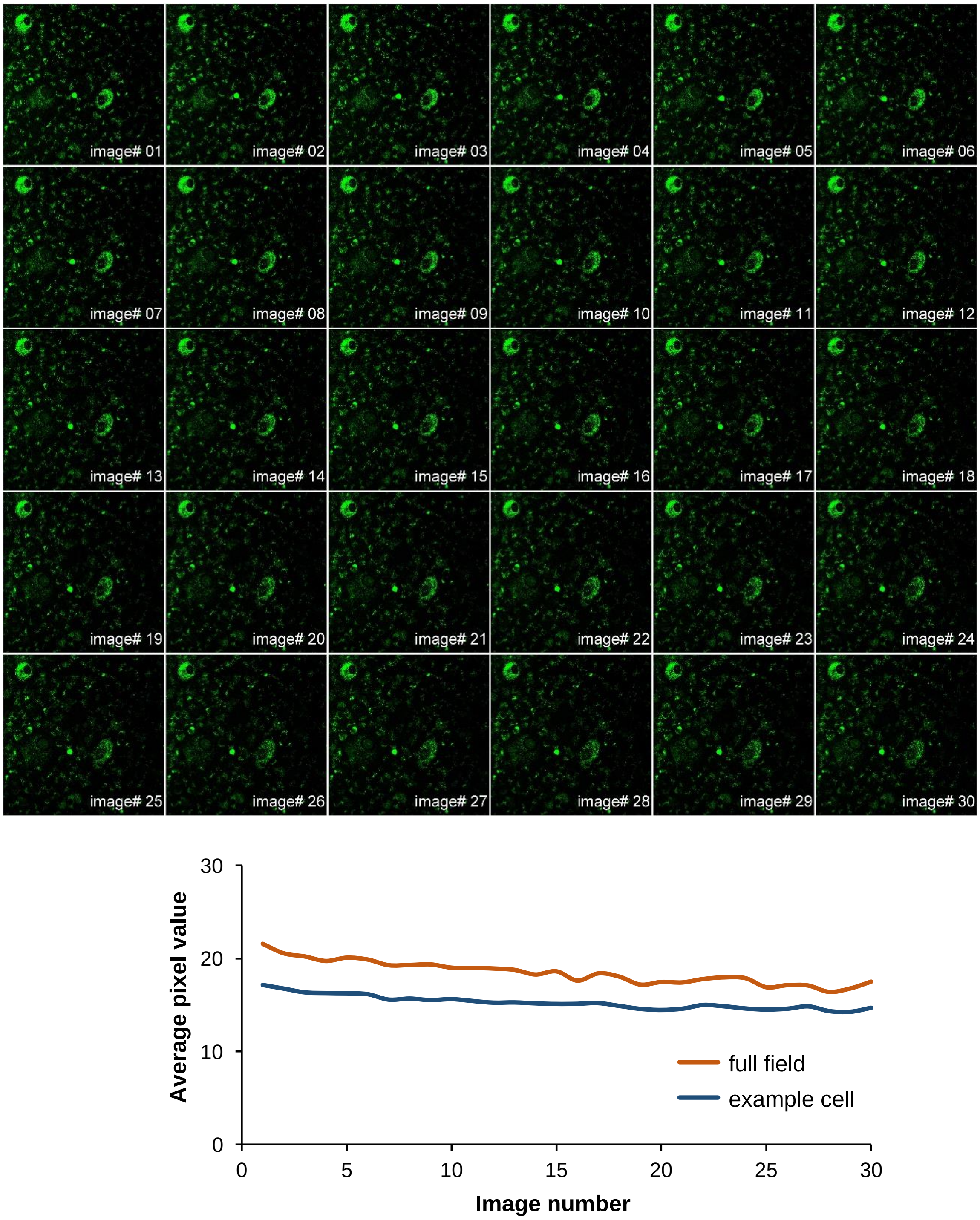

Fig. S4. Effect of long-term imaging. (Top panel) 30 consecutive images acquired from the same region with 30s difference, with no apparent change in the overall morphology of the cells or the culture. (Lower panel) Decrease of the average fluorescence intensity for the full field of view and for one example cell.

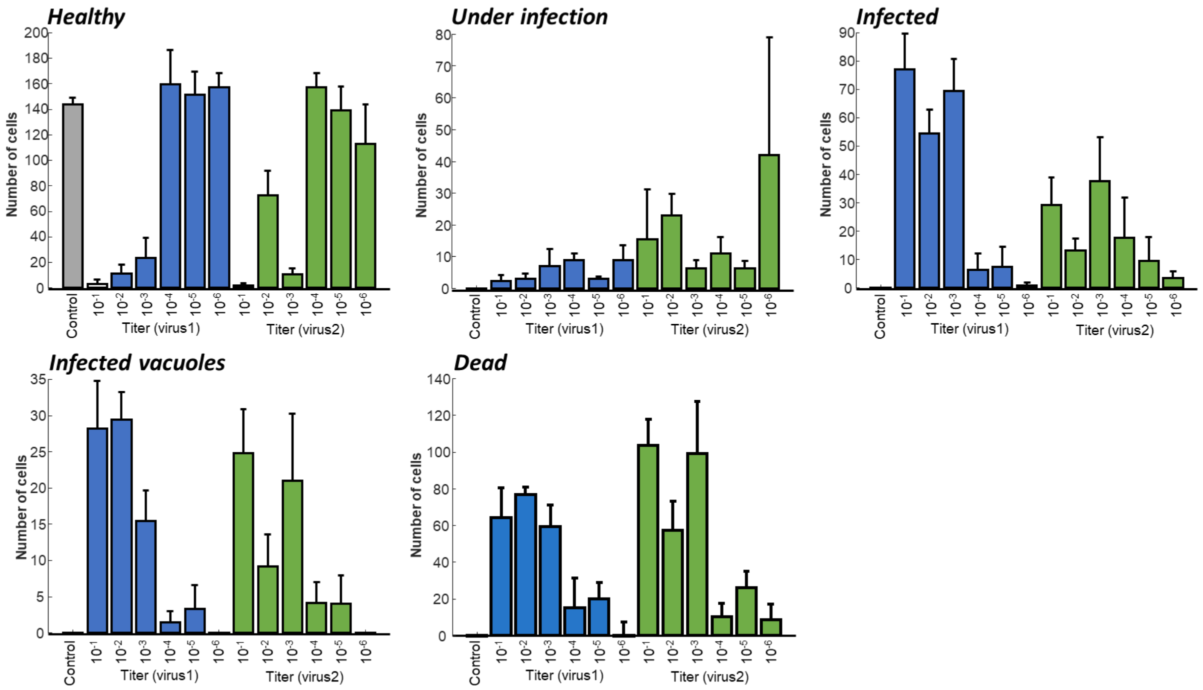

Fig. S5. Distribution of cells in different infection state as the function of the virus titer. All graphs show the average number of cells found in the different cell categories per image. If multiple images were taken with the same condition the data is averaged. Error bars show ± S.E.M.

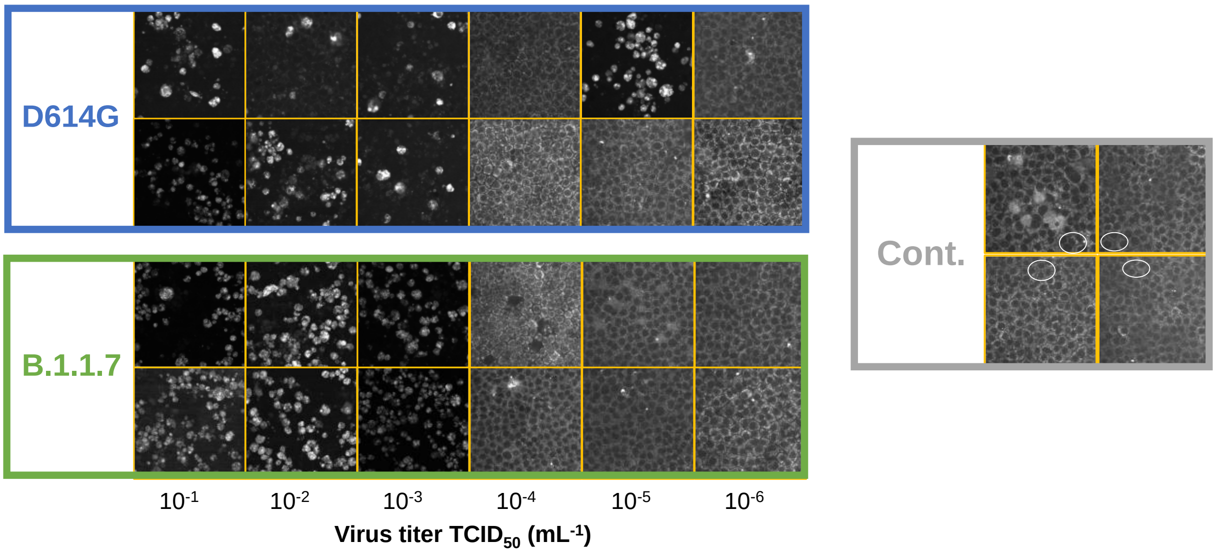

Fig. S6. Exemplary images of the different levels of infection. Representative images about how the applied virus titer influences the appearance of the labeled cell culture during two-photon imaging. Blue square shows the samples labeled with the D614G variant, green square for the B.1.1.7 variant. Gray (Cont.) panel square shows the Control images. Healthy cell membranes are exemplified with a white circle in Cont. panel.

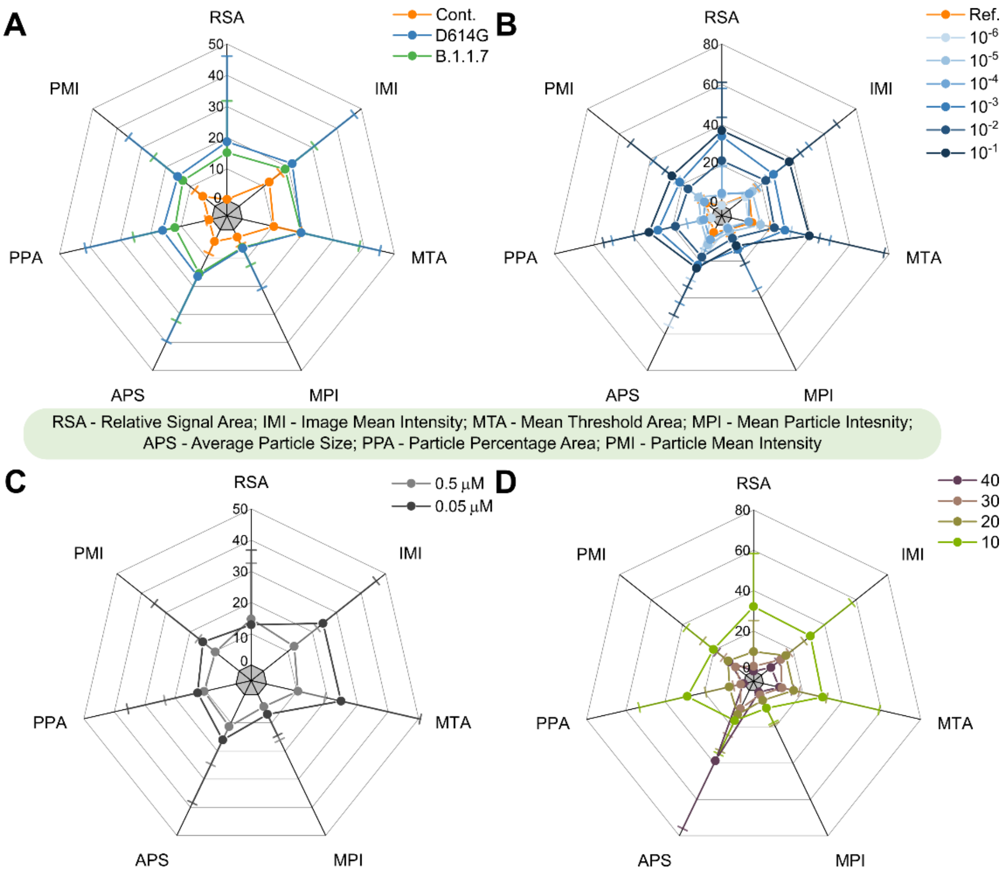

Fig. S7. Spider charts showing the effects of (A) variant, (B) virus titer, (C) dye concentration, and (D) photomultiplier tube (PMT) relative voltage on the range-normalized values of 7 different image parameters obtained from 2P microscopy images. This figute shows the same data as Fig. 2(B,C,E,F) but with error bars included.

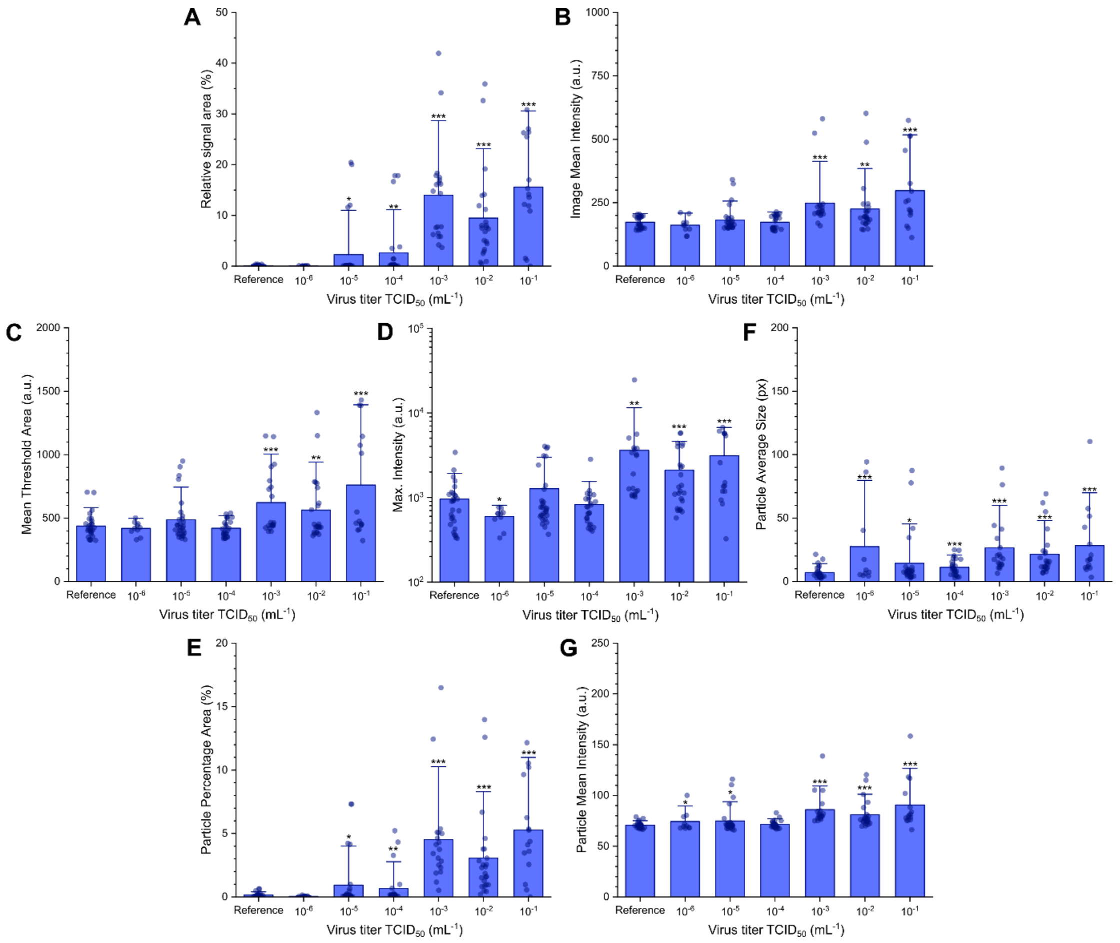

Fig. S8. Image parameters from the automatic image analysis plotted against virus titer ($TCID_{50}$). The automatic analysis determined seven different parameters for each image. Dots represent the measurements from the individual images, while blue bars show the averages for the given measurement conditions. (Error bars show standard deviation; significance levels as: * = $p \leq 0.1$; ** = $p \leq 0.05$; *** = $p \leq 0.01$).

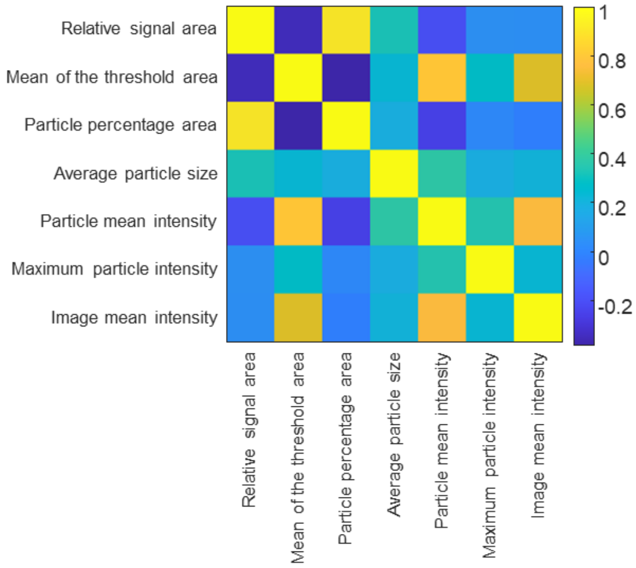

Fig. S9. Cross-correlation between the parameters. Cross-correlation between all the seven parameters. None of the correlation coefficient besides the center axes was higher than 0.8, therefore all parameters could be used during the cluster analysis.

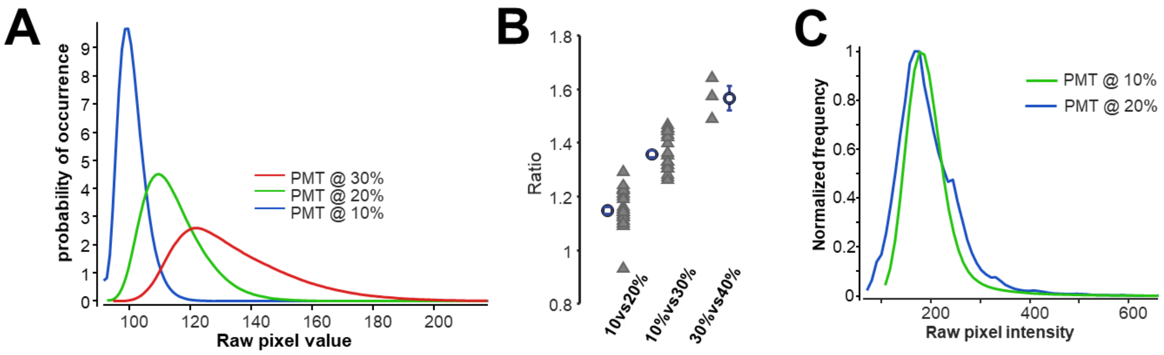

Fig. S10. Normalization for different PMT voltages. (A) Exemplified pixel intensity histograms for an imaging region that has been measured with three different PMT voltage. For comparison between images measured at different PMT voltage, images at 20% and 30% are normalized to the level of the images which were measured. For this normalization an offset and a division have been used. The offset and division coefficients were determined so that the curves have similar mean and deviation. Coefficient from multiple image duplets and triplets were averaged. This average was used for all the images. **(B)** Division ratios for the different PMT voltage differences. Triangles show the individual calculations while circles show the averages. Error bars represented as S.E.M. **(C)** Show of an example of the normalized histogram. Curvfes were drawn from the images acquired with virus titer of 10^{-2} mL^{-1} $TCID_{50}$ for the B.1.1.7 variant. For 10% PMT case data is pulled from the original figure while the image acquires at 20% PMT was normalized as above before the histogram is calculated.

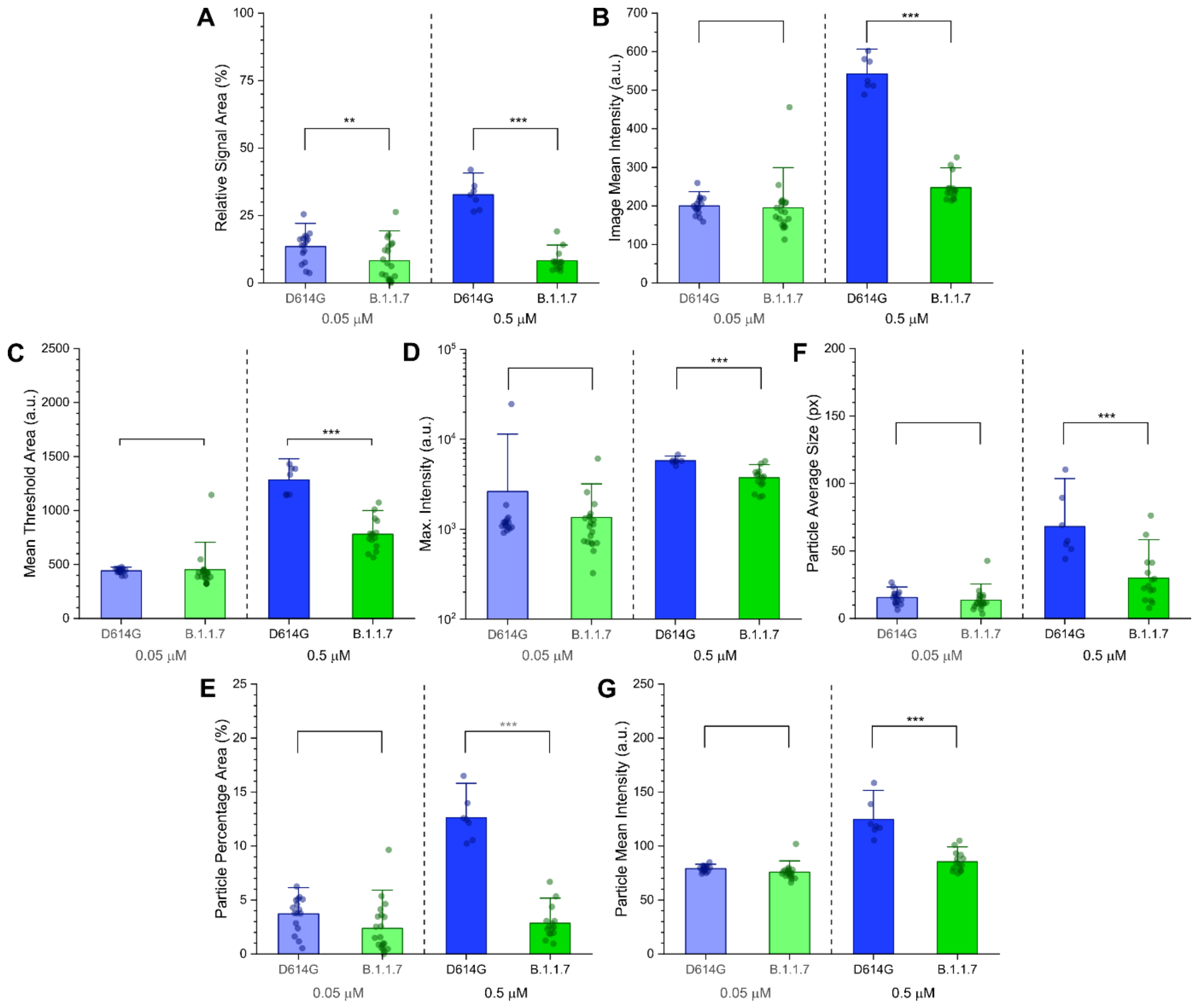

Fig. S11. Image parameters from the automatic image analysis plotted against the virus variant at different dye concentrations (i.e. 0.5 µM and 0.05 µM). Only data points corresponding to virus titer of 10^{-3} mL^{-1} $TCID_{50}$ and higher were considered. Dots represent the measurements from the individual images, while bars show the averages for the given measurement conditions. (Error bars show standard deviation; significance levels as: * = $p \leq 0.1$; ** = $p \leq 0.05$; *** = $p \leq 0.01$).

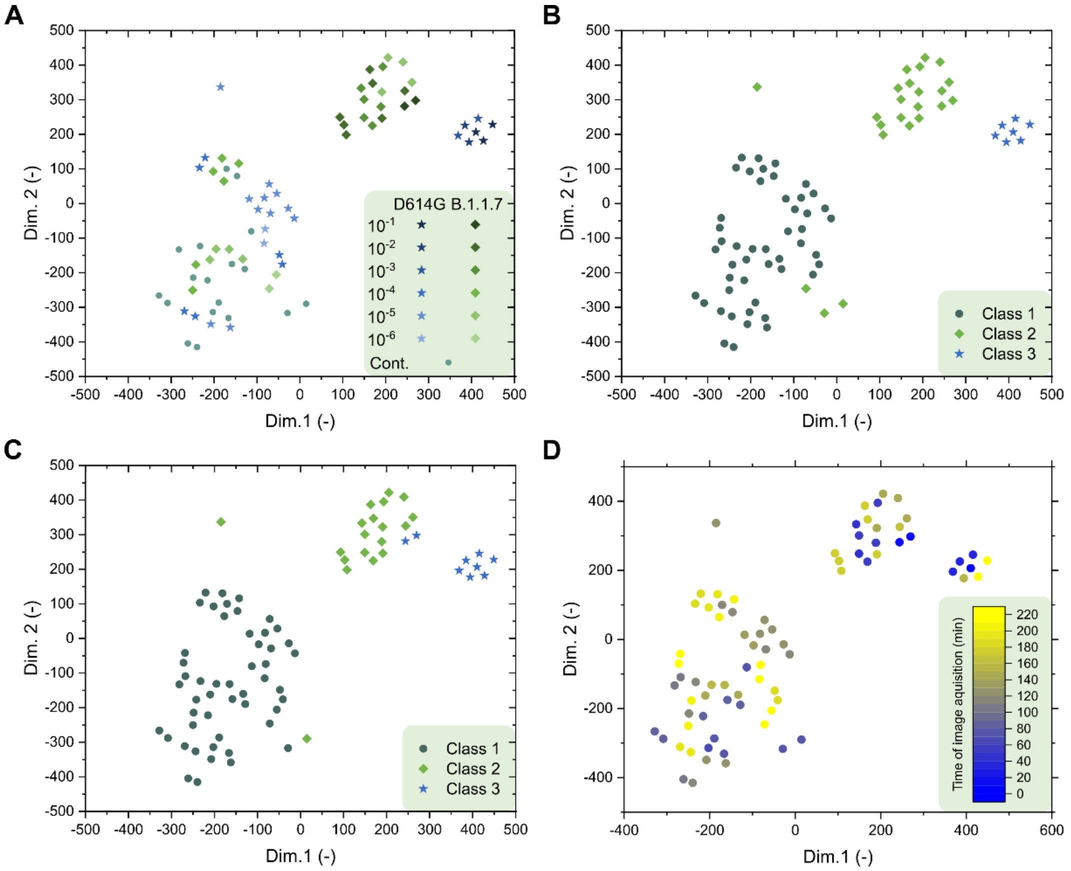

Fig. S12. T-distributed stochastic neighbor embedding (t-SNE) 2D plots obtained from all the seven image parameters recorded at 0.5 µM dye concentration. (A) t-SNE plot with virus titer $TCID_{50}$ (ml^{-1}) values and virus variant for each point. (B) t-SNE plot showing the three classes established by seven-dimensional gaussian mixture model clustering. (C) t-SNE plot showing the three classes established by seven-dimensional k-means clustering. For both clustering methods, the number of clusters was set to three. (D) The same t-SNE plot showing the temporal order of image acquisition. There is no apparent correlation between the time of acquisition and the classification of the images.

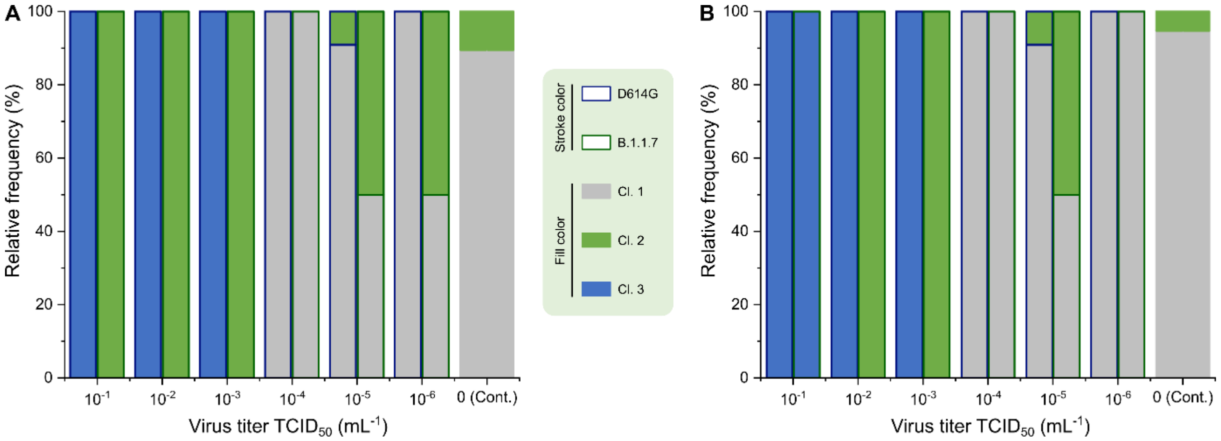

Fig. S13. Classification of the images corresponding to different variants at various virus titer in three clusters by seven-dimensional clustering. (A) Gaussian mixture model clustering. (B) K-means clustering. The virus variant and the designated cluster are indicated by the stroke color and the fill color, respectively. Both clustering analysis methods gave similar results. In detail, the three clusters in both cases match very well the following categories with a few outlier datapoints: i) no infection or low-level infection, ii) high level of D614G infection, iii) high level of B.1.1.7 infection.

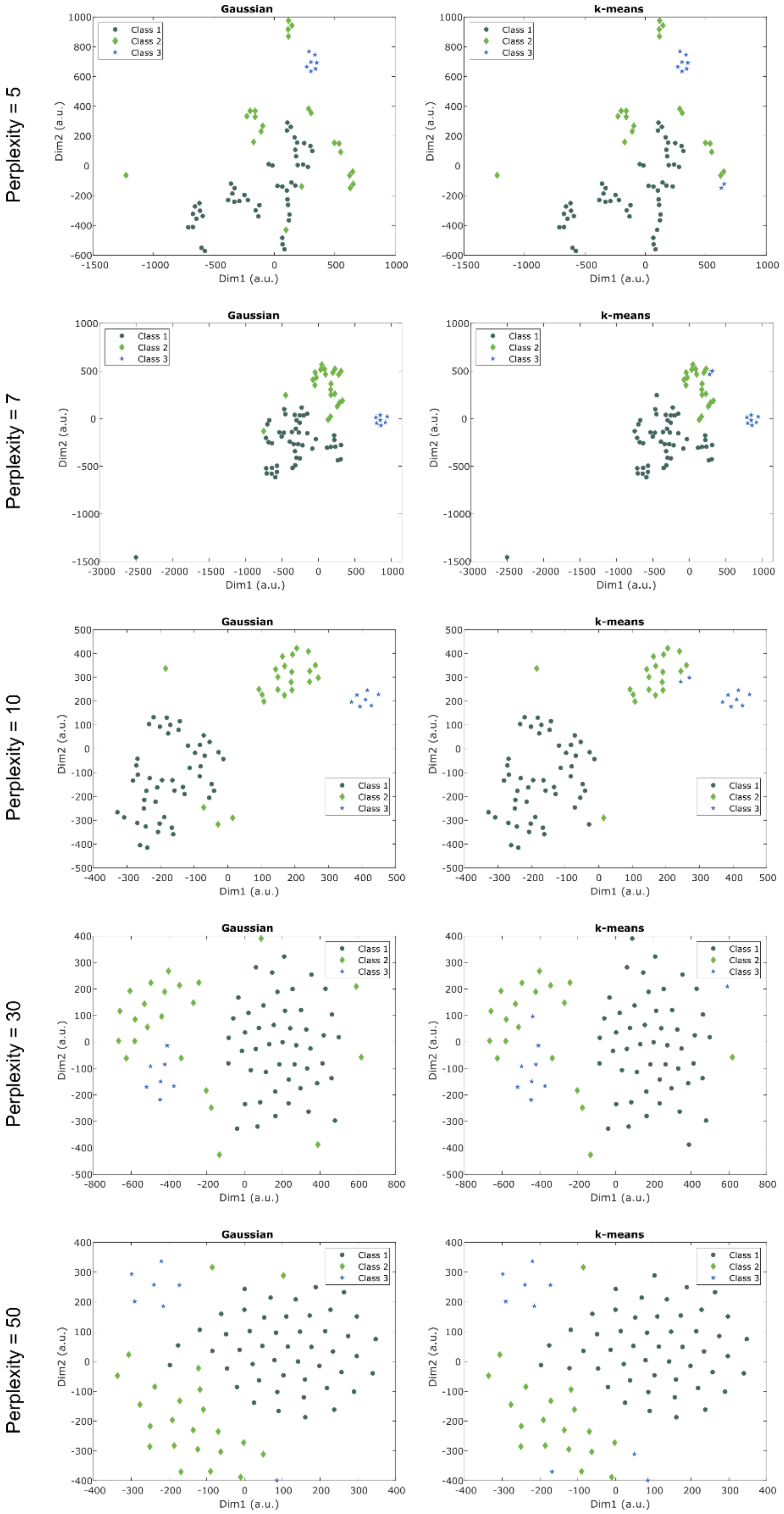

Fig. S14. Effect of the selected perplexity value on the t-SNE plot. The perplexity is an arbitrarily selected parameter used by the t-SNE algorithm that reflects the effective number of local neighbors of each point. Typical values are between 5 a nd 50, and usually larger datasets give better results with higher perplexity value. In this case, many small clusters form with the perplexity set to 5. With a value of 7, three clusters start to appear with an extreme outlier datapoint. The three clusters are the best defined and best separated at a perplexity value of 10. At higher perplexity selections of 30 and 50, the separation of clusters becomes smaller and the distances between neighboring become more uniform.

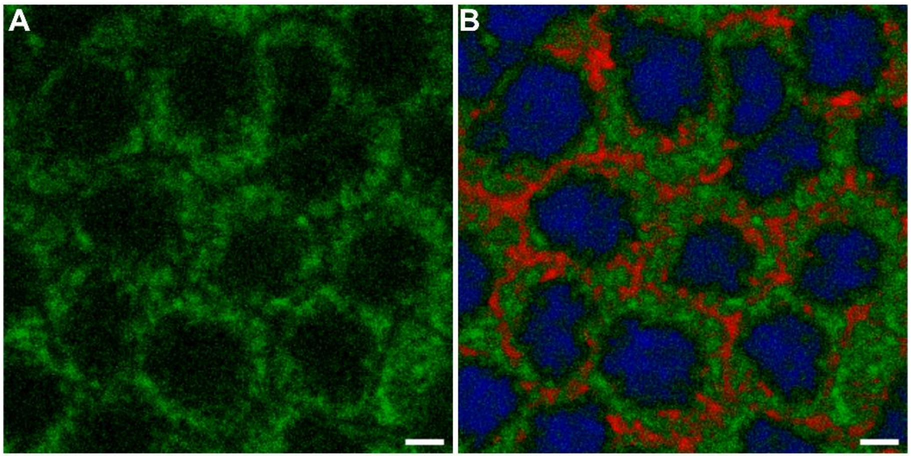

Fig. S15. The cytosolic localization of BEEF-CP visualizes the cell bodies. (A) Control Vero e6 cell culture stained with BEEF-CP yields 2P micrographs in which the individual cells are clearly distinguishable. (B) Artificial coloring used to highlight the nucleus (blue) and cell membranes (red) that are visible as regions of low fluorescence intensity in panel A. Scale bar: 10 µm.

Table S1. Image parameter values from the manual cell counting analysis. Each row represents a different image. Raw images can be found in Dataset S1.[1]

**Image ID**	Titer TCID$_{50}$ (mL^{-1})	Variant	Dye conc. (μM)	**Health**	**Initial infection**	**Infected**	**Infected vacuoles**	**Sum**	**Dead**
001	10^{-1}	D614G	0.5	0	0	73	20	93	73
003	10^{-1}	D614G	0.5	0	0	83	15	98	68
004	10^{-1}	D614G	0.05	0	0	51	15	66	100
005	10^{-1}	D614G	0.05	0	0	65	35	100	66
006	10^{-1}	D614G	0.05	1	16	48	32	97	69
007	10^{-1}	B.1.1.7	0.5	0	0	68	20	88	78
008	10^{-1}	B.1.1.7	0.5	0	0	25	22	47	119
009	10^{-1}	B.1.1.7	0.5	0	0	19	30	49	117
010	10^{-1}	B.1.1.7	0.05	21	34	8	1	64	102
014	10^{-1}	B.1.1.7	0.05	3	0	46	46	95	71
013	10^{-1}	B.1.1.7	0.05	20	0	10	30	60	106
016	10^{-2}	D614G	0.5	0	0	45	40	85	81
017	10^{-2}	D614G	0.5	0	0	77	21	98	68
019	10^{-2}	D614G	0.05	3	1	51	32	87	79
021	10^{-2}	D614G	0.05	4	7	31	34	76	90
023	10^{-2}	D614G	0.05	6	7	69	20	102	64
024	10^{-2}	B.1.1.7	0.5	0	5	31	26	62	104
027	10^{-2}	B.1.1.7	0.5	0	13	3	0	16	150
030	10^{-2}	B.1.1.7	0.5	0	8	16	15	39	127
033	10^{-2}	B.1.1.7	0.05	11	44	5	5	65	101
036	10^{-2}	B.1.1.7	0.05	20	39	11	0	70	96
039	10^{-2}	B.1.1.7	0.05	23	29	14	14	80	86
040	10^{-3}	D614G	0.5	0	0	64	5	69	97
041	10^{-3}	D614G	0.5	0	0	85	29	114	52
042	10^{-3}	D614G	10	1	0.	97	15	113	53
043	10^{-3}	D614G	0.05	0	0	82	24	106	60
045	10^{-3}	D614G	0.05	21	33	18	3	75	91
048	10^{-3}	D614G	0.05	21	9	70	17	117	49
050	10^{-3}	B.1.1.7	0.5	0	7	9	10	26	140
052	10^{-3}	B.1.1.7	0.5	18	0	11	11	40	126
054	10^{-3}	B.1.1.7	0.5	0	1	22	1	24	142
056	10^{-3}	B.1.1.7	0.05	21	14	60	53	148	18
058	10^{-3}	B.1.1.7	0.05	15	10	87	30	142	24
060	10^{-4}	D614G	0.5	181	9	0	0	190	0
062	10^{-4}	D614G	0.5	177	16	0	0	193	0
064	10^{-4}	D614G	0.5	184	4	1	0	189	0
065	10^{-4}	D614G	0.05	139	5	1	0	145	21
068	10^{-4}	D614G	0.05	139	5	1	0	115	21
070	10^{-4}	D614G	0.05	120	6	0	0	190	40
072	10^{-4}	B.1.1.7	0.5	175	8	0	0	183	0
074	10^{-4}	B.1.1.7	0.5	170	0	0	0	170	0
076	10^{-4}	B.1.1.7	0.5	176	4	0	0	180	0
078	10^{-4}	B.1.1.7	0.05	164	28	1	1	194	0

Table S1. continued

**Image ID**	Titer TCID$_{50}$ (mL^{-1})	Variant	Dye conc. (μM)	**Health**	**Initial infection**	**Infected**	**Infected vacuoles**	**Sum**	**Dead**
080	10^{-4}	B.1.1.7	0.05	138	26	20	7	191	0
083	10^{-4}	B.1.1.7	0.05	10	14	86	17	103	39
088	10^{-5}	D614G	0.5	180	2	0	0	182	0
090	10^{-5}	D614G	0.5	171	5	0	0	176	0
094	10^{-5}	D614G	0.5	161	5	1	0	167	0
097	10^{-5}	D614G	0.05	160	4	0	0	164	2
100	10^{-5}	D614G	0.05	177	2	1	0	180	0
104	10^{-5}	B.1.1.7	0.5	20	0	43	20	83	83
105	10^{-5}	B.1.1.7	0.5	163	6	0	0	169	0
107	10^{-5}	B.1.1.7	0.5	152	4	5	0	161	5
109	10^{-5}	B.1.1.7	0.05	162	8	0	0	170	0
112	10^{-5}	B.1.1.7	0.05	163	14	0	0	177	0
113	10^{-6}	D614G	0.5	166	1	0	0	167	0
116	10^{-6}	D614G	0.5	161	18	1	0	180	0
118	10^{-6}	D614G	0.05	180	9	2	0	191	0
119	10^{-6}	B.1.1.7	0.5	161	5	1	0	167	0
121	10^{-6}	B.1.1.7	0.5	164	8	1	0	173	0
118	10^{-6}	B.1.1.7	0.05	153	11	1	0	165	1
126	0	N/A	0.5	168	0	0	0	168	0
125	0	N/A	0.5	173	0	0	0	173	0
131	0	N/A	0.5	162	1	0	0	163	0
136	0	N/A	0.05	163	3	0	0	166	0
145	0	N/A	0.05	154	0	0	0	154	0
143	0	N/A	0.05	162	0	0	0	162	0

Table S2. Summary of the manual cell counting analysis.

Titer TCID$_{50}$ (mL^{-1})	Variant	Dye conc. (μM)	Mean value: Healthy	Mean value: Initial infection	Mean value: Infected	Mean value: Infected vacuoles.	Mean value: Dead	Standard deviation: Healthy	Standard deviation: Initial infection	Standard deviation: Infected	Standard deviation: Infected vacuoles.	Standard deviation: Dead
10^{-1}	D614G	0.5	0.0	0.0	69.0	16.7	80.0	0.0	0.0	16.4	2.9	17.2
		0.05	0.5	8.0	56.5	33.5	67.2	0.7	11.3	12.0	2.1	2.1
	B.1.1.7	0.5	0.0	0.0	37.3	24.0	104.3	0.0	0.0	26.7	5.3	23.1
		0.05	14.7	11.3	21.3	25.7	92.7	10.1	19.6	21.4	22.8	19.2
10^{-2}	D614G	0.5	0.0	0.0	61.0	30.5	104.7	0.0	0.0	22.6	13.4	53.2
		0.05	4.3	5.0	50.3	28.7	77.3	1.5	3.5	19.0	7.6	13.1
	B.1.1.7	0.5	0.0	8.7	16.7	13.7	126.7	0.0	4.0	14.0	13.1	23.0
		0.05	18.0	37.3	10.0	6.3	94.0	6.2	7.6	4.6	7.1	7.6
10^{-3}	D614G	0.5	0.3	0.0	82.0	16.3	74.2	0.6	0.0	16.7	12.1	31.8
		0.05	14.0	14.0	56.7	14.7	66.3	12.1	17.1	34.0	10.7	21.8
	B.1.1.7	0.5	6.0	2.7	14.0	7.3	135.7	10.4	3.8	7.0	5.5	8.7
		0.05	18.0	12.0	73.5	41.5	20.7	4.2	2.8	19.1	16.3	4.2
10^{-4}	D614G	0.5	180.7	9.7	0.3	0.0	0.0	3.5	6.0	0.6	0.0	0.0
		0.05	132.7	5.3	0.7	0.0	27.0	11.0	0.6	0.6	0.0	11.0
	B.1.1.7	0.5	173.7	4.0	0.0	0.0	0.0	3.2	4.0	0.0	0.0	0.0
		0.05	104.0	22.7	35.7	8.3	0.0	82.4	7.6	44.6	8.1	0.0
10^{-5}	D614G	0.5	170.7	4.0	0.3	0.0	0.0	9.5	1.7	0.6	0.0	0.0
		0.05	168.5	3.0	0.5	0.0	0.8	12.0	1.4	0.7	0.0	1.2
	B.1.1.7	0.5	111.7	3.3	16.0	6.7	29.1	79.6	3.1	23.5	11.5	46.4
		0.05	162.5	11.0	0.0	0.0	0.0	0.7	4.2	0.0	0.0	0.0
10^{-6}	D614G	0.5	163.5	9.5	0.5	0.0	0.0	3.5	12.0	0.7	0.0	0.0
		0.05	180.0	9.0	2.0	0.0	0.0	-[a]	-[a]	-[a]	-[a]	-[a]
	B.1.1.7	0.5	162.5	6.5	1.0	0.0	0.0	2.1	2.1	0.0	0.0	0.0
		0.05	153.0	11.0	1.0	0.0	0.7	-[a]	-[a]	-[a]	-[a]	-[a]
0	N/A	0.5	167.7	1.0	0.0	0.0	0.0	5.5	0.6	0.0	0.0	0.0
		0.05	161.7	1.7	0.0	0.0	0.0	1.5	1.5	0.0	0.0	0.0

[a]Only one image was acquired with these parameters.

Table S3. Image parameter values from the automatic image analysis. Each row represents a different image. Raw images can be found in Dataset S1.[1]

Image ID	Titer $TCID_{50}$ (mL^{-1})	Variant	PMT relative voltage (%)	Dye dilution (µM)	Relative signal area (%)	Image mean intensity (a.u.)	Mean of the threshold area (a.u.)	Maximum particle intensity (a.u.)	Average particle size (px)	Particle percentage area (%)	Particle mean intensity (a.u.)
001	10^{-1}	D614G	10	0.5	27.076	512	1387	5735	51.5	10.536	116.9
003	10^{-1}	D614G	10	0.5	30.852	574	1390	6737	57.5	12.159	118.2
008	10^{-1}	B.1.1.7	10	0.5	14.156	326	1074	5724	29.4	5.338	88.3
009	10^{-1}	B.1.1.7	10	0.5	10.875	295	1010	5365	20.8	4.371	83.8
002	10^{-1}	D614G	20	0.5	26.412	513	1431	5671	110.3	10.225	158.4
016	10^{-2}	D614G	10	0.5	35.909	602	1333	5735	55.0	13.974	115.1
017	10^{-2}	D614G	10	0.5	32.637	488	1150	5789	69.1	12.584	120.4
024	10^{-2}	B.1.1.7	10	0.5	19.153	306	783	4385	24.1	6.675	88.0
025	10^{-2}	B.1.1.7	10	0.5	5.076	213	566	2272	7.7	1.859	74.6
028	10^{-2}	B.1.1.7	10	0.5	8.040	234	740	4308	13.6	3.060	79.6
026	10^{-2}	B.1.1.7	20	0.5	4.820	217	596	2430	22.3	1.251	76.6
029	10^{-2}	B.1.1.7	20	0.5	8.002	246	775	4077	41.5	2.542	93.3
027	10^{-2}	B.1.1.7	30	0.5	4.419	217	618	2336	28.7	0.961	76.4
030	10^{-2}	B.1.1.7	30	0.5	7.508	245	787	3986	62.1	2.310	101.1
040	10^{-3}	D614G	10	0.5	34.151	524	1148	5049	44.1	12.434	105.3
041	10^{-3}	D614G	10	0.5	41.923	581	1142	5604	89.4	16.491	138.8
049	10^{-3}	B.1.1.7	10	0.5	5.839	220	745	3132	13.2	2.317	79.3
051	10^{-3}	B.1.1.7	10	0.5	7.570	238	671	3688	11.8	2.515	77.5
053	10^{-3}	B.1.1.7	10	0.5	7.767	240	904	3862	21.3	3.061	82.2
050	10^{-3}	B.1.1.7	20	0.5	5.824	231	794	3226	41.3	1.964	91.9
052	10^{-3}	B.1.1.7	20	0.5	6.503	233	727	3394	34.0	1.876	84.4
054	10^{-3}	B.1.1.7	20	0.5	7.761	245	925	3800	76.3	2.813	105.0
059	10^{-4}	D614G	20	0.5	0.136	200	354	480	5.0	0.116	69.7
061	10^{-4}	D614G	20	0.5	0.068	150	394	645	10.3	0.088	72.3
063	10^{-4}	D614G	20	0.5	0.331	179	532	1206	9.3	0.184	71.3
071	10^{-4}	B.1.1.7	20	0.5	0.011	198	349	470	3.0	0.057	67.7
073	10^{-4}	B.1.1.7	20	0.5	0.022	189	346	441	3.5	0.051	67.4
075	10^{-4}	B.1.1.7	20	0.5	0.134	196	507	962	5.5	0.155	69.2
060	10^{-4}	D614G	30	0.5	0.105	205	341	431	5.4	0.073	68.8
062	10^{-4}	D614G	30	0.5	0.063	150	372	571	15.5	0.049	73.7
064	10^{-4}	D614G	30	0.5	0.325	185	508	1102	12.3	0.161	71.7
072	10^{-4}	B.1.1.7	30	0.5	0.008	202	338	401	3.8	0.049	67.5
074	10^{-4}	B.1.1.7	30	0.5	0.011	191	341	417	4.8	0.037	66.9
076	10^{-4}	B.1.1.7	30	0.5	0.131	202	462	812	8.2	0.133	69.1
084	10^{-5}	D614G	10	0.5	0.179	171	368	726	3.5	0.992	71.6
085	10^{-5}	D614G	10	0.5	0.090	157	358	551	2.7	0.465	70.2
090	10^{-5}	D614G	10	0.5	0.189	161	494	1390	3.6	0.641	71.3
101	10^{-5}	B.1.1.7	10	0.5	11.604	243	806	3111	34.6	4.227	91.7
102	10^{-5}	B.1.1.7	10	0.5	20.042	325	904	3938	41.9	7.312	98.2
086	10^{-5}	D614G	20	0.5	0.063	172	375	612	4.2	0.194	68.6
087	10^{-5}	D614G	20	0.5	0.117	168	340	447	3.9	0.067	67.7
091	10^{-5}	D614G	20	0.5	0.113	159	546	1249	6.6	0.168	69.4

Table S3 continued

Image ID	Titer $TCID_{50}$ (mL^{-1})	Variant	PMT relative voltage (%)	Dye dilution (µM)	Relative signal area (%)	Image mean intensity (a.u.)	Mean of the threshold area (a.u.)	Maximum particle intensity (a.u.)	Average particle size (px)	Particle percentage area (%)	Particle mean intensity (a.u.)
093	10^{-5}	D614G	20	0.5	0.075	175	374	619	5.7	0.047	67.4
103	10^{-5}	B.1.1.7	20	0.5	12.014	261	835	3074	77.8	4.141	110.6
104	10^{-5}	B.1.1.7	20	0.5	20.425	341	949	3823	87.5	7.308	116.0
105	10^{-5}	B.1.1.7	20	0.5	0.102	190	435	761	8.2	0.110	69.9
107	10^{-5}	B.1.1.7	20	0.5	0.044	186	402	886	7.2	0.066	71.0
088	10^{-5}	D614G	30	0.5	0.048	170	348	485	5.8	0.222	67.5
089	10^{-5}	D614G	30	0.5	0.019	164	330	366	4.1	0.016	66.0
092	10^{-5}	D614G	30	0.5	0.171	160	619	4018	7.5	0.151	73.4
094	10^{-5}	D614G	30	0.5	0.098	178	359	505	8.0	0.029	66.5
106	10^{-5}	B.1.1.7	30	0.5	0.087	194	398	594	11.6	0.080	71.6
108	10^{-5}	B.1.1.7	30	0.5	0.026	191	383	672	8.0	0.033	70.6
113	10^{-6}	D614G	20	0.5	0.106	207	425	662	4.3	0.073	68.4
115	10^{-6}	D614G	20	0.5	0.060	171	419	645	4.7	0.142	69.4
114	10^{-6}	D614G	30	0.5	0.092	211	398	554	5.4	0.040	67.9
116	10^{-6}	D614G	30	0.5	0.058	171	406	584	9.0	0.067	69.9
119	10^{-6}	B.1.1.7	30	0.5	0.054	161	455	764	17.4	0.032	72.0
120	10^{-6}	B.1.1.7	40	0.5	0.037	161	442	645	40.3	0.024	79.1
123	Reference	N/A	10	0.5	0.133	165	496	1063	3.2	0.646	71.5
124	Reference	N/A	10	0.5	0.190	155	453	3415	3.4	0.630	71.6
129	Reference	N/A	10	0.5	0.359	187	435	1150	3.1	0.491	71.1
125	Reference	N/A	20	0.5	0.093	168	536	906	5.5	0.167	69.6
126	Reference	N/A	20	0.5	0.124	163	411	962	5.9	0.157	69.4
130	Reference	N/A	20	0.5	0.375	189	444	1117	11.2	0.160	75.5
131	Reference	N/A	20	0.5	0.051	193	408	563	10.6	0.060	72.4
144	Reference	N/A	20	0.5	0.047	200	404	1044	3.6	0.121	68.2
146	Reference	N/A	20	0.5	0.062	189	702	1747	4.4	0.081	69.7
147	Reference	N/A	20	0.5	0.016	192	330	361	3.2	0.093	68.0
150	Reference	N/A	20	0.5	0.060	199	457	1084	5.1	0.048	70.3
127	Reference	N/A	30	0.5	0.075	165	494	708	7.9	0.124	68.4
128	Reference	N/A	30	0.5	0.094	162	425	2112	7.0	0.093	72.1
132	Reference	N/A	30	0.5	0.381	198	426	962	17.6	0.101	77.2
133	Reference	N/A	30	0.5	0.043	198	392	526	12.4	0.037	70.4
145	Reference	N/A	30	0.5	0.051	204	393	934	5.0	0.077	68.2
148	Reference	N/A	30	0.5	0.051	193	704	1566	5.4	0.057	68.7
149	Reference	N/A	30	0.5	0.006	196	324	335	3.7	0.034	66.9
151	Reference	N/A	30	0.5	0.079	204	401	901	7.4	0.030	70.5
004	10^{-1}	D614G	10	0.05	15.320	223	476	1346	13.2	4.112	80.3
005	10^{-1}	D614G	10	0.05	17.002	219	459	1183	17.7	5.264	81.5
006	10^{-1}	D614G	10	0.05	25.483	259	443	1183	11.7	6.250	79.3
007	10^{-1}	B.1.1.7	10	0.05	26.296	456	1144	6094	42.7	9.644	101.9
010	10^{-1}	B.1.1.7	10	0.05	1.542	158	405	933	9.4	0.974	75.2
011	10^{-1}	B.1.1.7	10	0.05	12.181	254	548	2577	10.9	3.461	77.7
014	10^{-1}	B.1.1.7	10	0.05	13.541	211	450	1488	10.8	3.594	78.5
012	10^{-1}	B.1.1.7	20	0.05	1.189	150	409	843	16.8	0.551	75.5

Table S3 continued

Image ID	Titer TCID$_{50}$ (mL^{-1})	Variant	PMT relative voltage (%)	Dye dilution (μM)	Relative signal area (%)	Image mean intensity (a.u.)	Mean of the threshold area (a.u.)	Maximum particle intensity (a.u.)	Average particle size (px)	Particle percentage area (%)	Particle mean intensity (a.u.)
013	10^{-1}	B.1.1.7	20	0.05	0.000	113	323	325	3.4	0.004	66.1
015	10^{-1}	B.1.1.7	20	0.05	11.896	202	455	1361	17.4	2.559	75.9
018	10^{-2}	D614G	10	0.05	11.899	194	431	1118	11.7	3.766	78.1
020	10^{-2}	D614G	10	0.05	7.604	182	427	965	10.0	2.381	77.4
022	10^{-2}	D614G	10	0.05	14.106	194	453	1858	16.3	4.603	81.4
031	10^{-2}	B.1.1.7	10	0.05	0.945	143	375	704	6.9	0.811	74.0
034	10^{-2}	B.1.1.7	10	0.05	3.280	171	387	737	6.9	1.502	74.4
037	10^{-2}	B.1.1.7	10	0.05	8.701	195	428	1325	9.0	2.531	76.6
019	10^{-2}	D614G	20	0.05	11.167	190	435	1094	18.7	2.838	76.2
021	10^{-2}	D614G	20	0.05	6.804	174	428	909	15.7	1.612	74.5
023	10^{-2}	D614G	20	0.05	13.932	194	452	1193	23.6	3.776	78.0
032	10^{-2}	B.1.1.7	20	0.05	0.770	147	371	678	10.9	0.434	71.2
035	10^{-2}	B.1.1.7	20	0.05	2.889	167	389	724	11.4	0.858	72.3
038	10^{-2}	B.1.1.7	20	0.05	7.344	187	436	1391	14.4	1.563	74.2
033	10^{-2}	B.1.1.7	30	0.05	0.503	145	361	571	11.5	0.204	69.7
036	10^{-2}	B.1.1.7	30	0.05	2.480	166	385	700	11.7	0.470	70.1
039	10^{-2}	B.1.1.7	30	0.05	6.232	183	428	1127	15.7	0.967	72.4
042	10^{-3}	D614G	10	0.05	18.344	219	446	1052	13.9	5.063	79.8
043	10^{-3}	D614G	10	0.05	17.480	213	449	1281	15.0	5.088	80.8
045	10^{-3}	D614G	10	0.05	4.184	169	395	1041	6.5	1.162	75.1
047	10^{-3}	D614G	10	0.05	16.401	205	465	1205	17.9	4.987	81.9
055	10^{-3}	B.1.1.7	10	0.05	14.771	209	421	1902	10.8	4.630	77.9
057	10^{-3}	B.1.1.7	10	0.05	17.851	215	442	1172	13.8	5.363	79.9
044	10^{-3}	D614G	20	0.05	16.067	204	438	1011	19.5	3.751	76.3
046	10^{-3}	D614G	20	0.05	3.670	159	395	24531	10.4	0.523	84.9
048	10^{-3}	D614G	20	0.05	16.181	200	462	1081	26.7	4.288	79.7
056	10^{-3}	B.1.1.7	20	0.05	14.315	207	423	1262	17.4	3.423	75.0
058	10^{-3}	B.1.1.7	20	0.05	16.959	210	440	1074	20.5	4.133	77.5
081	10^{-4}	B.1.1.7	10	0.05	17.838	214	498	2827	18.1	5.222	82.9
065	10^{-4}	D614G	20	0.05	0.205	152	436	853	7.2	0.177	70.1
067	10^{-4}	D614G	20	0.05	0.102	152	539	1114	13.3	0.086	71.7
069	10^{-4}	D614G	20	0.05	3.798	145	416	893	17.6	0.981	75.1
077	10^{-4}	B.1.1.7	20	0.05	0.257	142	376	569	9.5	0.184	70.1
079	10^{-4}	B.1.1.7	20	0.05	1.426	138	380	662	8.1	0.280	71.0
082	10^{-4}	B.1.1.7	20	0.05	17.814	207	468	1077	24.5	4.321	78.5
066	10^{-4}	D614G	30	0.05	0.219	152	420	777	12.2	0.109	74.5
068	10^{-4}	D614G	30	0.05	0.086	152	507	947	20.3	0.065	71.6
070	10^{-4}	D614G	30	0.05	3.503	146	403	772	18.5	0.689	72.7
078	10^{-4}	B.1.1.7	30	0.05	0.159	142	379	528	11.4	0.077	69.5
080	10^{-4}	B.1.1.7	30	0.05	1.440	139	375	645	8.4	0.160	68.6
083	10^{-4}	B.1.1.7	30	0.05	16.653	201	448	1002	25.0	3.269	75.6
095	10^{-5}	D614G	20	0.05	0.042	150	516	919	7.6	0.114	71.3
097	10^{-5}	D614G	20	0.05	0.093	153	489	856	9.6	0.086	73.0
098	10^{-5}	D614G	20	0.05	0.048	153	445	695	5.6	0.114	68.9

Table S3 continued

Image ID	Titer TCID$_{50}$ (mL^{-1})	Variant	PMT relative voltage (%)	Dye dilution (µM)	Relative signal area (%)	Image mean intensity (a.u.)	Mean of the threshold area (a.u.)	Maximum particle intensity (a.u.)	Average particle size (px)	Particle percentage area (%)	Particle mean intensity (a.u.)
109	10^{-5}	B.1.1.7	20	0.05	0.025	150	407	546	7.6	0.056	71.1
111	10^{-5}	B.1.1.7	20	0.05	0.065	156	444	764	7.7	0.100	70.7
096	10^{-5}	D614G	30	0.05	0.038	153	468	800	8.8	0.094	69.8
099	10^{-5}	D614G	30	0.05	0.075	153	453	746	11.2	0.073	70.4
100	10^{-5}	D614G	30	0.05	0.039	153	423	615	8.8	0.048	70.2
110	10^{-5}	B.1.1.7	30	0.05	0.019	150	408	2417	7.5	0.034	77.2
112	10^{-5}	B.1.1.7	30	0.05	0.044	154	428	647	9.1	0.069	69.7
117	10^{-6}	D614G	30	0.05	0.045	118	501	742	94.3	0.029	100.0
121	10^{-6}	B.1.1.7	30	0.05	0.004	147	343	373	7.3	0.018	68.1
118	10^{-6}	D614G	40	0.05	0.039	118	467	647	86.3	0.026	80.4
122	10^{-6}	B.1.1.7	40	0.05	0.000	145	330	332	6.3	0.017	67.6
134	Reference	N/A	10	0.05	0.003	149	330	355	2.5	0.308	70.3
136	Reference	N/A	20	0.05	0.078	152	562	1341	6.4	0.149	71.3
138	Reference	N/A	20	0.05	0.076	142	405	586	14.6	0.069	74.8
140	Reference	N/A	20	0.05	0.017	150	478	695	4.2	0.106	68.3
141	Reference	N/A	20	0.05	0.016	144	355	424	6.1	0.050	69.8
137	Reference	N/A	30	0.05	0.001	151	327	328	3.7	0.017	66.6
139	Reference	N/A	30	0.05	0.064	142	376	480	21.4	0.043	78.9
142	Reference	N/A	30	0.05	0.014	151	455	624	6.5	0.050	68.3
143	Reference	N/A	30	0.05	0.007	146	350	386	5.7	0.024	67.0

Table S4. Comparison of in vitro cell culture assays of SARS-CoV-2 infection.

Method	Advantages	Disadvantages	Reference
Quantitative polymerase chain reaction (qPCR)	• Robust<br>• Quick<br>• Quantitative information	• No cellular level information	[2,3]
Immunofluorescence (IF) staining	• Cellular level information<br>• Highly specific signal	• Expensive fluorescent antibodies<br>• Only works after fixation	[4,5]
SARS-CoV-2 GFP/ΔN fluorescence imaging	• Cellular level information<br>• Highly specific signal<br>• Imaging of the infection in live cells<br>• BSL2 environment	• Only works on transgenic cell lines<br>• GFP gene deletion over time	[6]
2P calcium imaging	• Cellular level information<br>• Imaging of the infection in live cells<br>• High 3D spatial resolution – subcellular information	• Expensive instrumentation<br>• Derivative signal	This work

Immunofluorescent (IF) and plaque assays do not fulfil the aims of modern virology and especially do not provide any reproducible and cellular data quantitative means of the same virally infected culture over time. ELISA assays do not provide enough granularity and cannot inform about in-culture spread of the infection. qPCR assays also come with the need of destroying the samples, but most importantly, no host and functional changes are quantified using qPCR in infected cultures. Single cell sequencing[7] allows the tracking of viral sequences and host transcriptome but it comes with the necessary destruction of the investigated sample cellular/organoid entity and there is a large variability by cell, whereas this method inherently destroys the surface overview of cellular spread within the tissue/organoid/cell culture. There is also no possibility of longitudinal investigation of a model organoid over time. Our methodology crucially employs a novel calcium-sensitive dye with unique cell-penetration and sensitivity. This dye can be applied in longitundinal studies and in cell or organoid cultures. As shown in our study, when the BEEF-CP dye is coupled to two-photon microscopy with high spatial and temporal resolution, quantitation with appropriate software and analysis reveals surprising distinction possibilities. Our approach was to analyse numerous parameters within individual cells. This technique allows for the simultaneous assessment of viral replication, host cell responses, and morphological changes, providing a comprehensive understanding of virus infection dynamics in cell culture.

ImageJ script

```
run("Set Measurements...", "area mean min area_fraction limit display redirect=None decimal=2");
run("Clear Results");

//select directory of images to open
input = getDirectory("Input directory where images are stored");

//select location where images/results are to be stored
output = getDirectory("Output ditrectory for results");

//gets list of files
list = getFileList(input);

//arrays
Image_mean=newArray(list.length);

//loop for opening images
for (image=0;image<list.length;image++){
full = input +  list[image];
open(full);

fn=getTitle();
roiManager("reset");
run("Select None");

run("Duplicate...", "title=mask duplicate");
run("Despeckle", "stack");
//run("Threshold...");
setThreshold(320, 65535);//set threshold limit for image set
run("Measure");
Image_mean[image]=getValue("Mean");

run("Create Selection");
if (selectionType!=-1){
roiManager("Add");
}

//particle measure
selectWindow("mask");
resetThreshold();
run("Select None");
run("Duplicate...", "title=mask2");
run("Gaussian Blur...", "sigma=5");//blurr command
imageCalculator("Subtract create", "mask","mask2");
selectWindow("Result of mask");
rename(fn+" particles");
setThreshold(62, 65535);//set threshold limit for image set
run("Analyze Particles...", "summarize exclude");

run("Create Selection");
if (selectionType!=-1){
roiManager("Add");
}

selectWindow(fn);
roiManager("select", 0);
roiManager("Set Color", "red");
roiManager("select", 1);
roiManager("Set Fill Color", "green");
roiManager("Show All without labels");

//save drawing or image
out = output + fn + " Overlay";
saveAs("Tiff",out);
run("Close All");
}

Thresholded_mean=Table.getColumn("Mean");
Percent_area=Table.getColumn("%Area");
Max_Intensity=Table.getColumn("Max");
selectWindow("Results");
```

```
run("Close");

Table.create("Image data");
Table.setLocationAndSize(200, 200, 800, 600);
Table.setColumn("Image", list);
Table.setColumn("Signal % area", Percent_area);
Table.setColumn("Image Mean intensity", Image_mean);
Table.setColumn("Mean of thresholded area", Thresholded_mean);
Table.setColumn("Max intensity", Max_Intensity);

//save results file
selectWindow("Image data");
out = output + "Image data.xls";
saveAs("Results",out);

selectWindow("Summary");
IJ.renameResults("particle data");
out = output + " particle data.xls";
saveAs("Results",out);

run("Set Measurements...", "area mean min area_fraction display redirect=None decimal=2");
```

Microbiological assay results

Cytotoxicity assay

The toxicity characteristic of the Ca-ion sensor BEEF-CP was studied by cell viability experiments on two cell lines, such as A431 and HEK, showed in **Figure S13**, on a Perkin Elmer EnSpire Multimode Plate Reader with the standard protocol (PrestoBlu Cell Viability Reagent from Invitrogen, 10 min incubation). Parallel with the dye, the solvent applied (DMSO) was also included in the studies as reference. For both cell line, the toxicity trends break down above 8 μM dye concentration and the IC50 values are around 11 and 15 μM. These concentration values, however, are much higher than the concentration applied during this study (final concentration of BEEF-CP is 0.5 μM and 0.05 μM, as using 10 μl of dye (10 μM or 1 μM) in original stock, diluted to 200 μl in the vial, respectively), consequently we are at the non-toxic region.

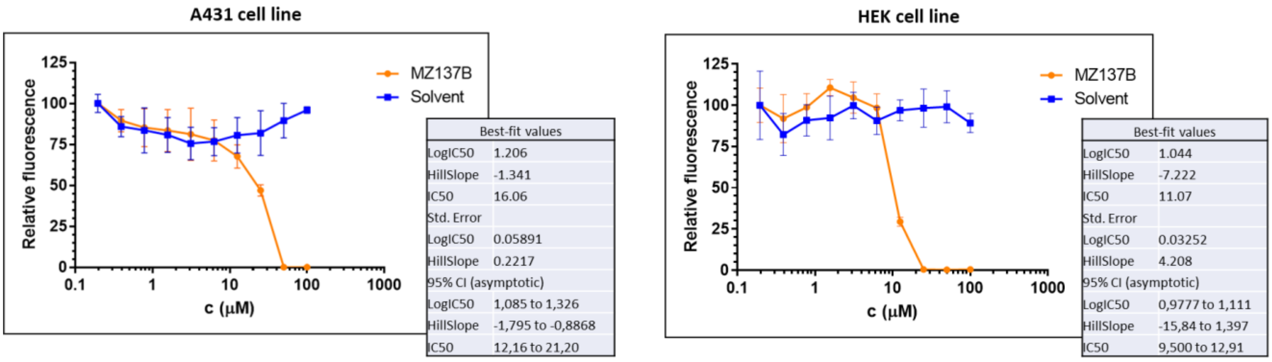

Fig S16. Graphical representation of cytotoxicity assays on A431 (left) and HEK (right) cell lines. The orange curves summarize the measured cell viability values with different concentration of calcium indicator (BEEF-CP) in comparison with the parallel experiments solvent applied (DMSO).

Parallelly to the cell viability experiment, an alternate cytotoxicity assay using uninfected 80% confluence VeroE6 cells incubated for 24 hours in the presence of the medium containing 0.5 μM concentration BEEF-CP solution, a dedicated spectrophotometer (Omni cell adhesion light spectrometer, Cytosmart, Netherlands) was used. The percentage rate of surface covered by monolayers and of transparent surface (plaques appearing in the absence of cells killed by the dye) in all the wells of the 96-well cell culture platform were determined. All tests were made in triplicates.

Flow cytometry measurement results

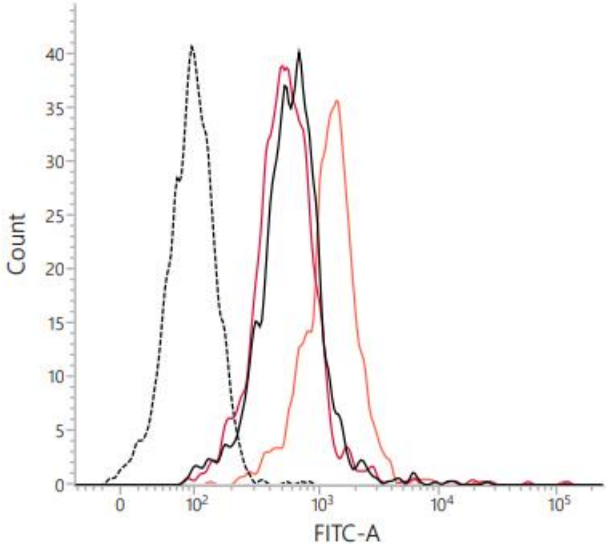

Fig. S17. Results of the flow cytometry measurement. Dotted black line: untreated cells; Solid black line: BEEF-CP treated cells; solid orange line: ionomycin + BEEF-CP treated cells; solid red line: EGTA + BEEF-CP treated cells.

Table S5. Tabulated summary of the flow cytometry measurements.

	**Fluorescence Intensity Mean (a.u.)**			**Ratio (-)**		
	**Untreated**	**+Ionomycin**	**+EGTA**	**Ionomycin/ Untreated**	**EGTA/ Untreated**	**(Ion./Unt.)/ (EGTA/Unt.)**
**HEK-293 control**	108	189	104	1.75	0.96	1.82
**HEK-293 + BEEF-CP**	784	1601	911	2.04	1.16	1.76

Synthetic methods and results

General synthetic methodologies and characterization

All the starting materials, reagents and solvents were purchased from Sigma-Aldrich (Merck) in reagent grade and used as received. NMR solvents were purchased from Eurisotop. Reactions were monitored by LC-MS (Shimazu MS2020, Supelco Ascentis, 2.0 × 50 mm, 2.1 µm C18 column; injection of 1 µl; 5–98% MeCN/H_2O, linear gradient, with constant 0.1% v/v TFA additive; run of 6 min; flow of 0.8 ml min^{-1}; ESI; positive ion mode). Reaction products were purified by gradient elution preparative HPLC (HPLC Gilson 333 instrument, UV detector 220 nm) on a Phenomenex Gemini C18, 250×50.00 mm; 10 µm, 110A column using 0.2% v/v TFA in water and acetonitrile as the mobile phase components.

^{1}H NMR and ^{13}C NMR spectra were recorded at 600/400 MHz, 151/101 MHz on Bruker Avance 600 or 400 spectrometers. All chemical shifts are quoted in parts per million (ppm), measured from the center of the signal except in the case of multiplets, which are quoted as a range. ^{1}H NMR and ^{13}C chemical shifts are referenced to the residual solvent peak of $(CD_3)_2SO$ (^{1}H referenced to 2.50 ppm and ^{13}C referenced to 39.52 ppm) or $CDCl_3$ (^{1}H referenced to 7.26 ppm and ^{13}C referenced to 77.16 ppm). Coupling constants are given with an accuracy of 0.1 Hz. Splitting patterns are abbreviated as follows: singlet (s), doublet (d), triplet (t), quartet (q), multiplet (m), broad singlet (bs) and combinations thereof. Assignment of spectra was aided by 2D NMR spectroscopy (^{1}H-^{13}C HSQC and HMBC).

HRMS and MS-MS analyses were performed on a Thermo Velos Pro Orbitrap Elite (Thermo Fisher Scientific) system. The ionization method was ESI operated in positive (or negative) ion mode. The protonated (or deprotonated) molecular ion peaks were fragmented by collision-induced dissociation (CID) at a normalized collision energy of 40-65%. For the CID experiment helium was used as the collision gas. The samples were dissolved in methanol. Data acquisition and analysis were accomplished with Xcalibur software version 4.0 (Thermo Fisher Scientific).

Synthesis of BEEF-CP

The preparation of the calcium dye was carried out in a five-step synthetic route using 4-ethyl resorcinol, 1,2,4-benzenetricarboxylic anhydride and tetraethyl BAPTA[8] in high overall yield.

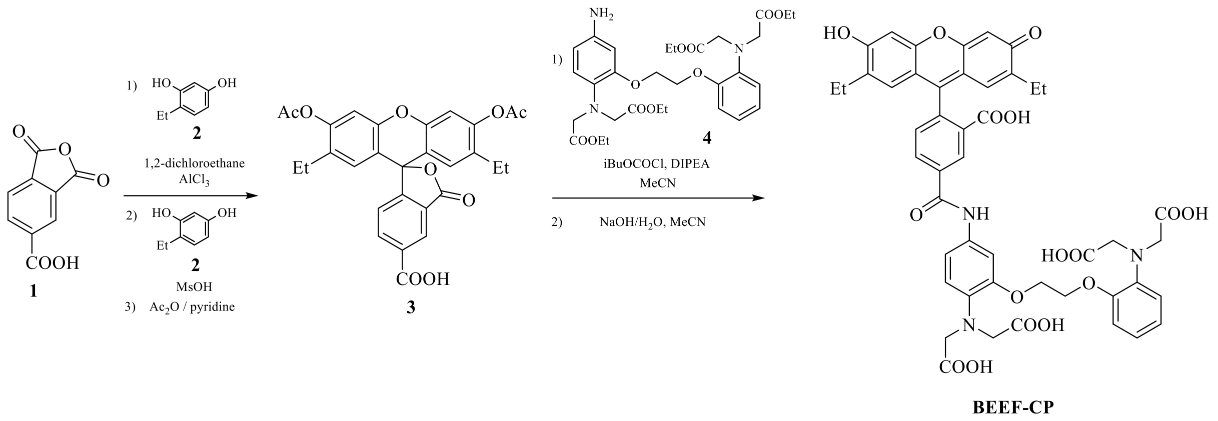

Synthesis of ketone 5

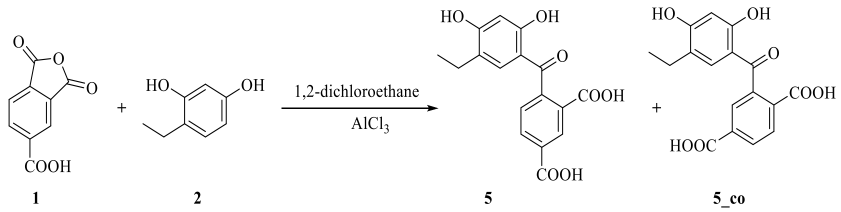

4-Ethylresorcinol (**2**, 1.38 g, 10.0 mmol, 1.0 equiv.) and 1,2,4-benzenetricarboxylic anhydride (**1**, 2.11 g, 11.0 mmol, 1.1 equiv.) were dissolved in 1,2-dichloroethane (55.0 mL). $AlCl_3$ (7.99 g, 60 mmol, 6.0 equiv.) was added, the mixture was stirred for 72 hours at room temperature. The solvent was evaporated, 100 mL of EtOAc, then 50 mL of aqueous HCl (4M) were added then the layers were separated. The aqueous phase was washed with EtOAc (3 × 100 mL), the combined organic layers was washed with aqueous HCl (1M, 50 mL), brine (50 mL), dried over $MgSO_4$. The solvent was evaporated under reduced pressure. The residue (3.51 g) was purified by preparative HPLC (water–acetonitrile–0.1% TFA, using the gradient method). After purification, the fractions were lyophilized. The target isomer (**5**, 1.49 g, yield 45%) and its regioisomer (**5_co** 1.02 g, yield 31%) were isolated as a yellow powder.

Spectroscopic data of ***5***

^{1}H NMR (400 MHz, DMSO-*d6*) δ 13.26 (s, 2H), 11.86 (s, 1H), 10.62 (s, 1H), 8.50 (d, J = 1.6 Hz, 1H), 8.21 (dd, J = 7.9, 1.7 Hz, 1H), 7.54 (d, J = 7.9 Hz, 1H), 6.80 (s, 1H), 6.39 (s, 1H), 2.34 (q, J = 7.4 Hz, 2H), 0.95 (t, J = 7.5 Hz, 3H). ^{13}C NMR (101 MHz, DMSO-d_6) δ 198.9, 166.0, 163.1, 162.4, 143.8, 132.6, 132.4, 131.8, 130.5, 130.0, 127.9, 122.7, 112.7, 102.2, 21.7, 13.8. HR-MS:(ESI) calcd. for $C_{17}H_{13}O_7$ 329.0667 $[M-H]^+$, found 329.06700, Df. = 1.0 ppm. HR-ESI-MS-MS (CID=40%; rel. int. %): 285(2); 191(100) and 147(9).

Spectroscopic data of ***5_co***

^{1}H NMR (400 MHz, DMSO-d_6) δ 8.15 (dd, J = 8.1, 1.7 Hz, 1H), 8.06 (d, J = 8.1 Hz, 1H), 7.85 (d, J = 1.6 Hz, 1H), 6.83 (s, 1H), 6.39 (s, 1H), 2.35 (q, J = 7.4 Hz, 2H), 0.96 (t, J = 7.5 Hz, 3H). ^{13}C NMR (101 MHz, DMSO-d_6) δ 198.7, 166.4, 166.0, 163.0, 162.5, 140.2, 133.8, 133.8, 132.4, 130.2, 130.1, 127.9, 122.6, 112.8, 102.2, 21.7, 13.8.

Synthesis of unprotected fluorescein derivative 6

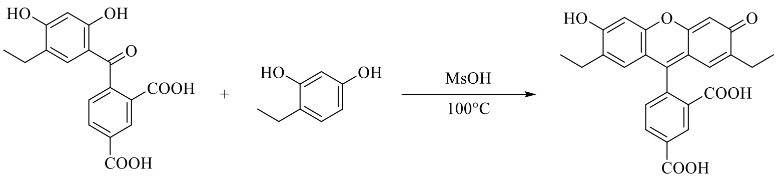

5 **2** **6**

5 (0.90 g, 2.73 mmol, 1.0 equiv.) and 4-ethylresorcinol (**2**, 628 mg, 3.27 mmol, 1.2 equiv.) were dissolved in methanesulfonic acid (6 mL) and stirred for 2 hours at 100 °C. The mixture was poured carefully to crushed ice (100 g), and the formed solid orange precipitate was filtered off and dried. 1.12 g (95%) orange solid product (**6**) was formed and used without further purification.

^{1}H NMR (400 MHz, DMSO-d_6) δ 10.11 (bs, 1H), 8.42 (s, 1H), 8.29 (dd, J = 8.0, 1.5 Hz, 1H), 7.38 (d, J = 8.0 Hz, 1H), 6.74 (bs, 2H), 6.45 (bs, 2H), 2.36 (q, J = 7.5 Hz, 4H), 0.93 (t, J = 7.5 Hz, 6H). ^{13}C NMR (101 MHz, DMSO-d_6) δ 167.8, 166.1, 156.1, 150.3, 135.9, 132.7, 129.1, 127.6, 120.3, 102.3, 101.8, 22.4, 14.1. HR-MS:(ESI) calcd. for $C_{25}H_{21}O_7$ 433.12818 [M+H]$^+$, found 433.12787, Df. = -0.7 ppm. HR-ESI-MS-MS (CID=65%; rel. int. %): 418(100); 405(25); 390(20) and 373(20).

Acylation of the fluoresceine derivative 6

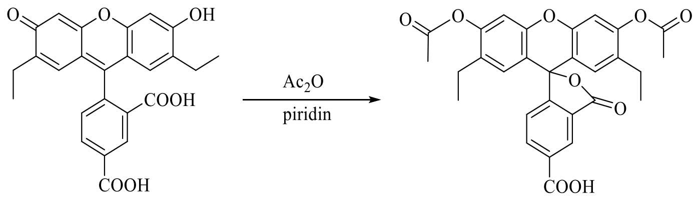

6 **3**

6 (1.10 g, 2.54 mmol, 1.0 equiv.) was dissolved in acetic anhydride (3.50 mL, 3 equiv.) and pyridine (0.71 mL) was added than the mixture was stirred at 100 °C for 60 minutes. The mixture was cooled down to room temperature than poured carefully to crashed ice (100 g). The product was extracted with EtOAc (3 × 100 mL), the combined organic phases was washed with distilled water (2 × 200 mL), saturated sodium carbonate (2 × 200 mL) aqueous hydrochloric acid (1M, 1 × 50 mL), distilled water (1 × 200 mL) and brine (1 × 100 mL). The solvent was dried over $MgSO_4$, evaporated under reduced pressure. 621 mg (47%) product (**3**) was isolated as a yellow solid.

^{1}H NMR (400 MHz, DMSO-d_6) δ 13.63 (bs, 1H), 8.27 (dd, J = 7.9, 1.2 Hz, 1H), 8.18 (dd, J = 7.9, 0.8 Hz, 1H), 7.78 (dd, J = 1.2, 0.8 Hz, 1H), 7.25 (s, 2H), 6.76 (s, 2H), 2.38 (m, 4H), 2.33 (s, 6H), 0.92 (t, J = 7.5 Hz, 6H). ^{13}C NMR (101 MHz, DMSO-d_6) δ 168.9, 167.7, 166.0, 152.4, 150.3, 148.9, 137.5, 132.3, 131.3, 128.9, 128.1, 125.7, 124.5, 115.8, 111.2, 81.6, 22.1, 20.6, 14.3. HR-MS:(ESI) calcd. for $C_{29}H_{26}O_9$ 517.14931 [M+H]$^+$, found 517.14944.

N-acylation of amino BAPTA moiety using 3

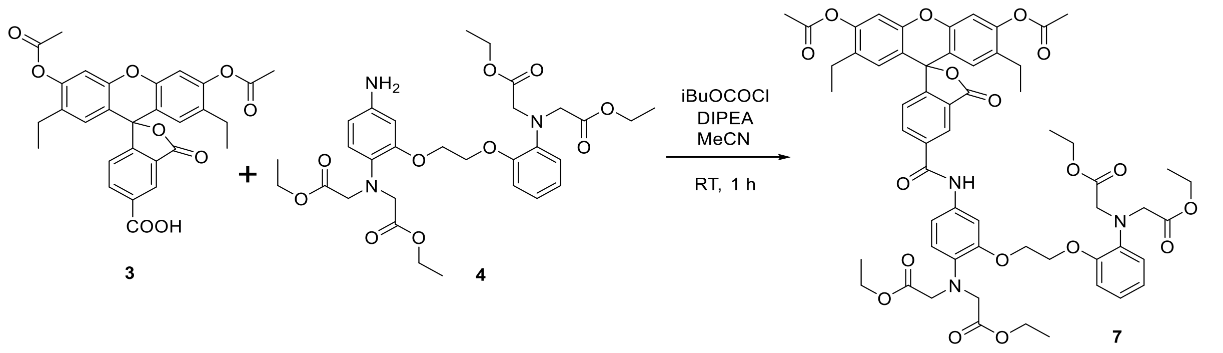

Tetraethyl ester **3** (156 mg, 0.302 mmol, 1.0 equiv.) was dissolved in dry dichloromethane (5 mL) diisopropylethylamine (0.049 mL, 0.605 mmol, 2.0 equiv.) was added. The mixture was cooled down to 0°C. Isobutyl chloroformate (0.038 mL, 0.29 mmol, 0.95 equiv.) was added, and the mixture was stirred at room temperature for 60 minutes. 219 mg (0.363 mmol, 1.2 equiv.) of amino BAPTA derivative (**4**) [9] was added, and the mixture was stirred overnight. The mixture was poured into distilled water (40 mL), the product was extracted with EtOAc (6 × 50 mL), the combined organic phases was washed with saturated sodium hydrogen carbonate (1 × 50 mL) and brine (1 × 100 mL). The solvent was dried over $MgSO_4$, evaporated under reduced pressure. The residue (405 mg) was purified by preparative HPLC (water–acetonitrile–0.1% TFA, using the gradient method). After purification, the fractions were lyophilized. The product (**7,** 170 mg, yield 51%) was isolated as a yellow powder.

^{1}H NMR (400 MHz, $CDCl_3$) δ 8.69 (bs, 1H), 8.56 (bs, 1H), 8.58 (s, 1H) 8.29 (dd, *J* = 8.0, 1.7 Hz, 1H), 8.21 (m, 1H), 8.11 – 8.01 (m, 5H), 7.54 – 7.49 (m, 1H), 7.36 – 7.27 (m, 2H), 7.03 (s, 1H), 6.91 – 6.82 (m, 2H), 6.62 (s, 1H), 4.15 – 4.13 (m, 4H), 4.67 (s, 8H), 4.16 (qd, *J* = 7.4 Hz, 3.0 Hz, 8H), 2.45 – 2.36 (m, 4H), 2.36 – 2.32 (m, 6H), 1.25 – 1.12 (m, 12H), 1.01 (t, *J* = 7.5 Hz, 6H). ^{13}C NMR (101 MHz, $CDCl_3$) δ 172.0, 171.7, 169.1, 168.8, 165.6, 165.6, 155.7, 150.6, 150.5, 150.4, 149.8, 137.4, 135.0, 134.3, 133.8, 133.8, 132.4, 131.4, 130.4, 129.9, 129.8, 129.0, 128.2, 126.7, 124.9, 121.7, 119.4, 119.3, 115.9, 113.2, 111.3, 63.2, 63.1, 61.2, 61.1, 53.9, 23.2, 21.0, 14.3, 14.2, 14.2. HR-MS:(ESI) calcd. for $C_{59}H_{64}N_3O_{18}$ 1102.41794 $[M+H]^+$, found 1102.41960. HR-ESI-MS-MS (CID=40%; rel. int. %): 1060(25); 1028(100) and 986(8).

Hydrolysis of 7 using aqueous sodium hydroxide

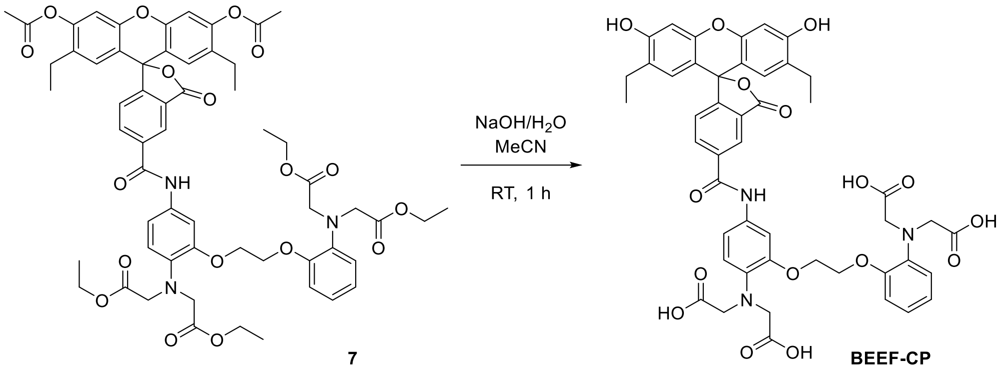

7 **BEEF-CP**

7 (130 mg, 0.118 mmol, 1.0 equiv.) was dissolved in methanol (2 mL). Sodium hydroxide (50 mg, 125 mmol, 11 equiv.) in water (0.40 mL) was added at 0 °C, the mixture was stirred at room temperature for 60 minutes. The mixture was diluted to 5 mL with ethanol/acetonitrile 1/1 and was purified by preparative HPLC (water–acetonitrile–0.1% TFA, using the gradient method). After purification, the combined fractions were lyophilized. The product (**BEEF-CP,** 88 mg, yield 82%) was isolated as a yellow powder in high purity (>98%, HPLC).

^{1}H NMR (600 MHz, DMSO-d_6) δ 12.38 (bs, 2H), 10.41 (s, 1H), 10.12 (bs, 2H), 8.58 (s, 1H) 8.32 (dd, *J* = 8.0, 1.7 Hz, 1H), 7.46 (d, *J* = 2.3 Hz, 1H), 7.40 (d, *J* = 8.1 Hz, 1H), 7.37 – 7.33 (m, 1H), 7.02 – 7.00 (m, *J* = 9.6 Hz, 1H), 6.89 – 6.83 (m, 2H), 6.80 (d, *J* = 8.8 Hz, 1H), 6.78 – 6.75 (m Hz, 1H), 6.72 (s, 2H), 6.40 (s, H), 4.32 – 4.26 (m, 4H), 4.06 (s, 8H), 2.37 (ddp, *J* = 21.7, 14.7, 7.4 Hz, 4H), 0.94 (t, *J* = 7.5 Hz, 6H). ^{13}C NMR (151 MHz, DMSO-d_6) δ 172.4, 168.2, 163.4, 158.2, 158.0, 145.0, 149.5, 149.5, 139.4, 136.6, 135.6, 132.8, 127.3, 121.5, 121.2, 118.5, 118.4, 115.0, 113.5, 108.6, 107.5, 101.8, 67.2, 67.1, 53.5, 22.4, 14.2. HR-MS:(ESI) calcd. for $C_{47}H_{44}N_3O_{16}$ 906.27161 [M+H]$^+$, found 906.27247. HR-ESI-MS-MS (CID=40%; rel. int. %): 848(100); 816(21); 728(9); 593(9) and 433(3).

Spectroscopic characterization of BEEF-CP

Single-photon absorption and emission of BEEF-CP

UV-Vis absorption spectra in the wavelength range of 220–700 nm were recorded on a Thermo Scientific Evolution 220 spectrometer in a quartz cuvette (pathlength = 1.0 cm). The general sample preparation protocol involved the dissolution of 1–3 mg of the studied compounds in 50.0 mL MOPS buffer (pH = 7.20) and then a dilution to the suitable concentrations ($1.0–8.8\times10^{-5}$ M) based on the UV-Vis absorption properties of the compounds. The spectrum of the pure solvent was subtracted from the sample spectra. Using the corrected spectra, the molar absorbance (ε) at a specific wavelength value was calculated, and the position of a selected absorption band (λ_{max}) and the full molar absorption spectra of the compounds were determined.

Fluorescence emission data were measured on a Hitachi F-4500 spectrophotometer in a quartz cell with 1.0 cm pathlength. Slit widths were selected to provide 5 nm and 10 nm bandpass for the excitation and emission beams, respectively. Emission data were normalized to the intensity of a probe body, as an external reference, recorded every day. Solvent background spectra (emission spectra or excitation scans) were subtracted from the spectra of samples. Sample solutions were prepared by dilutions from the stock solutions used for the recording of the UV/Vis absorption spectra. The final concentration of the solutions prepared for fluorescence experiments was around 5 μM. The compounds were excited at their absorption maxima (λ_{max}) determined during UV/Vis absorption measurements. Quantum yields were calculated by ratiometric method.[10]

Table S6. Spectroscopic parameters of **BEEF-CP** without Ca^{2+} or in the presence of calcium ion in aqueous media.

	Absorption		Emission			
	l_{max} (nm)	ε ($M^{-1}cm^{-1}$)	$l_{EM,max}$ (nm)	I_{max} (mM)	QY [a]	$l_{EM,max} - l_{EX}$ (nm)
**BEEF-CP** [b]	504	58600	534	252	0.141	30
**[BEEF-CP – Ca^{2+}]** [c]	504	57900	535	917	0.548	31

[a]Quantum yields (QY) were determined using fluorescein in HEPES buffer (pH = 7.4) as a reference.
[b]The measurements are carried out in water using MOPS buffer (pH = 7.20) and 100 mM KCl in the presence of EGTA (10.0 mM).
[c]The measurements are carried out in water using MOPS buffer (pH = 7.20) and 100 mM KCl in the presence of EGTA (10.0 mM) and $CaCl_2$ (10.0 mM).

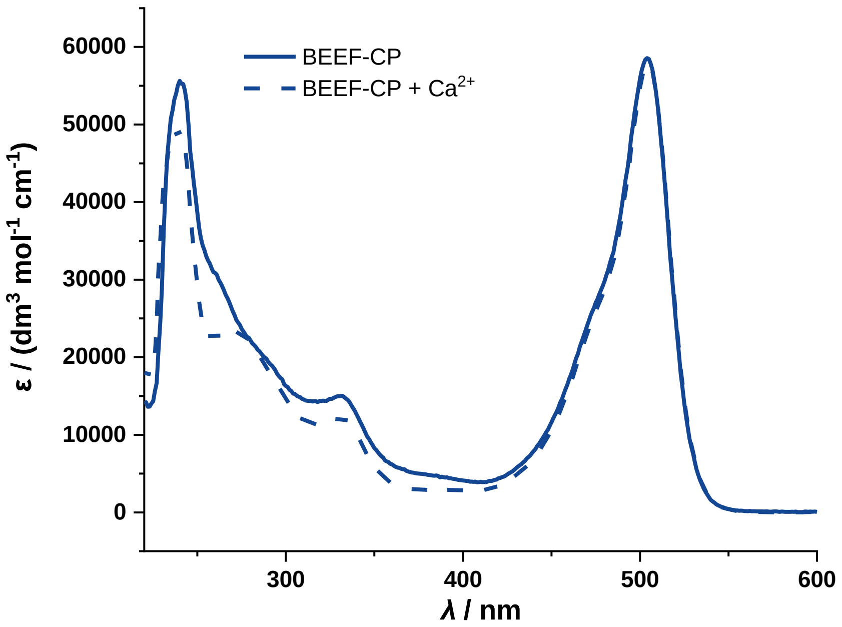

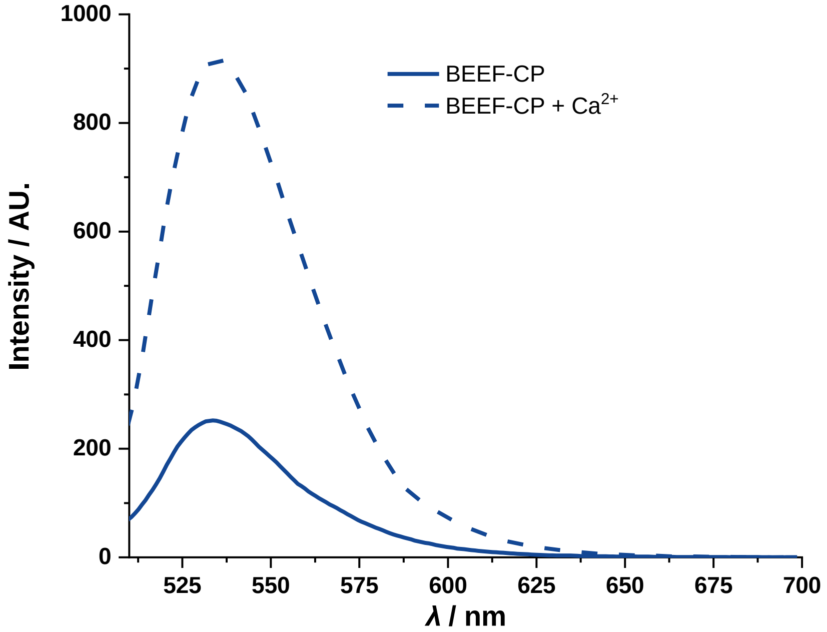

Fig. S18. Comparison of the molar UV-Vis absorption (top) and fluorescence emission (bottom) spectra (normalized to c = 1.0 µM concentrations) of BEEF-CP in water using MOPS buffer (pH = 7.20) and 100 mM KCl in the presence of EGTA (10.0 mM) without calcium ion (solid line) or in the presence of Ca^{2+} (10.0 mM) (dashed line). Excitation was carried out at 504 nm.

Determination of dissociation constant (K_d) of fluorescent calcium indicator BEEF-CP

Fluorescence-monitored Ca^{2+}-titrations were executed by using a solution of the selected dye molecule containing the Ca^{2+}-buffer EGTA in a concentration of 0.01 M but no Ca^{2+} ions. These starting solutions were made by the dilution of the stock solution of the dye into 'Buffer A'. Another solution, containing the dye in the same concentration, was also prepared by using 'Buffer B'. This latter solution contained the Ca^{2+} buffer EGTA and Ca^{2+} ions at identical, 0.01 M concentration, setting the free Ca^{2+} level to 37 μM. For the measurement of the first (0 M) point of the titration curve 2.0 mL of the sample made with 'Buffer A' was used. In the titration process various (typically increasing) volume fractions of the sample were replaced by the same volumes of the solution made with 'Buffer B'. Fine details of this fluorescence titration protocol have been described previously [11,12]. The recorded spectra are shown in Fig. S14 and the fluorescence intensities versus the log$[Ca^{2+}]_{free}$ are shown in Fig. S16. The K_d value of **BEEF-CP** determined using this method is 175.6 nM.

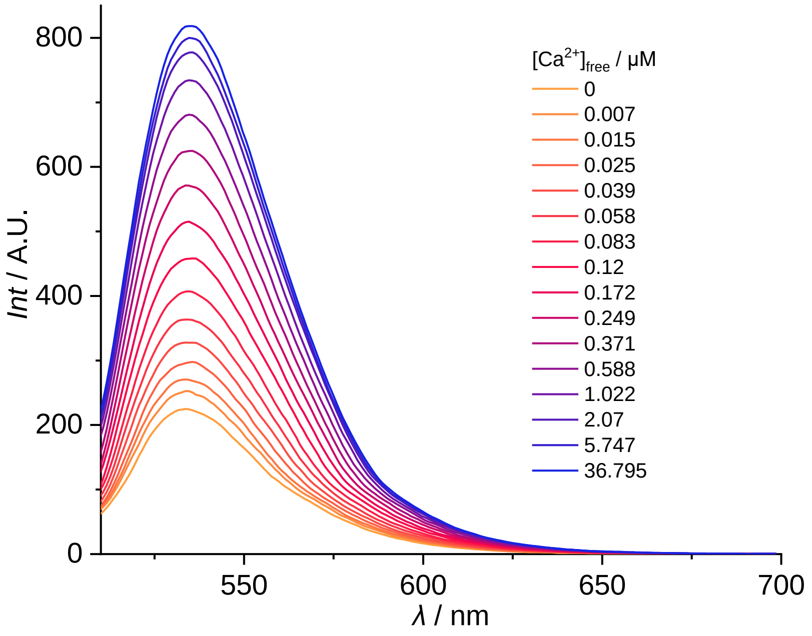

Fig. S19. Emission spectra of BEEF-CP recorded in the presence of different free Ca^{2+} concentrations in MOPS buffer (pH = 7.2) containing 100 mM KCl.

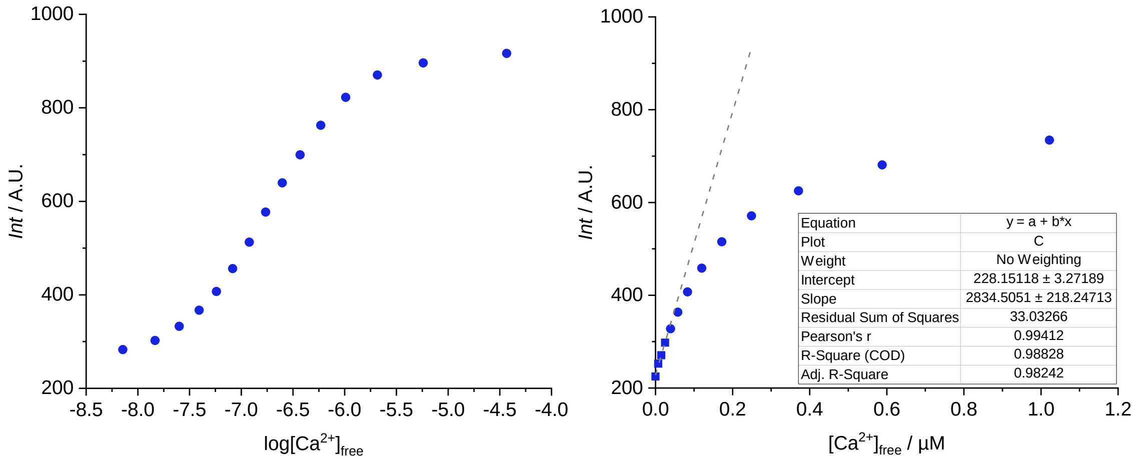

Fig. S20. Calcium titration curves used for the determination of the dissociation constant (K_d) constant of the calcium complex of compound **BEEF-CP.** At free Ca^{2+} concentrations much lower than Kd, the intensity changes linearly with the free Ca^{2+} concentration. The data points used for the linear fitting are indicated with squares in the right panel.

pH sensitivity of the fluorescent sensor BEEF-CP

0.8 mg of **BEEF-CP** was dissolved in DMSO to obtain a stock solution. pH Buffers for the range of 3–9 based on NaOAc/AcOH (pH 3–5) or Na-HEPES/HEPES (pH 6–9) were prepared in the concentration of 5 mM. The ionic strength was set to 50 mM by the addition of KCl and either 1.5 mM EGTA or 1.5 mM $CaCl_2$ was dissolved in the solution. The fluorescent spectra of **BEEF-CP** have been recorded using a Shimadzu 1900 spectrophotometer in each Ca^{2+}-containing and Ca^{2+}-free pH buffer. The used excitation wavelength was 504 nm, both the excitation and emission slits were set to 5 nm. The spectra were recorded at a scan speed of 2000 nm/min by recording datapoints every in 0.5. nm. The instrument was used in low sensitivity mode. The emission intensity values were plotted against for pH in both the Ca^{2+}-containing and Ca^{2+}-free solutions shown in Fig. S18.

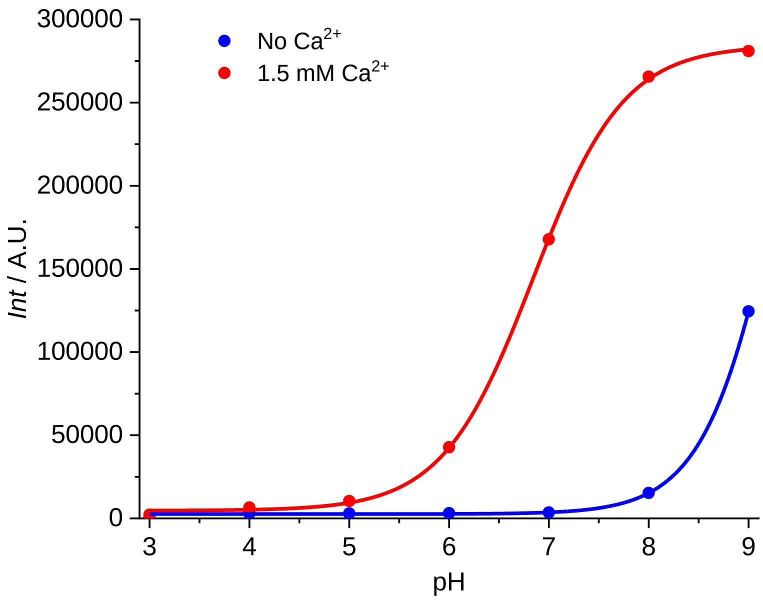

Fig. S21. Emission intensity of probe BEEF-CP measured in solutions containing no Ca^{2+} or 1.5 mM Ca^{2+} at different pH values.

Ion-selectivity of the fluorescent sensor BEEF-CP

The fluorescence spectra of 50 mM pH = 7.4 HEPES solutions containing 1 mM of different metal salts (NaCl, KCl, $MgCl_2$, $CaCl_2$, $Zn(OTf)_2$, $MnCl_2$, $FeCl_2$, $FeCl_3$, $CuSO_4$, $NiCl_2$) and 10 µM BEEF-CP were recorded. The free solution contained 1 mM EGTA to capture any free ion in the solution. The area under the curve of the Ca^{2+} containing solution was normalized and the areas under the curve of the other solutions were compared to that as shown in Fig. **S22**. The selectivity of the sensor is similar to other BAPTA-type sensors, mainly showing turn-on for Zn^{2+} and Mn^{2+}.

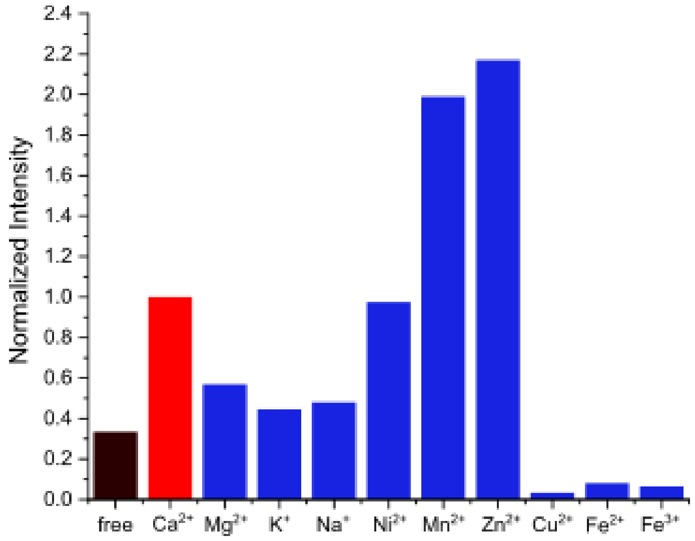

Fig. S22. Normalized emission intensity of BEEF-CP measured in aqueous solutions (pH = 7.4 HEPES) containing 1 mM of different metal salts. (NaCl, KCl, $MgCl_2$, $CaCl_2$, $Zn(OTf)_2$, $MnCl_2$, $FeCl_2$, $FeCl_3$, $CuSO_4$, $NiCl_2$)

Two-photon action cross-section measurements

The two-photon action cross section (TPCS) was determined with the two-photon excited fluorescence (TPEF) method [13]. The measurements were performed using an inverted two-photon microscope (FemtoSmart2D, Femtonics), equipped with a XLUMPFLN20XW Olympus objective (numerical aperture; NA = 1.0) and a tunable high-power Ti:Sapphire laser (Coherent Chameleon Ultra II, wavelength of the excitation light is between 700 nm and 1050 nm). The incident light source was focused into at capillary filled with either the sample or the reference solution (Rhodamine 6G in MeOH [14]) and integrated fluorescence emission was detected in a wavelength window from 500 to 550 nm (green channel of the microscope). The power of the laser source was kept constant at 32 mW. TPCS at each excitation wavelength was calculated according to the following equation:

$$TPCS_{sam} = TPCS_{ref} \cdot \frac{A_{sam} \cdot c_{ref} \cdot a_{ref} \cdot n_{D,ref}}{A_{ref} \cdot c_{sam} \cdot a_{sam} \cdot n_{D,sam}}$$

where A is the mean TPEF emission intensity, c is the concentration of the dye, n is the refractive index of the solvent measured at the sodium D-line; a is a ratio derived from one-photon emission measurements calculated as the integral of one-photon emission spectrum from 500 to 550 nm divided by the total one-photon emission spectrum integral, *ref* is reference, *sam* is sample. The dye concentrations were 10 μM, in pH = 7.4 HEPES (50 mM). To investigate the Ca^{2+} complex the dye was studied in a solution also containing 1 mM $CaCl_2$ and to study the free dye a soltion containing 1 mM EGTA was used. The 2P action cross section spectra resemble the literature spectra of fluoresceins, with a slight batochromic shift.[15] As shown in Fig. S2, the brightness with constant modulation was the highest at 700 nm in the biological sample, which may be explained by the sample itself and the performance profile of the light source. Since the dye has one of its two-photon absorption peaks at this wavelength, we chose it for the biological experiments. At 700 nm the two-photon signal is dominating the signal, as for single photon image the spatial resolution would be degraded (see **Fig**. S1e).

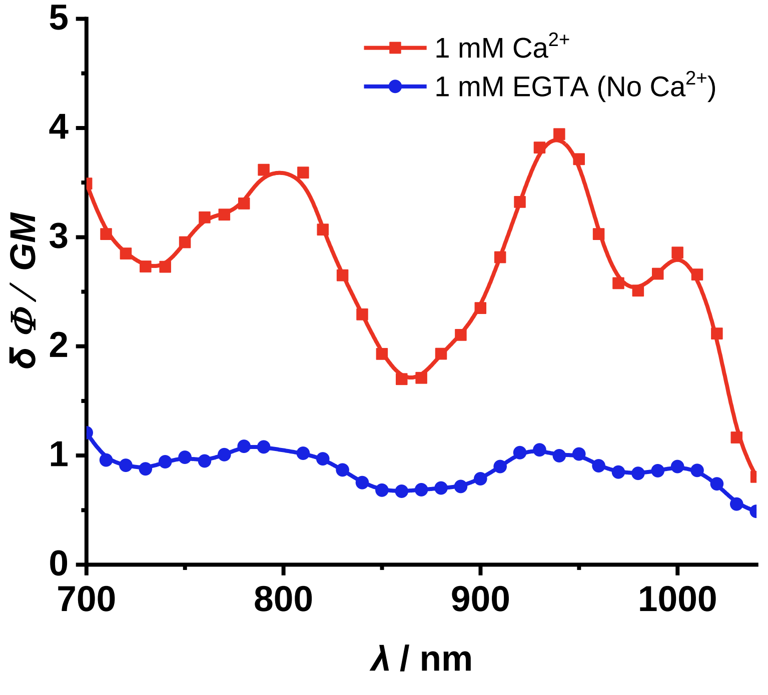

Fig. S23. Two-photon cross section of BEEF-CP in HEPES buffer at pH = 7.4 in the presence of 1 mM Ca^{2+} (red) or without Ca^{2+} in the presence of 1 mM EGTA. GM: Goeppert-Mayer unit.

NMR spectra of the synthesized novel compounds

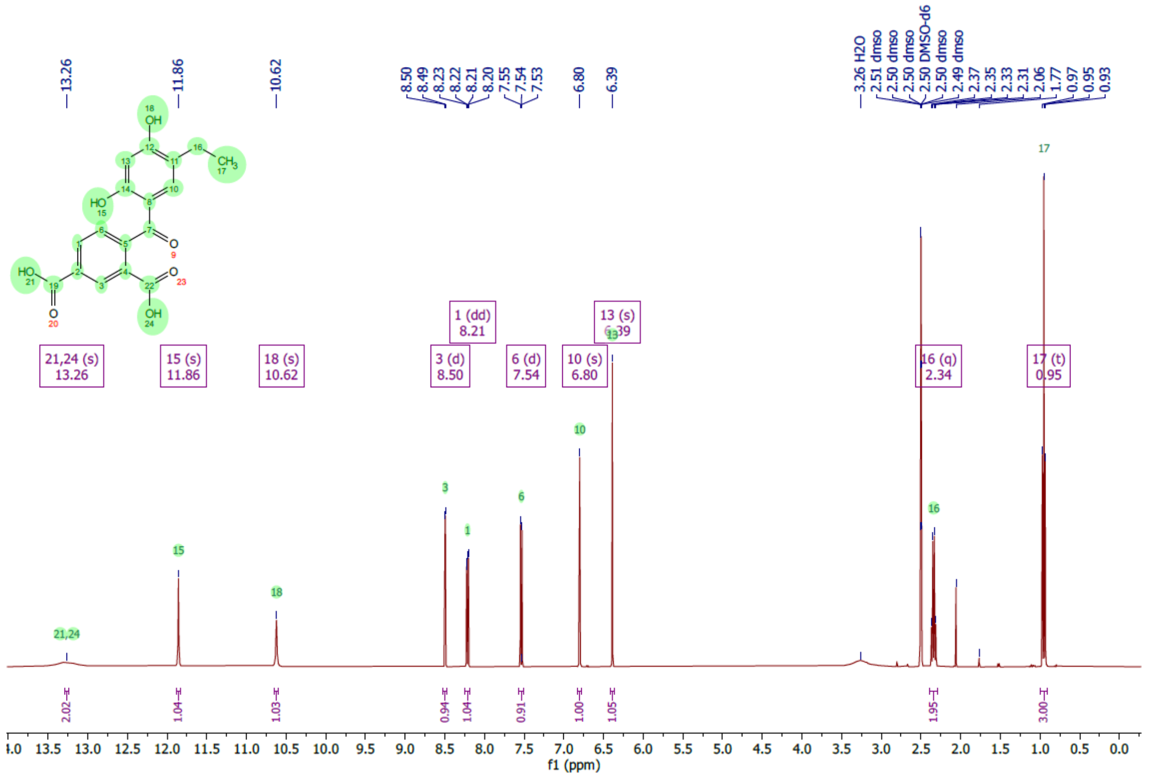

Fig. S24. ^{1}H NMR spectrum of **5** recorded at 400 MHz in DMSO-d6.

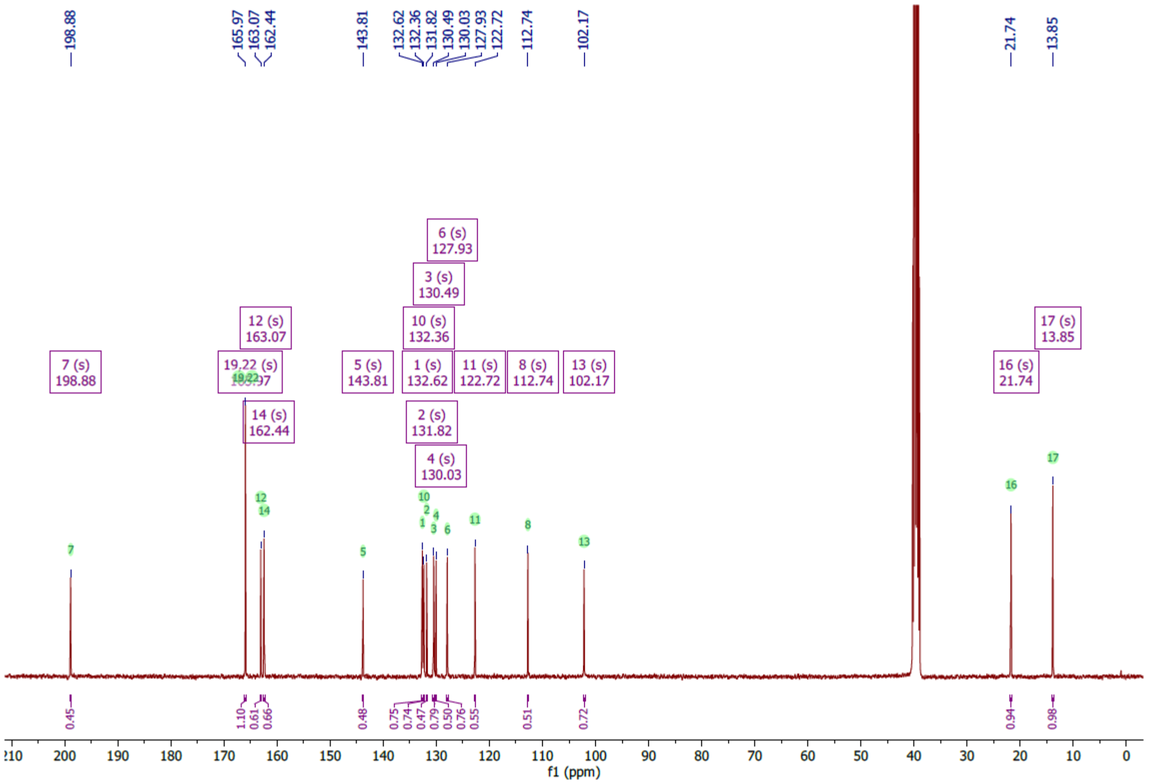

Fig. S25. ^{13}C NMR spectrum of **5** recorded at 101 MHz in DMSO-d6.

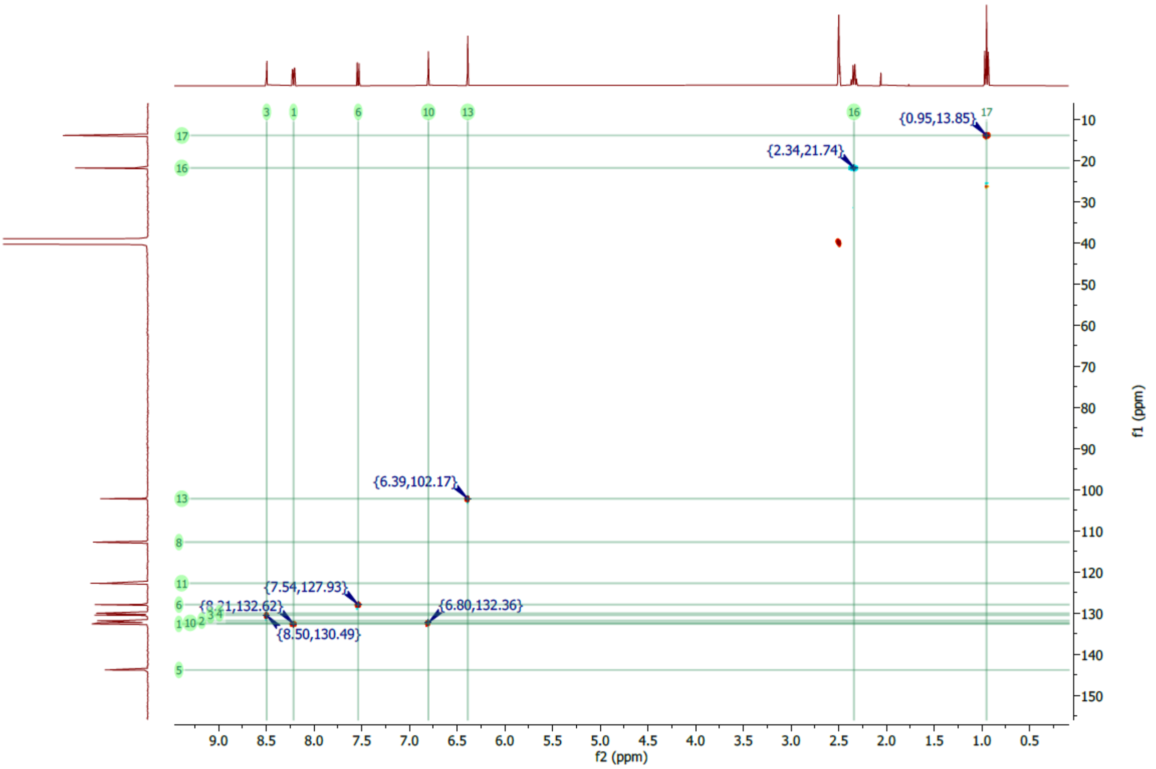

Fig. S26. HSQC spectrum of **5** recorded in DMSO-d$_6$.

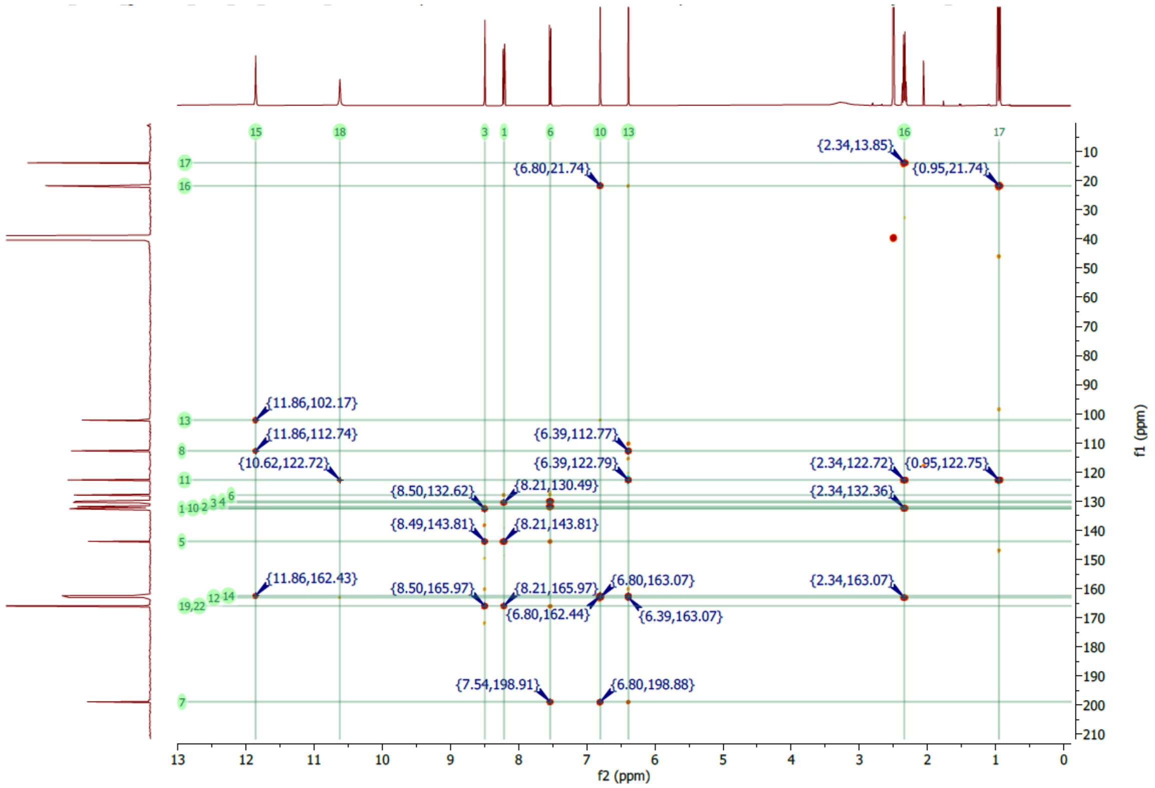

Fig. S27. HMBC spectrum of **5** recorded in DMSO-d$_6$.

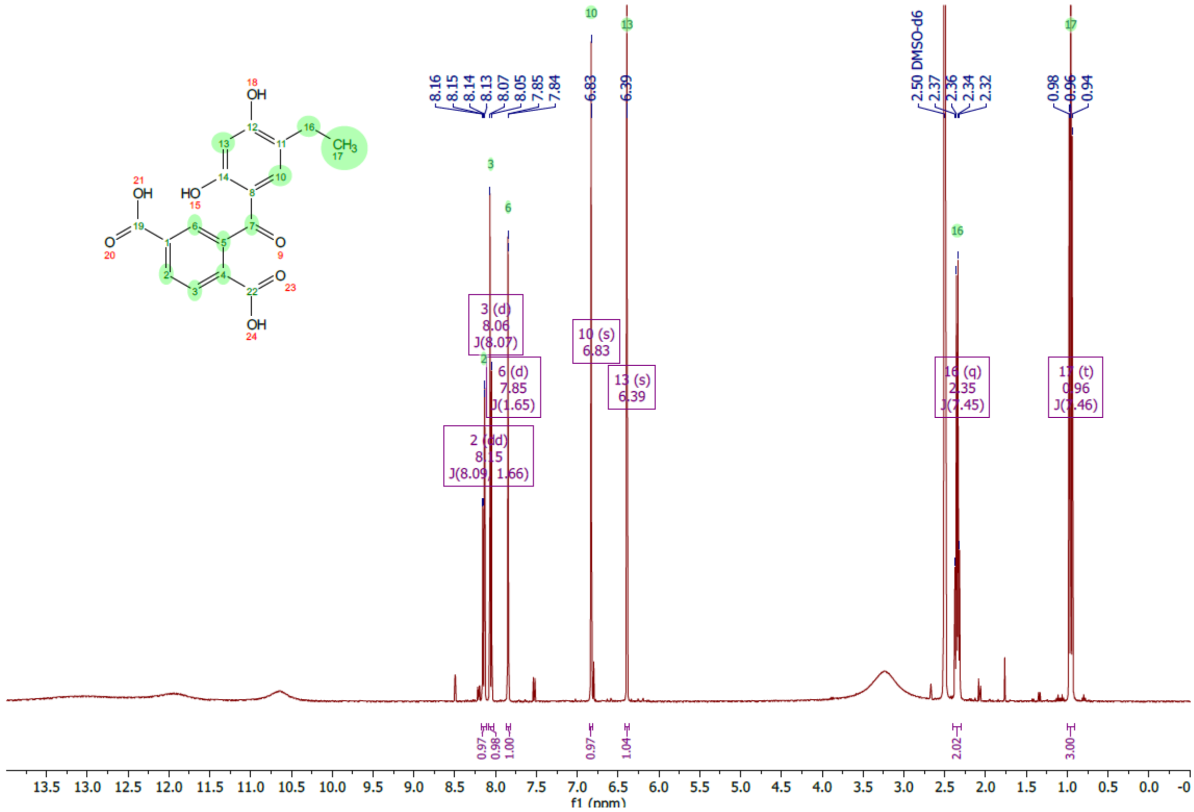

Fig. S28. ^{1}H NMR spectrum of **5_co** recorded at 400 MHz in DMSO-d_6.

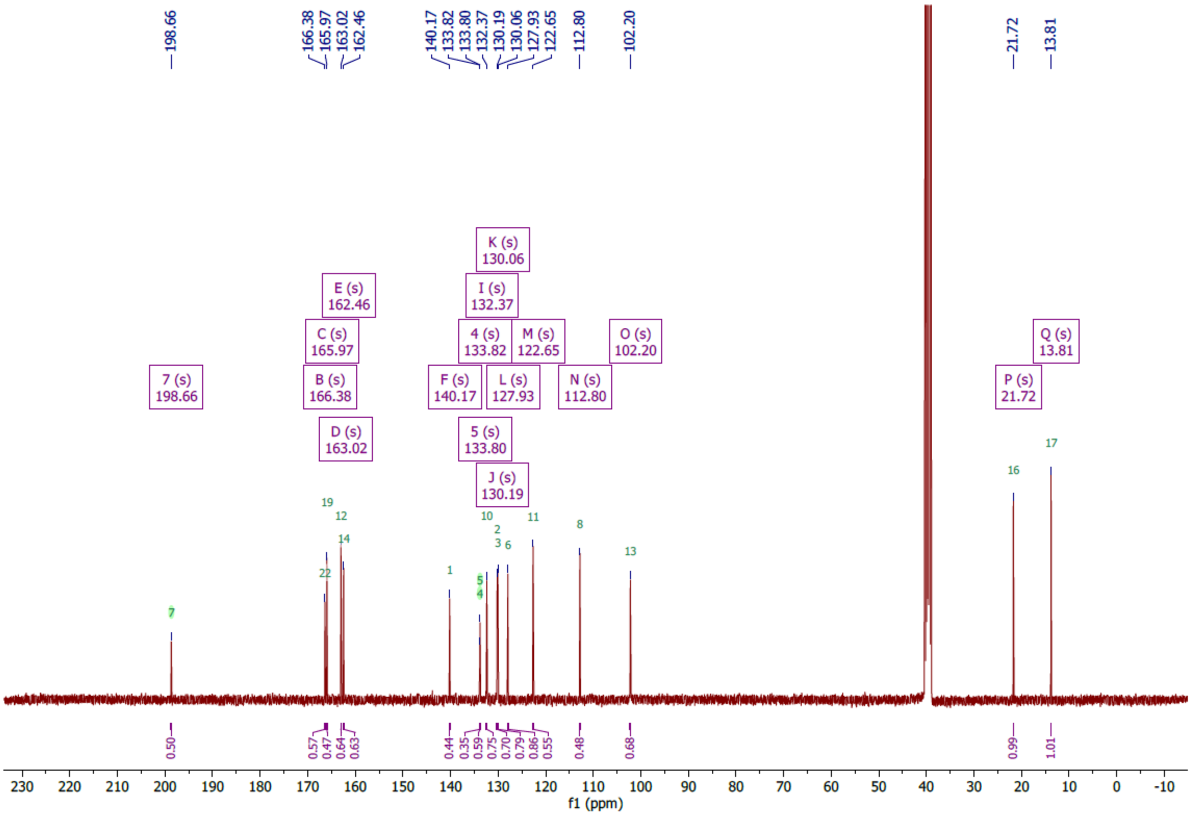

Fig. S29. ^{13}C NMR spectrum of **5_co** recorded at 101 MHz in DMSO-d_6.

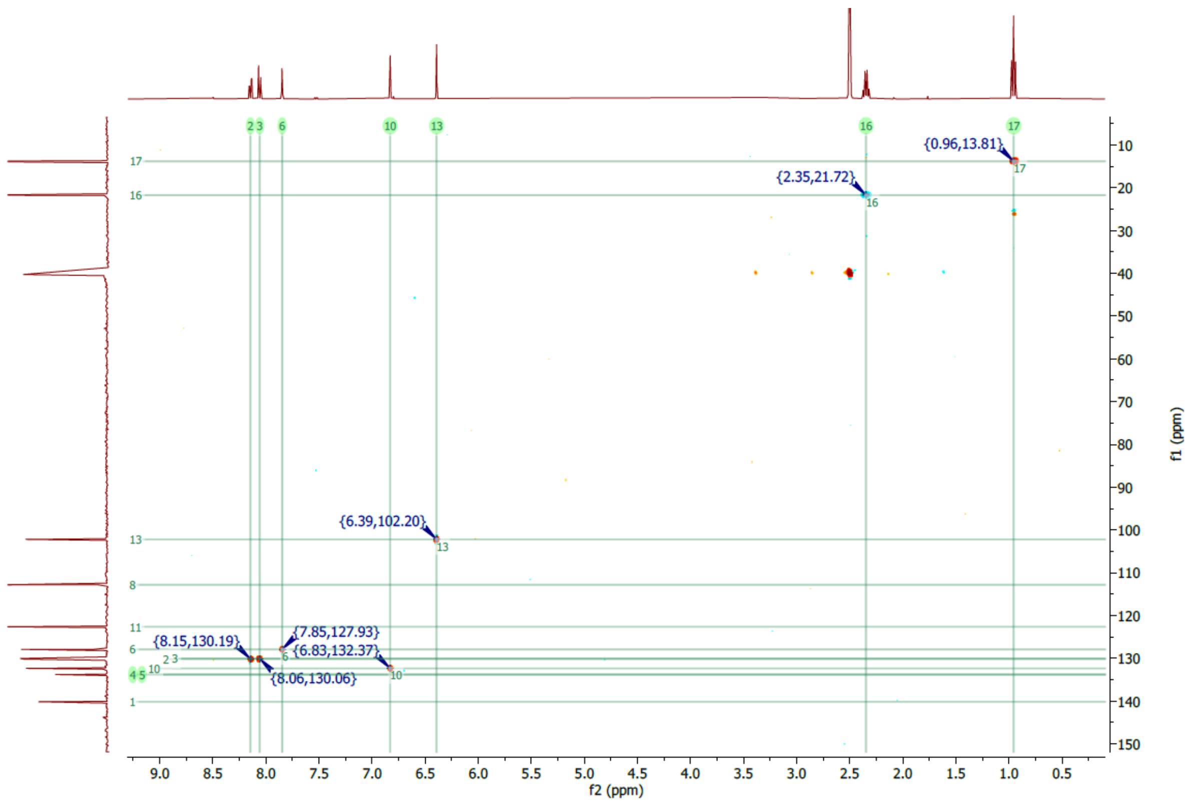

Fig. S30. HSQC spectrum of **5_co** in DMSO-d$_6$.

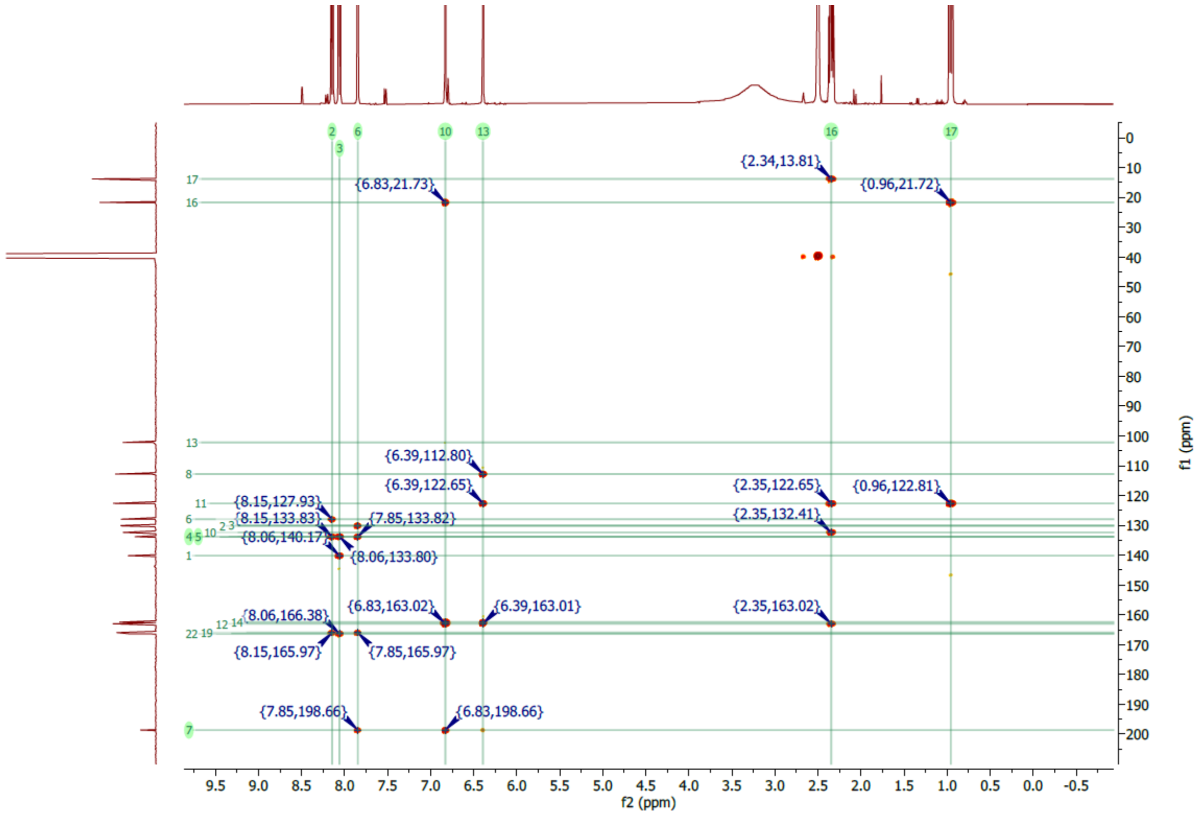

Fig. S31. HMBC spectrum of **5_co** in DMSO-d$_6$.

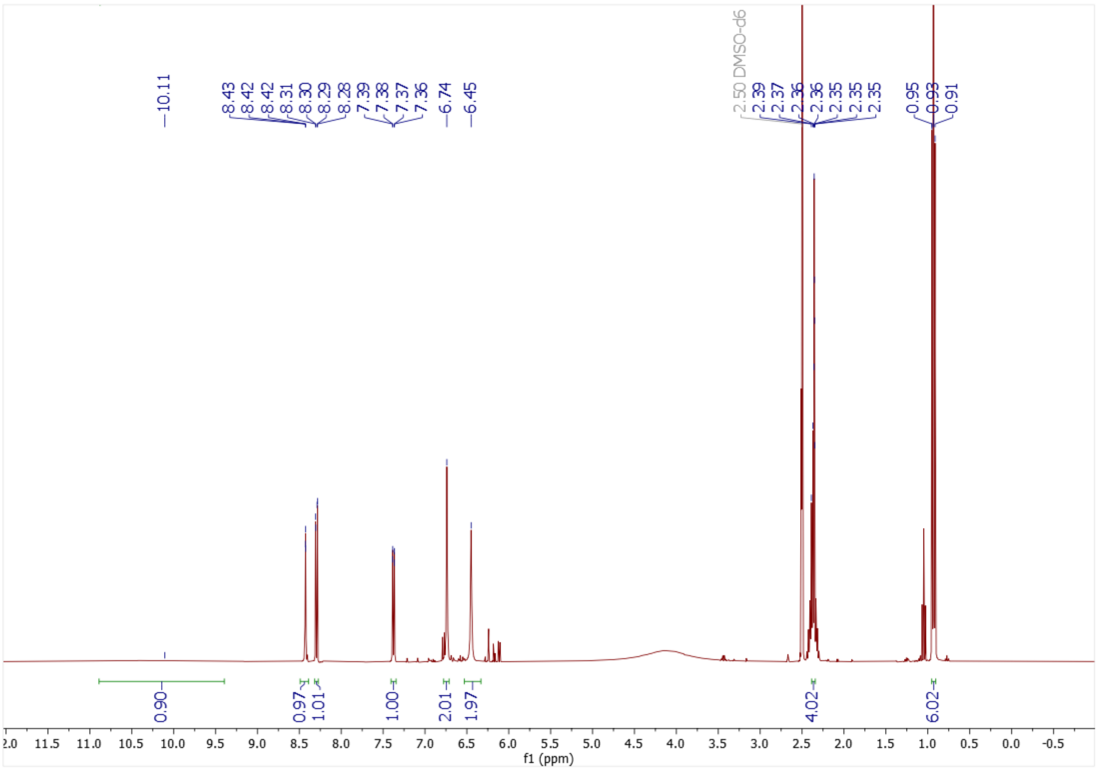

Fig. S32. ^{1}H NMR spectrum of **6** recorded at 400 MHz in DMSO-d$_6$.

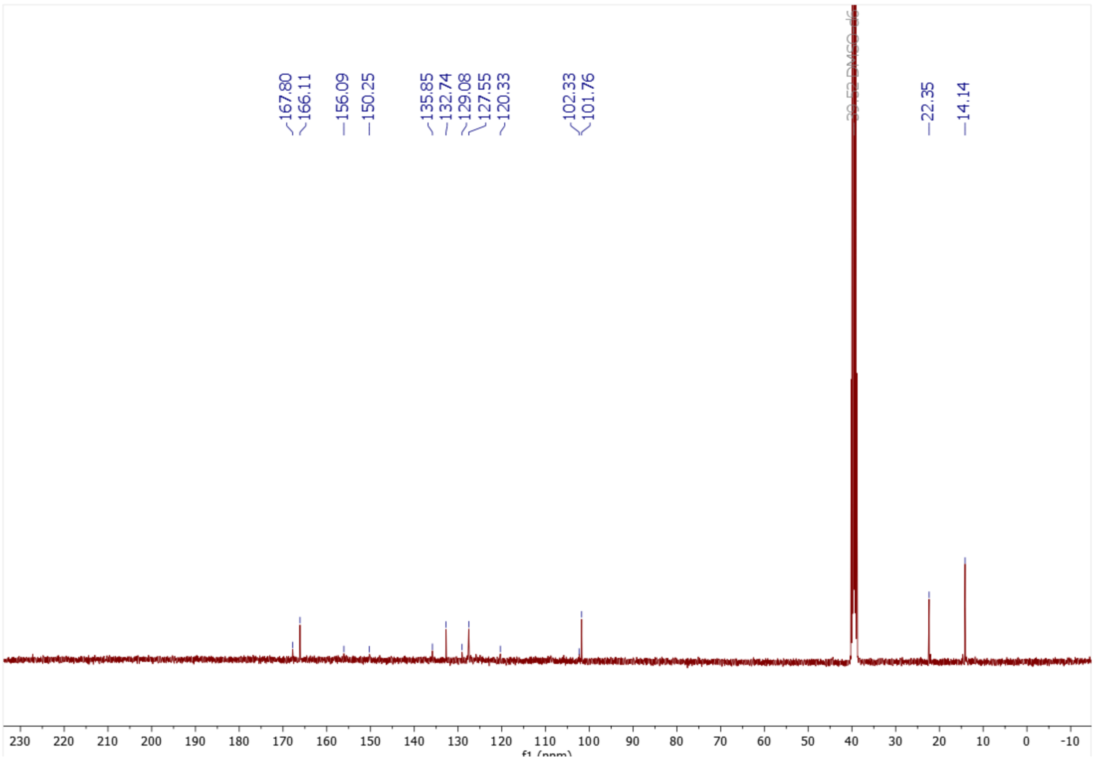

Fig. S33. ^{13}C NMR spectrum of **6** recorded at 101 MHz in DMSO-d$_6$.

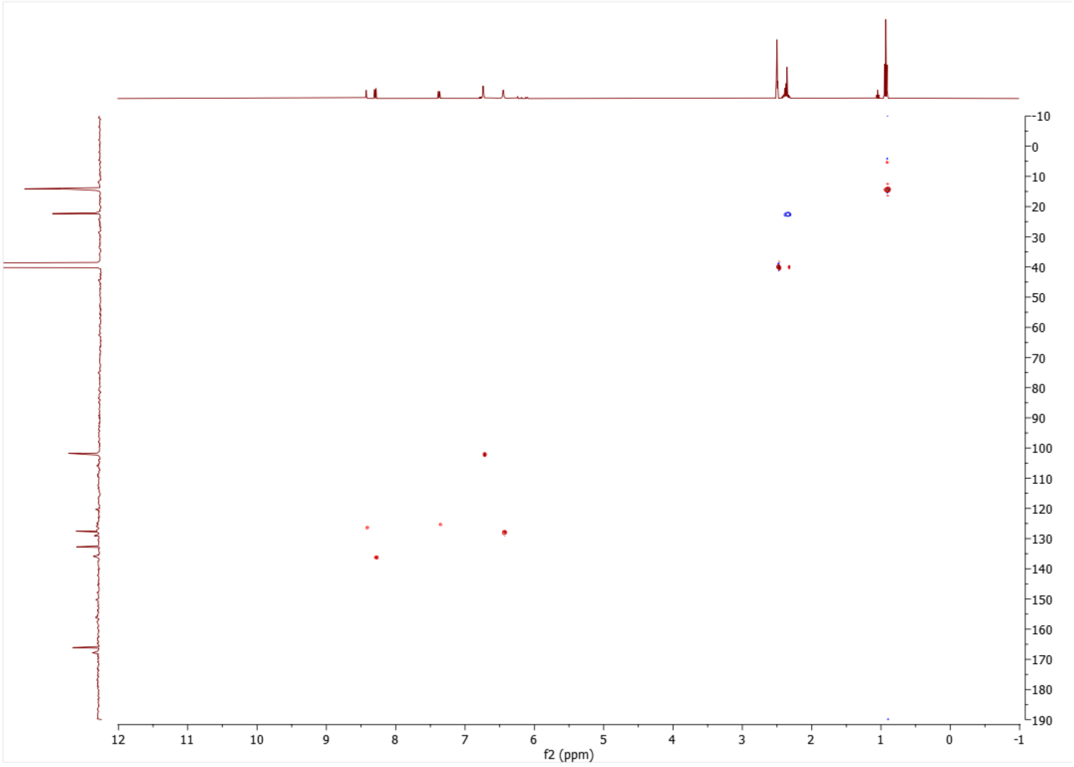

Fig. S34. HSQC spectrum of **6** recorded in DMSO-d6.

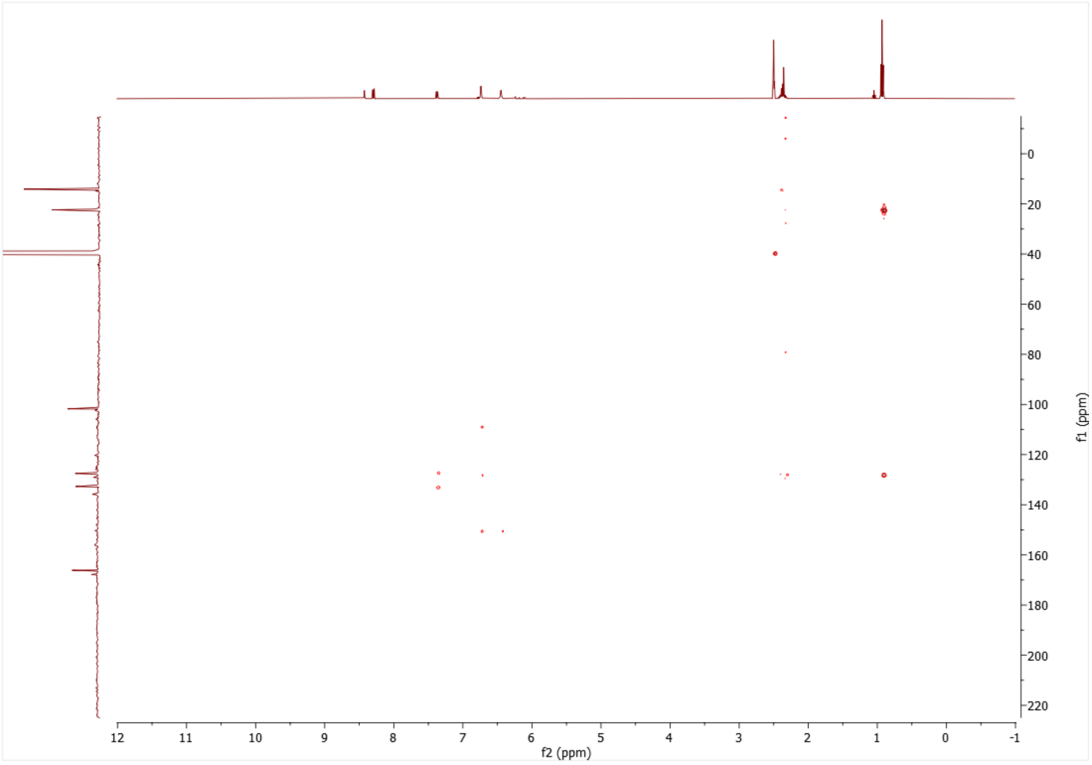

Fig. S35. HMBC spectrum of **6** recorded in DMSO-d6.

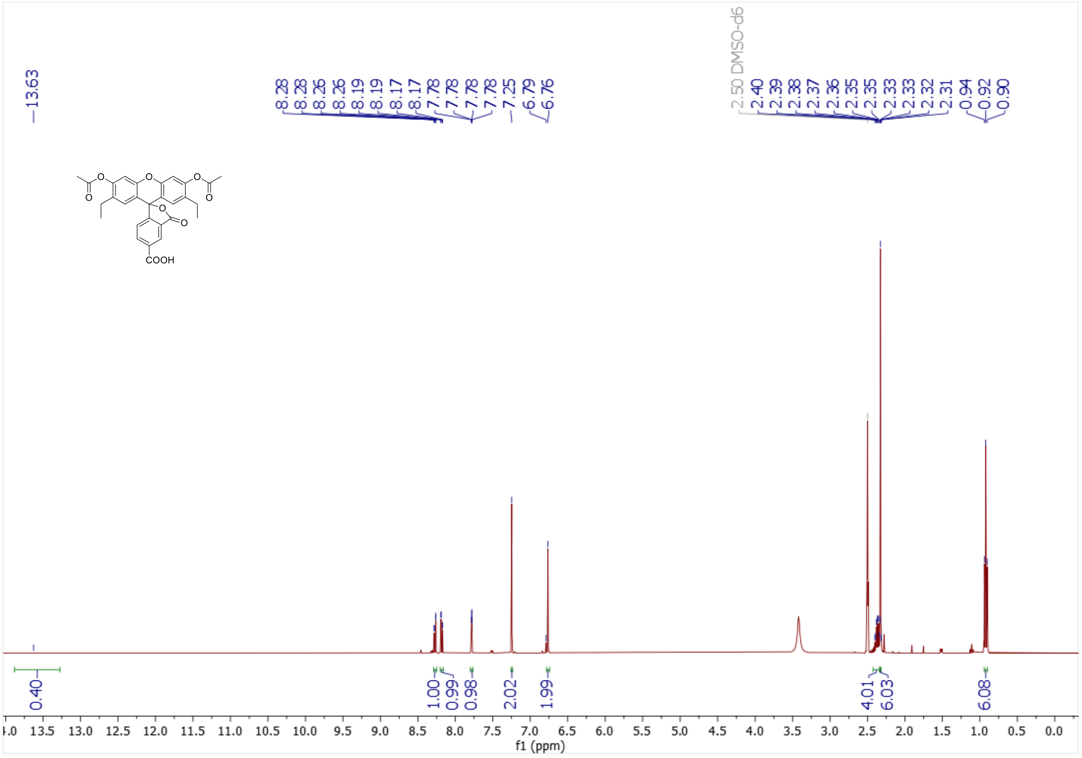

Fig. S36. ^{1}H NMR spectrum of **3** recorded at 400 MHz in DMSO-d_6.

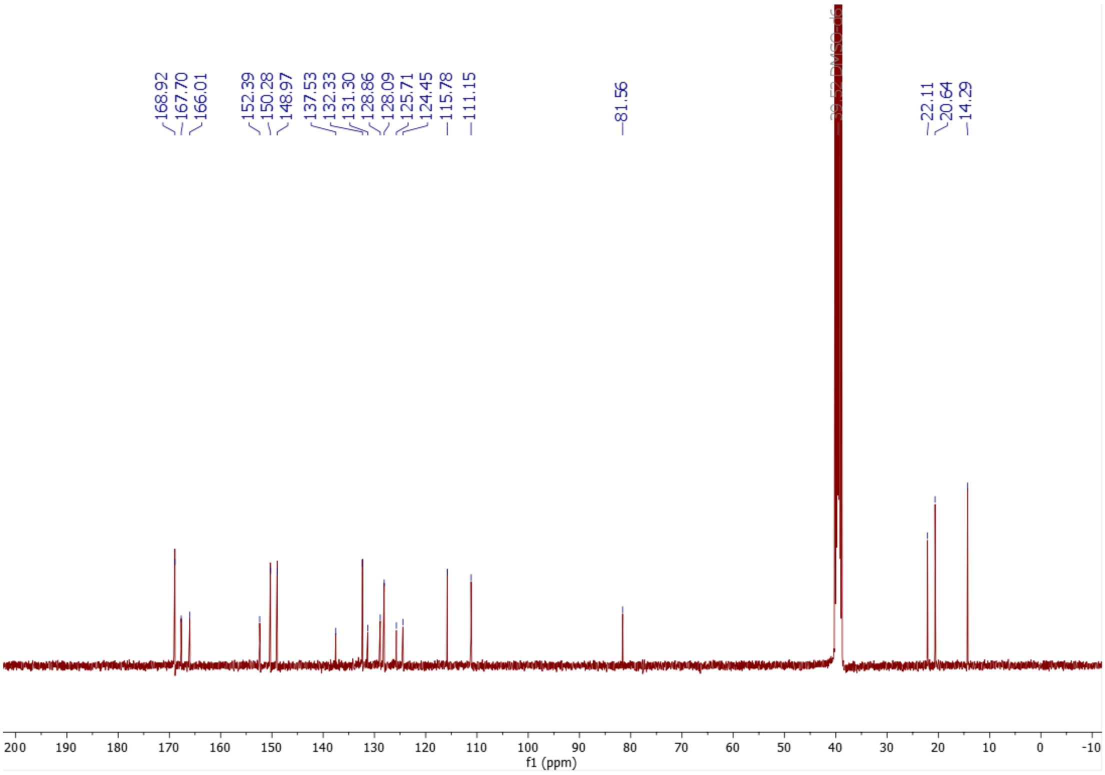

Fig. S37. ^{13}C NMR spectrum of **3** recorded at 101 MHz in DMSO-d_6.

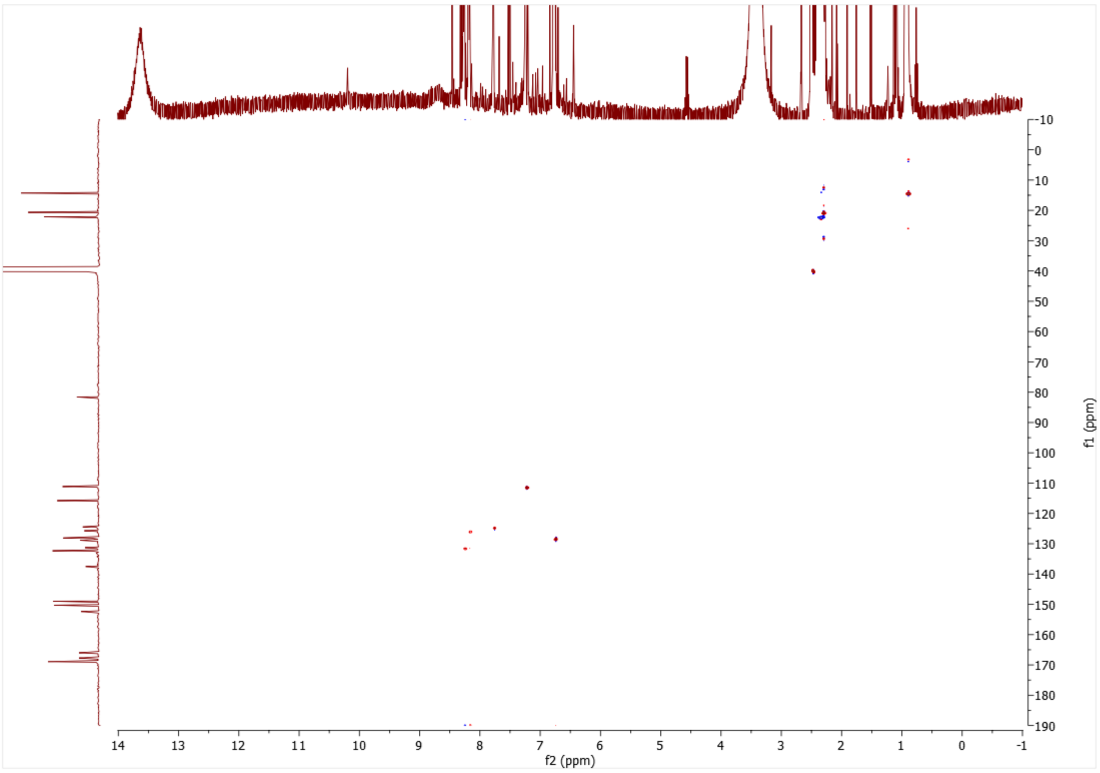

Fig. S38. HMBC spectrum of **3** recorded in DMSO-d_6.

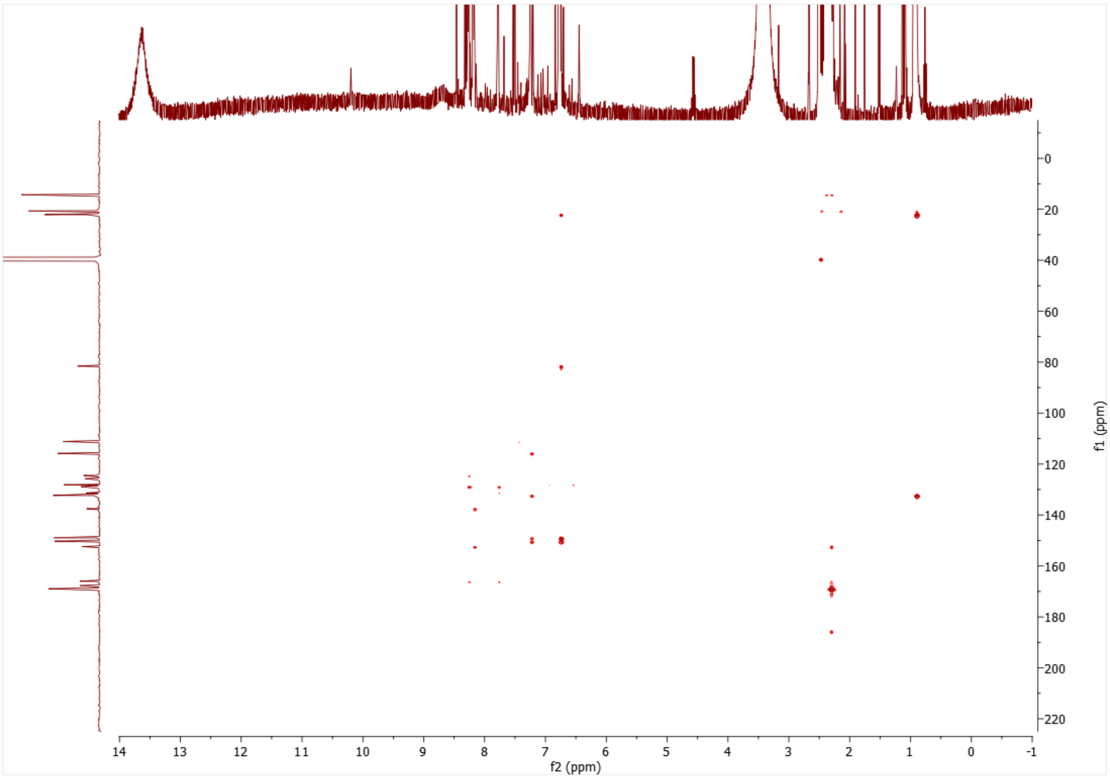

Fig. S39. HSQC spectrum of **3** recorded in DMSO-d_6.

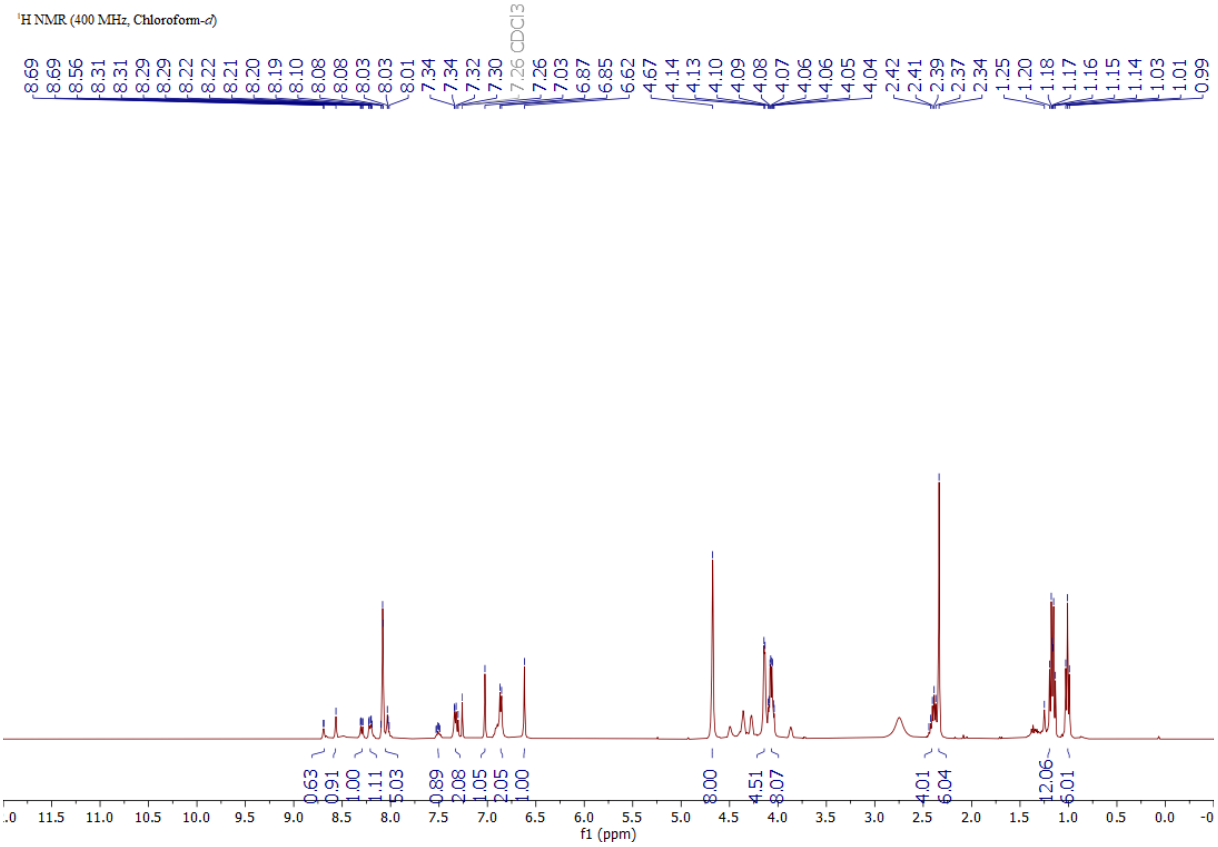

Fig. S40. ^{1}H NMR spectrum of **7** recorded at 400 MHz in $CDCl_3$.

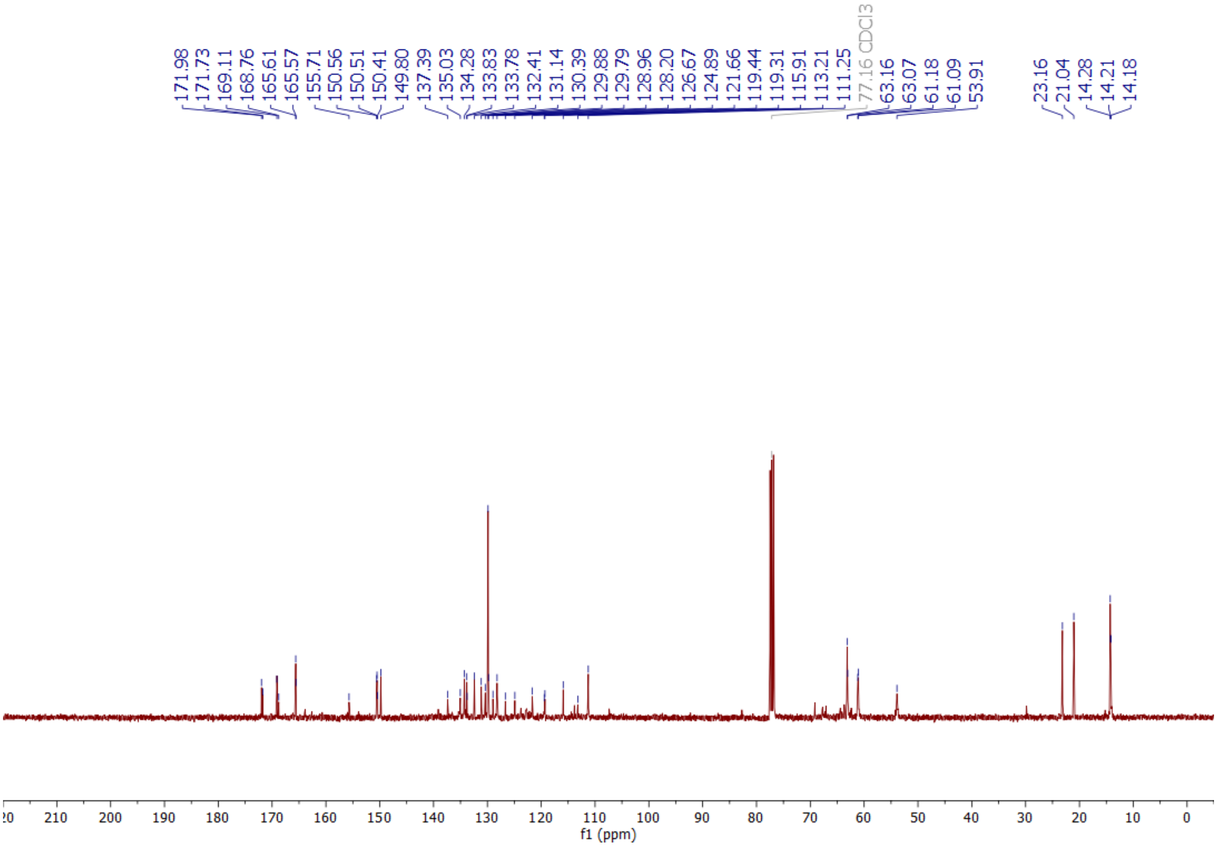

Fig. S41. ^{13}C NMR spectrum of **7** recorded at 101 MHz in $CDCl_3$.

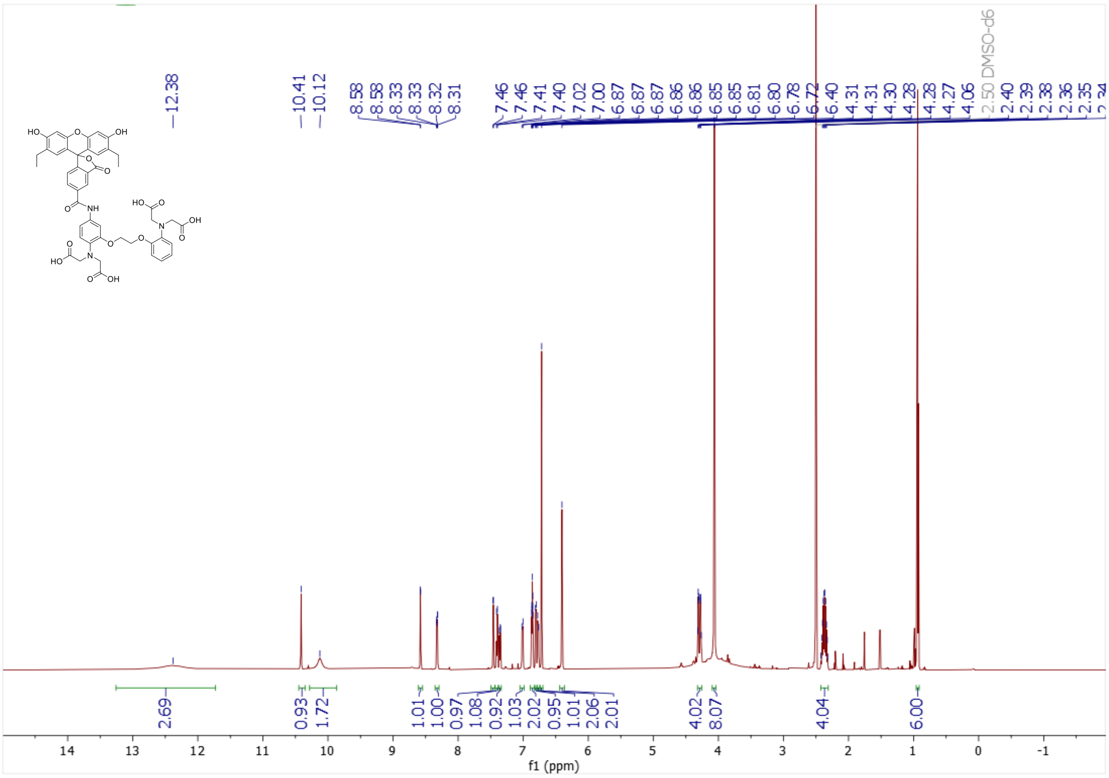

Fig. S42. ^{1}H NMR spectrum of **BEEF-CP** recorded at 600 MHz in DMSO-d_6.

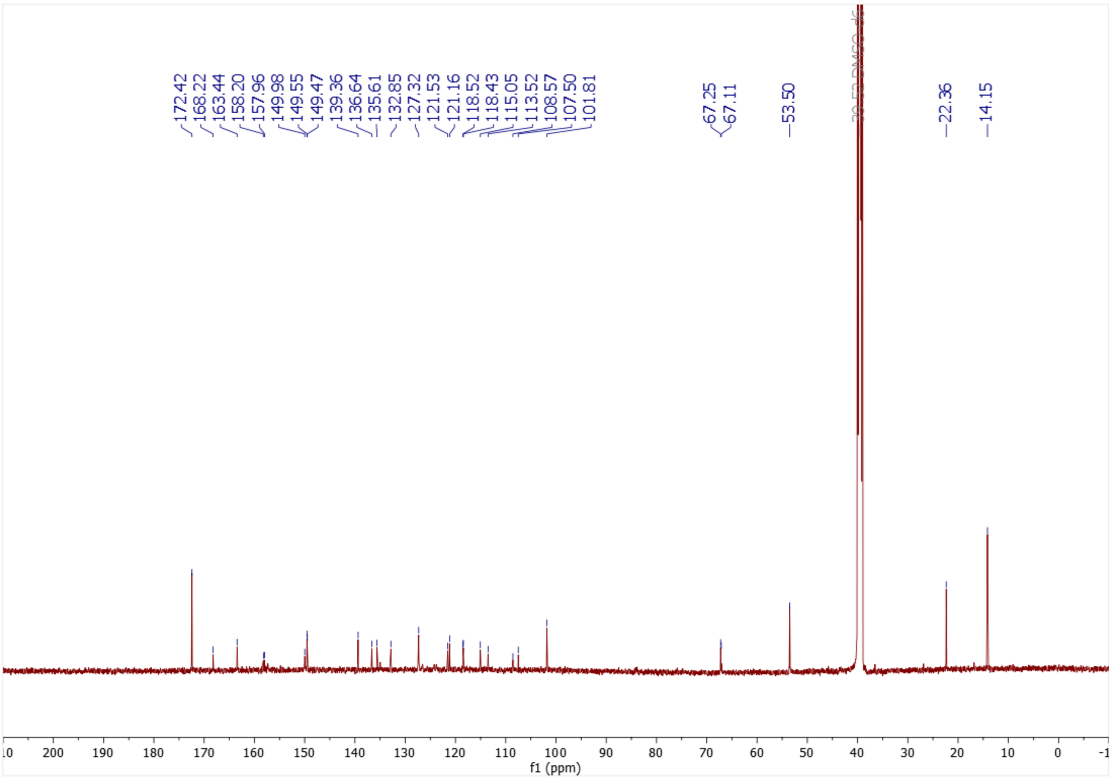

Fig. S43. ^{13}C NMR spectrum of **BEEF-CP** recorded at 151 MHz in DMSO-d_6.

Supplementary data references

(1) Domokos, M.; Szalay, G.; Cseri, L.; Mucsi, Z.; Rózsa, B. J.; Kovács, E. Dataset S1 _ Monitoring Correlates of SARS-CoV-2 Infection in Cell Culture Using Two-Photon Calcium Imaging. *Mendeley Data* **2022**, *V1*, doi: 10.17632/296576fp6h.1. https://doi.org/10.17632/296576fp6h.1.

(2) Plaze, M.; Attali, D.; Prot, M.; Petit, A.-C.; Blatzer, M.; Vinckier, F.; Levillayer, L.; Chiaravalli, J.; Perin-Dureau, F.; Cachia, A.; Friedlander, G.; Chrétien, F.; Simon-Loriere, E.; Gaillard, R. Inhibition of the Replication of SARS-CoV-2 in Human Cells by the FDA-Approved Drug Chlorpromazine. *Int. J. Antimicrob. Agents* **2021**, *57* (3), 106274. https://doi.org/10.1016/j.ijantimicag.2020.106274.

(3) Clarisse, S.-B.; Melissa, T.; Ali, T.; S., O. N.; J., B. P.; K., N. D.; Ying, W.; S., H. P.; J., S. E.; J., van H. M. Suramin Inhibits SARS-CoV-2 Infection in Cell Culture by Interfering with Early Steps of the Replication Cycle. *Antimicrob. Agents Chemother.* **2020**, *64* (8), 10.1128/aac.00900-20. https://doi.org/10.1128/aac.00900-20.

(4) Putlyaeva, L. V; Lukyanov, K. A. Studying SARS-CoV-2 with Fluorescence Microscopy. *International Journal of Molecular Sciences*. 2021, p 6558. https://doi.org/10.3390/ijms22126558.

(5) Lukose, J.; Chidangil, S.; George, S. D. Optical Technologies for the Detection of Viruses like COVID-19: Progress and Prospects. *Biosens. Bioelectron.* **2021**, *178*, 113004. https://doi.org/10.1016/j.bios.2021.113004.

(6) Ju, X.; Zhu, Y.; Wang, Y.; Li, J.; Zhang, J.; Gong, M.; Ren, W.; Li, S.; Zhong, J.; Zhang, L.; Zhang, Q. C.; Zhang, R.; Ding, Q. A Novel Cell Culture System Modeling the SARS-CoV-2 Life Cycle. *PLOS Pathog.* **2021**, *17* (3), e1009439. https://doi.org/10.1371/journal.ppat.1009439.

(7) Rato, S.; Golumbeanu, M.; Telenti, A.; Ciuffi, A. Exploring Viral Infection Using Single-Cell Sequencing. *Virus Res.* **2017**, *239*, 55–68. https://doi.org/10.1016/j.virusres.2016.10.016.

(8) Csomos, A.; Kontra, B.; Jancsó, A.; Galbács, G.; Deme, R.; Kele, Z.; Rózsa, B. J.; Kovács, E.; Mucsi, Z. A Comprehensive Study of the Ca^{2+} Ion Binding of Fluorescently Labelled BAPTA Analogues. *Eur. J. Org. Chem.* **2021**, *2021* (37), 5248– 5261. https://doi.org/10.1002/ejoc.202100948.

(9) Csomos, A.; Kontra, B.; Jancsó, A.; Galbács, G.; Deme, R.; Kele, Z.; Rózsa, B. J.; Kovács, E.; Mucsi, Z. A Comprehensive Study of the Ca^{2+} Ion Binding of Fluorescently Labelled BAPTA Analogues. *Eur. J. Org. Chem.* **2021**, *2021* (37), 5248– 5261. https://doi.org/10.1002/ejoc.202100948.

(10) Lakowicz Joseph R. Effects of Solvents on Fluorescence Emission Spectra. In *Principles of Fluorescence Spectroscopy*; Springer: Boston, 2006; pp 187–215. https://doi.org/https://doi.org/10.1007/978-0-387-46312-4.

(11) Tsien, R.; Pozzan, T. T. Measurement of Cytosolic Free Ca^{2+} with $Quin_2$. *Methods Enzymol.* **1989**, *172* (C), 230–262. https://doi.org/10.1016/S0076-6879(89)72017-6.

(12) Chelators, Calibration Buffers, Ionophores and Cell-Loading Reagents. In *The Molecular Probes® Handbook - A Guide to Fluorescent Probes and Labeling Technologies 11th Edition*; 2010.

(13) Xu, C.; Webb, W. W. Measurement of Two-Photon Excitation Cross Sections of Molecular Fluorophores with Data from 690 to 1050 Nm. *J. Opt. Soc. Am. B* **1996**, *13* (3), 481–491. https://doi.org/10.1364/JOSAB.13.000481.

(14) Makarov, N. S.; Drobizhev, M.; Rebane, A. Two-Photon Absorption Standards in the 550–1600 Nm Excitation Wavelength Range. *Opt. Express* **2008**, *16* (6), 4029–4047. https://doi.org/10.1364/OE.16.004029.

(15) Makarov, N. S.; Drobizhev, M.; Rebane, A. Two-Photon Absorption Standards in the 550-1600 Nm Excitation Wavelength Range. *Opt. Express* **2008**, *16* (6), 4029. https://doi.org/10.1364/oe.16.004029.
